# Tuning of G-CSFR signaling by *de novo*-designed agonists

**DOI:** 10.1016/j.ymthe.2025.08.031

**Published:** 2025-08-29

**Authors:** Timo Ullrich, Christoph Pollmann, Malte Ritter, Jérémy Haaf, Narges Aghaallaei, Ivan Tesakov, Valeriia Hatskovska, Maya El-Riz, Kateryna Maksymenko, Sergey Kandabarau, Maksim Klimiankou, Claudia Lengerke, Karl Welte, Birte Hernandez-Alvarez, Patrick Müller, Andrei Lupas, Jacob Piehler, Julia Skokowa, Mohammad ElGamacy

**Affiliations:** 1 Max Planck Institute for Biology, Department Protein Evolution, 72076 Tübingen, Germany; 2 Friedrich Miescher Laboratory of the Max Planck Society, 72076 Tübingen, Germany; 3 Department of Biology/Chemistry and Center for Cellular Nanoanalytics, Osnabrück University, 49076 Osnabrück, Germany; 4 Internal Medicine II, University Hospital Tübingen, 72076 Tübingen, Germany; 5 German Cancer Consortium (DKTK) partner site Tübingen, a partnership between DKFZ and University Hospital Tübingen, Tübingen, Germany; 6 University Children’s Hospital Tübingen, 72076 Tübingen, Germany

**Keywords:** *de novo* protein design, cytokine therapy, hematopoietic stem cells, G-CSF, G-CSFR, tuning cytokine signaling, granulopoiesis, neutropenia, therapeutic proteins

## Abstract

Enhancing cytokine-based therapies by systematically tuning how an agonist associates its receptor is emerging as a powerful new concept in drug discovery. Here, we report the design and characterization of agonists that tune granulocyte-colony-stimulating factor receptor (G-CSFR) activity, which is central for the proliferation and granulocytic differentiation of hematopoietic stem cells. Using design agonists, we study the impact of varying the receptor-binding affinity and dimerization geometry on receptor association, downstream signaling, and cellular response. Hence, we achieved agonists with altered signaling specificities that are hyper-thermostable, can outcompete the native ligand (G-CSF), and bias cells toward granulopoietic differentiation over triggering proliferation. Furthermore, the design agonists differentially modulate the kinetics and amplitudes of signal transduction pathways and gene expression patterns. In contrast to G-CSF, they achieve more selective activation of gene sets with hematopoietic functions, with minimal unwanted effects on immunomodulatory signaling. These findings demonstrate the potential of dissecting the complex G-CSFR signaling, and they open up ways for new therapeutic applications for designed cytokines.

## Graphical abstract

**Figure:**
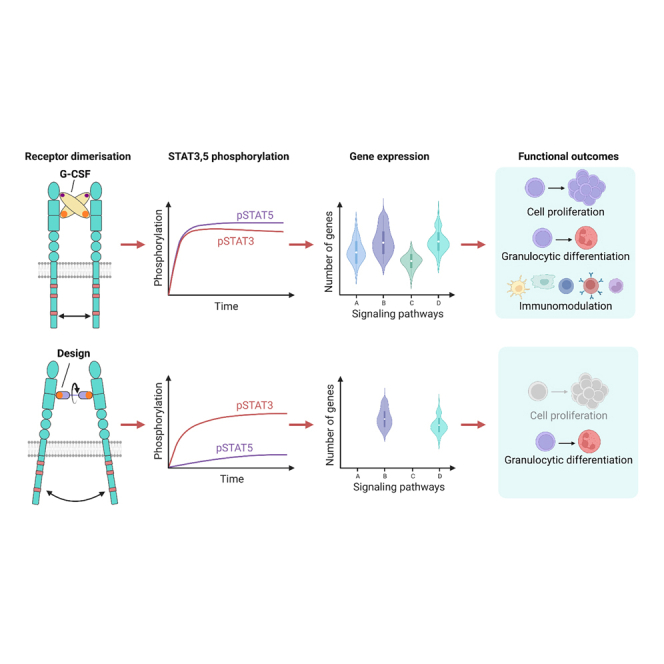

## Teaser

Skokowa, ElGamacy, and colleagues report the design and characterization of protein agonists that fine-tune granulocyte-colony-stimulating factor receptor activity, which is central for the proliferation and granulocytic differentiation of hematopoietic stem cells. The agonists differentially modulate the kinetics and amplitudes of signal transduction pathways and effects on cell proliferation and differentiation.

## Introduction

Cytokine receptor activation typically triggers several signaling pathways that result in different cellular responses, depending on target cells.^1^^,^^2^ Synthetic ligands that engage cytokine receptors in non-native ways can selectively bias the signaling outcomes, overcoming the functional pleiotropy of native agonists.^3^ Modulation of class I/II cytokine receptors is of considerable interest for therapeutic intervention, but this is hampered by the often broad and pleiotropic responses.^4^^,^^5^^,^^6^^,^^7^^,^^8^ Decomposing these signaling outcomes through synthetic agonists can thus unlock previously untapped therapeutic applications.^9^

The observed functional plasticity of natural ligands^10^ has inspired efforts to systematically bias signaling specificity using design agonists purposefully engineered to possess optimal pharmaceutical properties.^9^^,^^11^ For instance, affinity engineering of interleukin-2 (IL-2)^12^ or IL-22^13^ variants possessed immunomodulatory activity or altered tissue specificity when compared to the respective natural ligands. This highlights the effect of the ligand’s receptor-binding affinity on downstream signaling.^14^^,^^15^ In addition to affinity, the association geometry of the receptors can provide another layer of control over downstream signaling,^16^^,^^17^^,^^18^^,^^19^ which was demonstrated through synthetic ligands. Specifically, diabodies and DARPins dimerizing the erythropoietin receptor (EPOR) in non-native orientations could induce a range of differential signaling outcomes.^20^^,^^21^ Alternatively, using antibody fragment fusions, signal tuning was demonstrated for heterodimeric receptor complexes of the IL-2 receptor (IL-2Rβ:γ_c_) and the type I interferon receptor (IFNAR1:IFNAR2).^22^

In this study, we explore the tunability of granulocyte colony-stimulating factor receptor (G-CSFR). Under physiological conditions, the native ligand (G-CSF) homodimerizes G-CSFR to induce signaling, which is crucial for granulopoiesis, and the regulation of hematopoietic stem and progenitor cells (HSPCs) and immune cells.^23^^,^^24^^,^^25^^,^^26^^,^^27^^,^^28^^,^^29^^,^^30^ Recombinant human G-CSF (rhG-CSF) is widely used to treat inherited or chemotherapy-induced neutropenia and for hematopoietic stem cell mobilization.^31^^,^^32^^,^^33^ However, rhG-CSF therapy can cause side effects such as bone pain, splenomegaly, and vasculitis, especially in patients requiring long-term treatment. G-CSF has been shown to activate several types of immunoregulatory cells, including myeloid-derived suppressor cells, dendritic cells, and regulatory T cells.^34^^,^^35^^,^^36^^,^^37^ A variety of cancer and tumor microenvironment cells can also express G-CSFR or secrete G-CSF, including colorectal, breast, lung, ovarian, and pancreatic tumors, glioblastoma, and acute myeloid leukemia.^38^^,^^39^^,^^40^^,^^41^^,^^42^^,^^43^^,^^44^ While the effects of triggering G-CSFR on the deregulation of immune cells or tumor cells are still under investigation, it would beneficial for patients if a G-CSFR ligand could be developed with more specific activity on granulocytic progenitor cells only, to selectively induce granulocytic differentiation, or on HSPCs only, to trigger their mobilization without inducing granulopoiesis or other cell types.

We therefore sought to explore the feasibility of ligand-mediated tuning of G-CSFR signaling, by altering the G-CSFR association modes, whereby beneficial functions are preserved but undesired effects are neglected. Indeed, by designing G-CSFR agonists that associate the receptor subunits at varying affinities and geometries we were able to alter the downstream phosphorylation levels of key signal transducers and activators of transcription (STATs). Based on characterizing the biophysical properties of our design agonists and investigating their impact on receptor dimerization in the plasma membrane, in conjunction with intracellular signal activation, and cellular activity *in vitro* and *in vivo*, we highlight the potential to tune G-CSFR signaling toward differentiation, while avoiding its unwanted non-hematopoietic effects.

## Results

### Computationally guided screening identifies higher-affinity G-CSFR binders

**Figure 1:**
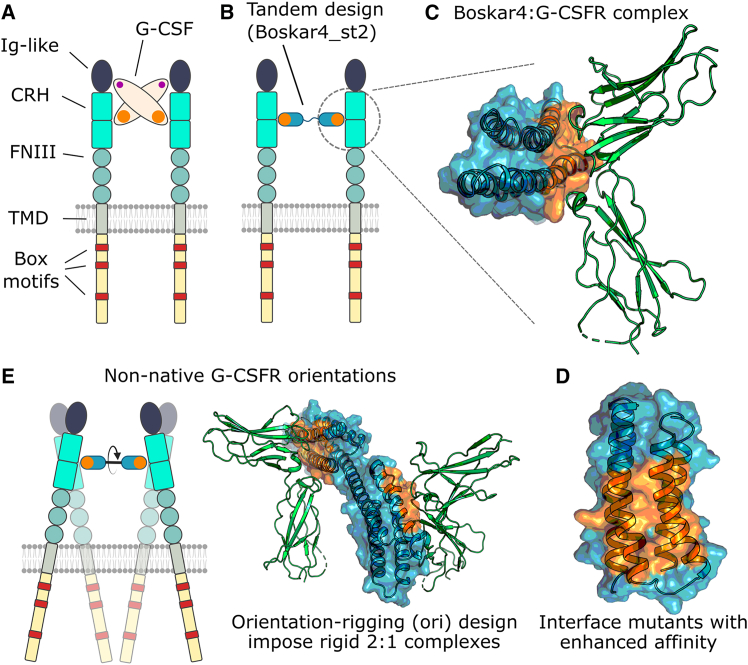
Overall strategy to generate novel G-CSFR modulators with varying affinities and geometries (A) G-CSF (beige oval) activates G-CSFR by dimerizing the Ig-like and CRH domains (dark blue and cyan) through two distinct receptor-binding sites (orange and purple).^45^ (B) In contrast, the *de novo*-designed Boskar4_st2 agonist consists of two tandemly repeated Boskar4 domains, which (C) possess minimal architecture and encode a single high-affinity, receptor-binding site (blue domain and orange surface patch). (D) On the Boskar4 module, the residues forming the receptor-binding entity were diversified in order to identify affinity-enhanced variants. (E) The improved Boskar4 module was fused into rigid, orientation-rigging (ori) designs that can dimerize and activate G-CSFR in non-native geometries.

**Figure 2:**
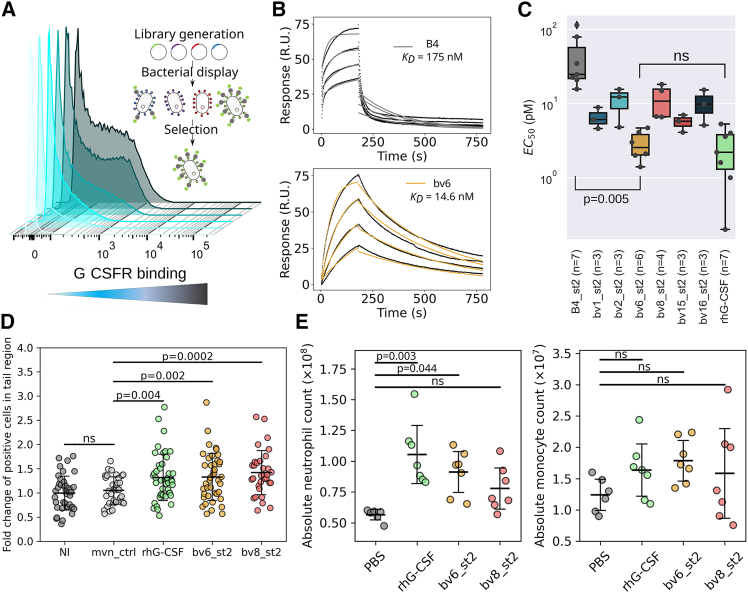
Affinity-enhanced binders exhibit improved granulopoietic activity *in vitro* and *in vivo* (A) Cell sorting of bacterially displayed Boskar4 mutants bound to fluorescently labeled G-CSFR was conducted over five steps of enrichment. Successive fluorescence shifts are shown as light cyan to dark gray distributions. (B) SPR titrations showed single-domain bv6 to bind 12-fold tighter than its Boskar4 counterpart (Figure S7D; Table 1). (C) Proliferative activity assays in NFS-60 cells showed the enhanced activity (EC_50_) of all variants in comparison to Boskar4_st2, where the most active variant bv6_st2 was not significantly different in activity compared to the native ligand (rhG-CSF; light green box). For statistical analysis, a one-way ANOVA over all samples was performed, followed by a Tukey HSD test. Proliferation assays were conducted in at least three (3≤n≤7) independent experiments per variant or rhG-CSF (Table 1; Figure S8). (D) Interval plots of the fluorescent neutrophils in transgenic *Tg:mpxGFP* zebrafish larvae that were either not injected (NI), injected with the inactive protein Moevan_control (mvn_ctrl),^49^ rhG-CSF, or indicated Boskar4 variants (bv6_st2 or bv8_st2) after 24 h of treatment. Data show mean ± standard deviation, where each circle represents one zebrafish larva (NI *n* = 40, mvn_ctr *n* = 35, rhG-CSF *n* = 44, bv6_st2 *n* = 42, bv8_st2 *n* = 32). Statistical analysis was carried out by ordinary one-way ANOVA followed by a Tukey HSD test. Representative images of embryo tails used in this analysis are provided in Figure S11. (E) C57BL/6 Ly5.1 mice were treated (i.p.) with either PBS buffer, rhG-CSF, bv6_st2, or bv8_st2 (each circle indicates one mouse). Absolute cell counts of mouse neutrophils (left image) and monocytes (right image) in the bone marrow of treated mice are shown. Statistical analysis was carried out by ordinary one-way ANOVA with a multiple comparisons test of all experimental drugs to the PBS buffer treatment as a negative control (PBS, *n* = 6; rhG-CSF, *n* = 7; bv6_st2, *n* = 7; bv8_st2, *n* = 7; ns, not significant).

G-CSF dimerizes G-CSFR subunits in a 2:2 stoichiometry through two (high- and low-affinity) binding sites (Figure 1A). Previously, we *de novo* designed a G-CSFR-binding module (Boskar4), that when tandemly linked (i.e., Boskar4 short tandem of 2 domains [B4_st2]) could dimerize and activate G-CSFR (Figure 1B).^46^ In contrast to G-CSF, Boskar4 binds G-CSFR through only one binding site that engages the CRH domain (Figure 1C). Thus, our first aim was to enhance the affinity of the Boskar4 binding module to G-CSFR, which we reasoned could lead to more potent dimerization by the respective design agonists (i.e., B4_st2 variants). We built a computationally guided mutant library by modeling the Boskar4:G-CSFR complex (Figures 1C and 1D) and scanning for energy-minimizing Boskar4 mutations at the binding interface using Damietta software.^47^ In addition to the computationally designed sequence library, we built a site-saturation mutagenesis of the same positions to serve as control (Figure S1). The designed library contained 6 mutations per position, in contrast to an average of 12 mutations per position for the control library. We used a bacterial display screening system^48^ in which the ligand is presented as an extracellular intimin fusion on the surface of the bacterial particles. Upon adding fluorescently labeled G-CSFR, we sorted the bacterial particles for receptor binding using fluorescence-activated cell sorting (FACS) (supplemental methods). We ran both libraries (saturation and designed) through five cycles of FACS enrichment under the same conditions, where shifts in the fluorescence of both populations were observed across the enrichment cycles (Figure 2A). Forty-eight single clones were picked from either library after the third and fifth FACS cycles, amounting to a total of 192 clones. Using on-plate fluorescence- and luminescence-based assays we quantified the relative binding of these clones to the receptor under the same conditions (Figure S2).

The sequences of the top 40 clones picked from both libraries after Sanger sequencing yielded 16 unique Boskar4 variants (Figure S2D). A focused FACS-based analysis for these ligands’ expression level and binding affinity on the surface of *Escherichia coli* (Figure S3) showed the expression-normalized binding affinities to range from a 3- to a 37-fold increase, when compared to the starting Boskar4 clone. To narrow down the number of candidates for subsequent investigations, while maintaining diversity, we chose six variants from the design library based on their estimated affinity, frequency in the screen, or the respective mutation composition (Figures S2D and S3B). The most frequent variant was bv1, while the highest affinity variant was bv2. Four additional variants, bv6, bv8, bv15, and bv16, were chosen based on their middle- or lower-binding affinity profiles. Specifically, variants with polar and charged residues were included with the goal of selecting the most soluble and specific binders. No hits from the site-saturation library were selected for any further due to the predominantly hydrophobic nature of these variants.

We constructed agonists by creating short tandems of these six selected designs (Figure S4), which were expressed and purified from *E. coli* and evaluated by size-exclusion chromatography (SEC) and SDS-PAGE analysis (Figure S5). We used surface plasmon resonance (SPR) to determine the binding affinity and kinetic parameters of the designs to G-CSFR. The SPR results showed that all the six tested variants in their tandem forms bind G-CSFR within the sub-nanomolar affinity range, with up to 7-fold enhanced affinity compared to the starting template B4_st2 (Figure S6; Table 1). The observed affinity improvement was also higher when evaluated for single-domain variants. For instance, the single-domain form (bv6) of the most biologically active agonist (bv6_st2) exhibited a K_D_ of 14 ± 2 nM, in comparison to the single-domain Boskar4 template, with a K_D_ of 173 ± 43 nM (Figures 2B and S7B). Notably, circular dichroism (CD) measurements of bv6 showed the expected strong α-helical signal. Nanoscale differential scanning fluorimetry (nanoDSF) measurements demonstrated that bv6 exhibited hyper-thermostability, similar to Boskar4 (Figures S7C and S7D).

**Table 1:** Binding affinity and activity parameters determined for G-CSFR modulators

	k_a_ (M^−1^ s^−1^)	k_d_ (s^−1^)	Apparent K_D_ (M)	χ^2^ (RU^2^)	EC_50_ (pM) mean ± SD
Boskar4	(3.4 ± 1.7) × 10^4^	(5.4 ± 1.4) × 10^−3^	(1.7 ± 0.4) × 10^−7^	13.9	NA
bv6	(2.4 ± 0.7) × 10^5^	(3.3 ± 0.4) × 10^−3^	(1.4 ± 0.2) × 10^−8^	2.51	NA
Boskar4_st2	(2.3 ± 1.0) × 10^6^	(3.3 ± 0.7) × 10^−3^	(1.6 ± 0.3) × 10^−9^	0.678	33.1 ± 23.6
bv1_st2	(9.7 ± 2.0) × 10^5^	(2.2 ± 0.2) × 10^−4^	(2.4 ± 0.6) × 10^−10^	1.44	6.5 ± 2.2
bv2_st2	(3.5 ± 1.3) × 10^5^	(1.7 ± 0.1) × 10^−4^	(5.3 ± 1.8) × 10^−10^	0.371	10.9 ± 5.5
bv6_st2	(4.9 ± 0.9) × 10^5^	(5.9 ± 0.5) × 10^−4^	(6.4 ± 1.0) × 10^−10^	1.16	2.9 ± 1.3
bv8_st2	(1.8 ± 0.4) × 10^6^	(1.5 ± 0.1) × 10^−3^	(8.3 ± 1.6) × 10^−10^	1.36	11.6 ± 5.9
bv15_st2	(7.4 ± 5.0) × 10^5^	(4.0 ± 0.1) × 10^−4^	(6.5 ± 2.1) × 10^−10^	0.982	5.6 ± 1.5
bv16_st2	(8.1 ± 0.1) × 10^5^	(7.1 ± 0.8) × 10^−4^	(8.8 ± 1.2) × 10^−10^	0.473	10.1 ± 5.1
rhG-CSF (self-made from *E. coli*)^a^	(3.0 ± 0.3) × 10^5^	(4.9 ± 2.8) × 10^−4^	(1.1 ± 1.6) × 10^−9^	4.9	2.6 ± 1.8
Lenograstim (glycosylated, commercial rhG-CSF)	NA	NA	NA	NA	1.0 ± 0.7
ori0	(6.5 ± 6.6) × 10^5^	(2.5 ± 0.8) × 10^−4^	(5.6 ± 2.4) × 10^−10^	1.11	2.8 ± 1.4
ori1	(1.4 ± 2.1) × 10^6^	(1.2 ± 0.3) × 10^−4^	(3.6 ± 2.8) × 10^−10^	0.779	28.9 ± 0.7
ori2	(1.1 ± 0.8) × 10^5^	(4.6 ± 1.3) × 10^−5^	(3.4 ± 1.6) × 10^−10^	2.83	35.6 ± 19.8
ori3	(8.7 ± 5.9) × 10^5^	(2.5 ± 0.1) × 10^−4^	(4.1 ± 0.1) × 10^−10^	2.36	10.6 ± 3.0
ori4	(1.2 ± 0.1) × 10^5^	(7.6 ± 1.4) × 10^−5^	(6.4 ± 1.14) × 10^−10^	1.37	19.3 ± 6.5

The association rate constant (k_a_), the dissociation rate constant (k_d_), and the apparent equilibrium dissociation constant (K_D_) were obtained by surface plasmon resonance against immobilized hG-CSF receptor (compare Figures S6, S7, and S14). The mean and standard deviation (SD) of the half-maximal effective concentration EC_50_ (pM) was obtained from NFS-60 activity assays of at least three biological replicates (compare Figures S8 and S16).

a The SPR parameters of rhG-CSF (self-made purified from *E. coli*) were adapted from Skokowa et al.^46^

### Affinity enhancement yields highly potent G-CSFR agonists with uncompromised thermostability

To evaluate the biological impact of increased affinity, we evaluated the proliferative activity of our design agonists in G-CSF-responsive NFS-60 cells.^50^^,^^51^ All the affinity-enhanced agonist variants induced stronger proliferation than the starting template (B4_st2). Remarkably, the most active design, bv6_st2, had a half-maximal effective concentration (EC_50_) of 2.9 ± 1.3 pM compared to 33 ± 23.6 pM for B4_st2 and 2.6 ± 1.8 pM for rhG-CSF (Figures 2C and S8; Table 1), whereas no differences in the maximum response were noticed among the designs and rhG-CSF (Figure S8E). Furthermore, the tested agonists showed no strong correlations between the proliferative activity (EC_50_) and the binding affinity (K_D_), the association rate constant (k_a_), or the dissociation rate constant (k_d_) (Figure S9). We therefore expect that the biological activity within this narrow range of affinities might be influenced by other factors such as relative stabilities and binding specificity of the different agonists.

CD spectra of all design agonists indicated their strong α-helical nature (Figure S10A), while nanoDSF melting curves highlighted their thermostability, where half of the tested variants (bv1_st2, bv6_st2, and bv8_st2) exhibited no detectable unfolding up to 110°C, while the other three (bv2_st2, bv15_st2, and bv16_st2) exhibited a melting onset at around 100°C. None of the design agonists exhibited increased scattering over the full temperature range (Figures S10B–S10H), highlighting their excellent colloidal stability. Similar results were observed for the single-domain form of the designs (Figure S7D).

These results indicate that the affinity-driven functional enhancement did not alter the biophysical properties of the designs, which are far superior to rhG-CSF. Therefore, we further sought to test whether the design agonists exhibit similarly enhanced activity *in vivo*.

### Boskar4 variants induce neutrophil production in zebrafish embryos and mice

We tested the capacity of two design agonists, bv6_st2 and bv8_st2, to induce granulopoiesis in a fluorescent neutrophil reporter zebrafish transgenic line, *Tg(mpx:GFP)*.^52^ The mpx:GFP zebrafish transgenic line is a widely used model for studying granulopoiesis due to the specific expression of GFP under the myeloperoxidase promoter. Interestingly, the increased number of GFP^+^ neutrophils localized near the caudal hematopoietic tissue (tail region) was comparable between larvae treated with rhG-CSF and bv6_st2 or bv8_st2 at 24 h post-injection (Figures 2D and S11). The number of neutrophils did not significantly increase in the Moevan_control-injected larvae. Moevan is an inert helical protein^49^ expressed and purified under the same conditions.

We additionally treated C57BL/6 Ly5.1 mice with 300 μg/kg rhG-CSF, bv6_st2, or bv8_st2 by intraperitoneal (i.p.) injection every second day with five injections in total. One day after the fifth injection, the number of neutrophils and monocytes in the bone marrow of treated mice was evaluated. The treatment of mice with bv6_st2 but not bv8_st2 induced the production of neutrophils and monocytes at a level comparable to that of rhG-CSF (Figure 2E). These results confirm the capacity of the design agonist to activate G-CSFR *in vivo*.

### Design of agonists altering the relative orientation of G-CSFR

**Figure 3:**
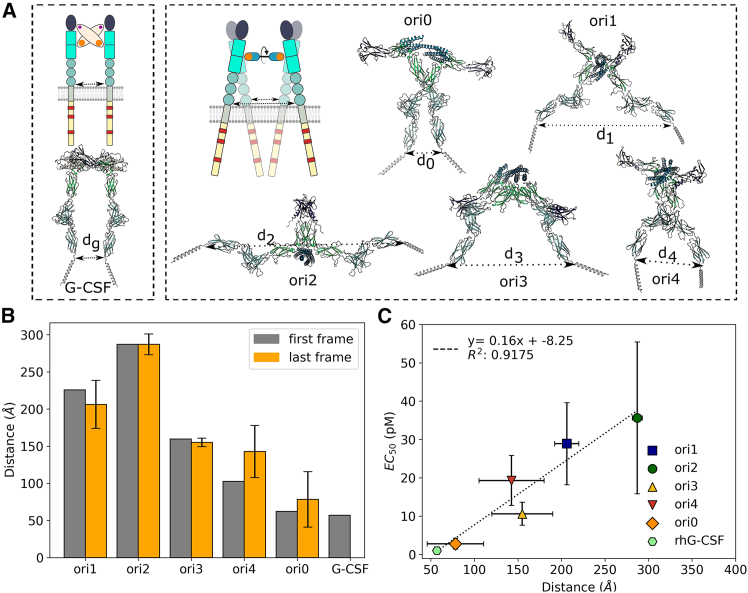
Agonists designed to associate G-CSFR in non-native dimeric geometries yield distinct cell activation potencies (A) Unlike the native 2:2 ligand-receptor assembly induced by G-CSF (PDB: 2D9Q), we developed a range of novel ligands by rigidly connecting two copies of the affinity-enhanced bv6 module (supplemental methods; Figure S12) that form 1:2 ligand-receptor complexes. Modeled complexes of these ori designs (ori0–ori4) and G-CSFR indicate varying transmembrane domain spacings (*d*_*x*_). Ori0 was designed to closely mimic this distance parameter to that of G-CSF (i.e., *d*_*0*_ ≈ *d*_*g*_). (B) Molecular dynamics simulations performed on a selected set of designed ori candidates confirmed the rigidity of the design linkers. Orange bars represent the mean spacing and its standard deviation from five simulation replicas. (C) Proliferation assays in NFS-60 cells of the five selected ori designs and rhG-CSF (Lenograstim) showed the proliferative EC_50_ (mean and standard deviation of at least five independent experiments) to strongly correlate to the modeled transmembrane domain spacing (*d*_*x*_) for the different proteins. The dotted line represents the linear fit between the obtained EC_50_ and the distances of the last frame (where the *d*_*g*_ of G-CSF was obtained as a single value from the crystal structure (PDB: 2D9Q).

Besides affinity, the geometry of receptor dimerization can impact its activation. Hence, we sought to design orientation-rigging (ori) agonists that are composed of two rigidly connected bv6 modules (Figure 1E). We thus modeled helical connectors to introduce different inter-domain rotations (Figures S12A and S12B), and predicted the 2:1 assemblies using AlphaFold2^53^ (Figure 3A). The five templates, named ori0–ori4, underwent sequence design using the combinatorial sampler application of the Damietta software (version 0.36)^47^ and filtering based on their structural rigidity in molecular dynamics simulations (Figures S12C and S12D). Our models predicted spacings across the receptor transmembrane domains (TMD) of *d*_*0*_ = 78 ± 32 Å, *d*_*1*_ = 206 ± 14 Å, *d*_*2*_ = 287 ± 6 Å, *d*_*3*_ = 155 ± 35 Å, and *d*_*4*_ = 143 ± 37 Å, for ori0–ori4, respectively, which compares to *d*_*g*_ = 57 Å for the native G-CSF:G-CSFR complex (Figures 3A and 3B).

The produced ori designs were helical, stable, and monomeric, with the exception of ori1, which showed monomeric-dimer exchange (Figures S13 and S14). SPR titrations showed the ori designs to bind G-CSFR as expected, with affinities ranging from 0.44 to 0.73 nM (Table 1; Figure S15).

### The geometric modulation of the G-CSFR by design agonists differentially affects cell proliferation

Proliferation assays in NFS-60 cells showed the ori design EC_50_ values to range from 36 ± 19.8 pM down to 2.8 ± 1.4 pM (Table 1; Figure S16), where only ori1 displayed a reduced maximum proliferation level (E_max_) compared to rhG-CSF (Figure S16H). The proliferative EC_50_ values of these design agonists correlated well with the modeled distances across the receptor TMD for these designs (*R*^*2*^ = 0.92; Figure 3C). Ori0 was the most potent because it dimerizes G-CSFR with similar TMD spacing to G-CSF (*d*_*0*_ *≈ d*_*g*_). Conversely, the design agonists inducing the farthest TMD spacing, ori1 and ori2, showed the lowest proliferation.

### Design agonists impose distinct complexes with G-CSFR and can override the native ligand

**Figure 4:**
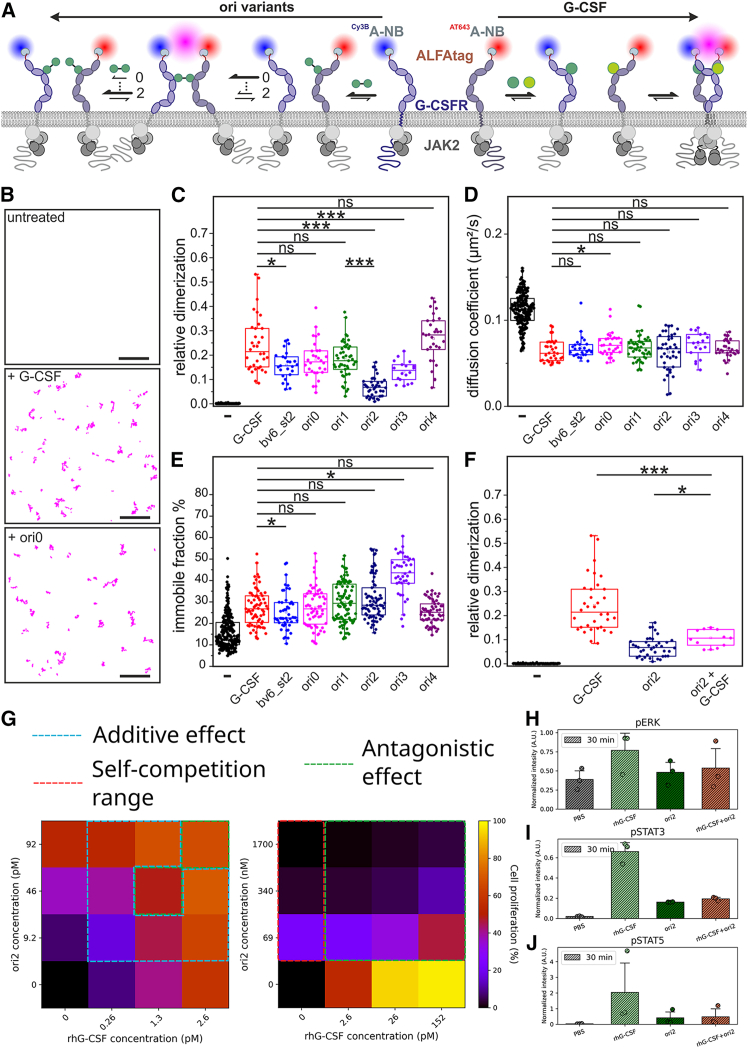
The design agonists differentially dimerize G-CSFR subunits in live cells (A and B) Dual-color single-molecule co-tracking for quantifying G-CSFR dimerization in live cells. (A) G-CSFR labeled via an N-terminal ALFA-tag using a mixture of nanobodies conjugated with Cy3B and AT643, respectively. The G-CSFR monomer-dimer equilibrium probed by dual-color single-molecule co-tracking depends on the 2D and 3D binding affinities of the respective design agonist. Different dimerization principles by G-CSF (arrow to the right) and design agonists (arrow to the left), as well as the consequences of altered orientation on the assembly kinetics are schematically outlined (numbers on arrows indicate expected impact of ori0 (0) or ori2 (2). (B) Typical single-molecule co-trajectories of G-CSFR in the absence of agonist and presence of G-CSF or ori0, respectively. A region of interest from a single cell is shown. Scale bar: 5 μm. (C) Relative dimerization levels of G-CSFR in the absence of ligand and in the presence of rhG-CSF or design agonists (10 nM). (D) Diffusion constants of G-CSFR monomers in the absence of ligand compared to G-CSFR dimers in the presence of rhG-CSF and design agonists identified by co-tracking analysis. (E) Comparison of the immobile fraction of G-CSFR in the absence of ligand and in the presence of rhG-CSF and design agonists. (F) Comparison of G-CSFR dimerization by G-CSFR by rhG-CSF, ori2, and an equimolar mixture of both (10 nM each). (G) Combined treatment of NFS-60 cells with near-EC_50_ concentrations of rhG-CSF and ori2 shows mostly in additive effect on cell proliferation (left), while higher concentrations of ori2 saturate the receptor binding in a monovalent manner and readily outcompete rhG-CSF-induced proliferation, resulting in an antagonistic effect. The heatmaps show the percentage of confluence, and combination treatments where additive, antagonistic, or self-competition effects are outlined in cyan, green, or red, respectively. (H–J) Outcompetition of rhG-CSF signaling was also observed, where combined treatment with rhG-CSF (26 pM) and ori2 (340 nM) leads to the lowering of ERK (H), STAT3 (I), and STAT5 (J) phosphorylation toward ori2-induction levels. Normalization of western blot detection intensity in (H)–(J) was done with respect to total protein stain in the corresponding lane (blots are shown in Figure S20). Box plots in (C)–(F) were obtained from analysis of multiple cells. For (C)–(E), the numbers of cells and experiments, respectively, are 124 and 17, 34 and 3, 25 and 2, 30 and 2, 47 and 3, 39 and 3, 18 and 2, and 30 and 2 for each column, going from left to right. For (F), the numbers of cells and experiments, respectively, are 124 and 17, 34 and 3, 39 and 3, and 10 and 1 for each column, going from left to right, with each dot representing the result from one cell. Box plots indicate data distribution of the second and third quartiles (box), median (line), mean (square), and 1.5× interquartile range (whiskers). Statistics were performed using two-sided two-sample Kolmogorov-Smirnov test (ns, not significant; ∗*p* ≤ 0.05; ∗∗∗*p* ≤ 0.001).

To query the properties of these design:receptor complexes in a cellular environment, we explored G-CSFR assembly at the plasma membrane by live-cell single-molecule fluorescence imaging. To this end, we fused ALFA-tag to the N terminus of G-CSFR, expressed it in HeLa cells, and labeled it with a mixture of anti-ALFA nanobodies (NBs) conjugated to either Cy3B or AT643 (Figure 4A).^54^ We additionally transfected the cells with JAK2 C-terminally fused to mEGFP, as the latter was shown to contribute to receptor assembly.^7^ This construct lacks the tyrosine kinase domain (TK) to eliminate any bias from downstream signaling activation. Simultaneous dual-color single-molecule imaging by total internal reflection fluorescence microscopy enabled identifying individual G-CSFR dimers by co-localization and co-tracking analysis (Figures 4B and S17A).

As expected, G-CSFR dimerization was negligible in the resting state but strongly stimulated by G-CSF and all design agonists (Figure 4C). While similar dimerization levels were observed for most design agonists, dimerization induced by ori2 and ori3 was significantly lower. Importantly, similar receptor cell surface densities were used in all experiments (Figure S17B), thus ensuring that dimerization levels were not biased by the two-dimensional (2D) receptor concentration. Since the three-dimensional (3D) binding affinity of all design agonists is identical because they share the same bv6 binding site, the distinct differences in dimerization potencies of different design agonists can be ascribed to different 2D binding affinities (Figure 4A). This can be rationalized by changes in the 2D association rate constant $k_{a}^{2D}$, but not the complex stability $k_{d}^{2D}$ due to geometrical constraints imposed by the orientation of the receptor ectodomains at the cell surface. With the formation of 1:1 G-CSFR:agonist complexes competing with G-CSFR dimerization, reduction in 2D binding affinity is accompanied by a lower maximum of the bell-shaped concentration-dimerization relationship of homodimeric receptors.^55^ Changes in dimerization efficiency can explain differences in the EC_50_ values, as the overall receptor binding affinity depends on dimerization efficiency,^56^^,^^57^ which was reported for synthetic ligands of EPOR.^20^^,^^21^ However, different G-CSFR dimerization levels induced by different design agonists did not directly correlate with their activity. For instance, ori1 showed substantially higher dimerization levels compared to ori2, yet their NFS-60 proliferation activity was similar. Additionally, ori1 and ori0 had similar dimerization levels, but ori0 had ∼10 times higher proliferation activity compared to ori1 (Table 1). These results therefore confirm that biased signaling observed for our design agonists is related to the altered geometry of the signaling complex rather than weakened dimerization.

Our analysis of G-CSFR complexes indicated that their diffusion coefficients were similar among the different ligands, which were ∼45% lower than unbound G-CSFR monomers (Figure 4D). However, the fraction of immobile G-CSFR increased by a factor of ∼2, with ori3 showing surprisingly higher immobilization by a factor of ∼3 (Figure 4E). Receptor immobilization is very likely caused by endocytosis, and therefore differences across design agonist may indicate altered endocytic uptake.^58^

We then tested whether the ori2, which is predicted to impose the farthest inter-TMD spacing, can override native complex formation in the presence of G-CSF. We indeed observed effective competition of G-CSF binding to receptors at the cell surface by ori2 at equimolar concentrations of 10 nM of each ligand (Figures 4F, S17C, and S17D). To further evaluate ori2 to outcompete G-CSF in cells, we studied the effect of combination treatment with both ligands. First, we performed proliferation assays using near-EC_50_ concentrations of either rhG-CSF, ori2, or a mixture of both (Figures 4G, S18A, and S18B). These experiments showed mostly additive effects, except for two concentrations of ori2, which antagonized rhG-CSF at picomolar levels as estimated by the combination index (CI; Figure S18C). Second, to emphatically demonstrate this antagonizing combination effect, we repeated these proliferation assays at nano- to micromolar concentrations of ori2 against concentrations of G-CSF that correspond to serum concentrations at physiological (2.6 pM^59^^,^^60^), bacterial infection (26 pM^61^^,^^62^), and sepsis (152 pM^63^) conditions, since under the former conditions, partial agonism that maintains granulopoiesis while reducing inflammation is desirable. These results showed that pico- to nanomolar concentrations of ori2 can achieve partial agonism of G-CSFR while completely abolishing G-CSF activity (Figures 4G and S19), while higher ori2 (sub-micromolar) concentrations only partially dimerize the receptor due to self-competition. Finally, we sought to confirm this effect through tracking of secondary messenger phosphorylation, where phospho-STAT (pSTAT)3/5 and phospho-extracellular signal-regulated kinase (pERK)1/2 levels were overridden by ori2 (Figures 4H–4J and S20).

Taken together, these results establish alternate G-CSFR modulation through alternate geometries and demonstrate its usefulness.

### Different design agonists tune G-CSFR signaling amplitudes, kinetics, and cellular response patterns

**Figure 5:**
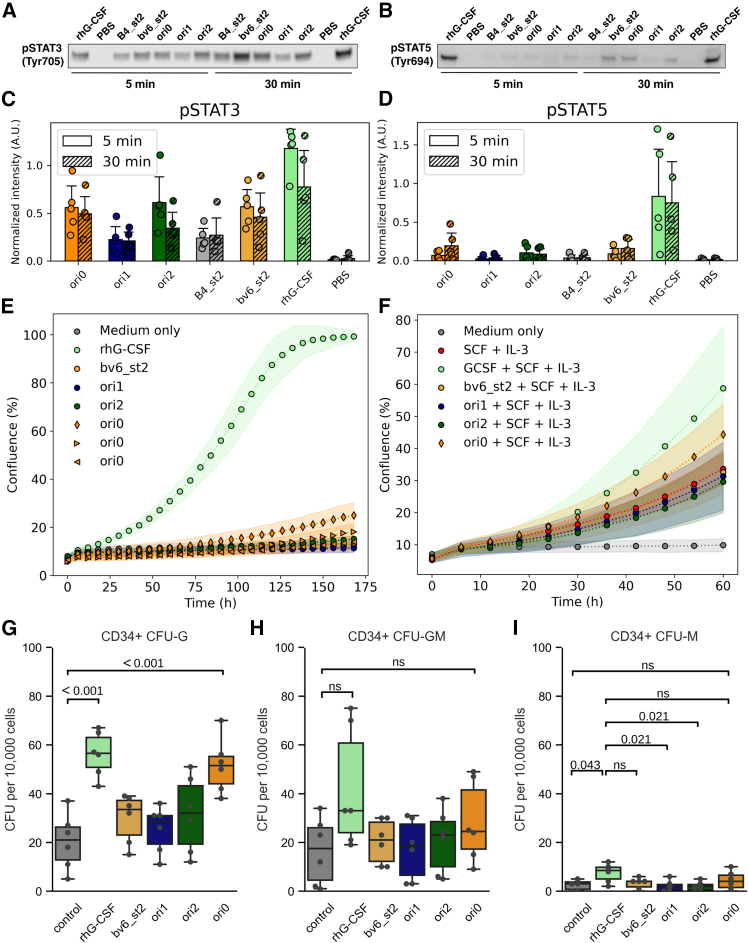
The affinity and geometry of the design agonists bias intracellular signaling and primary stem cell differentiation (A and B) Representative western blot images of intracellular levels of phospho-STAT3 (Tyr705) (A) and phospho-STAT5 (Tyr694) (B) proteins after treatment of NFS-60 cells with 1 nM of different designs (saturating conditions) for 5 min (left) or 30 min (right). Total protein staining was used as a loading control. Five biological replicates of this experiment showed the same trend of higher pSTAT3/pSTAT5 ratio by the design agonists (see Figure S21). (C and D) The pSTAT3 (C) and pSTAT5 (D) levels were quantified after 5 and 30 min from those replicates. Shown is the level of pSTAT3 or pSTAT5 divided by the total protein-normalized STAT3 or STAT5 levels, respectively. (E and F) To probe the effect of the design agonists (100 ng/mL) and rhG-CSF (10 ng/mL) on healthy donors’ CD34^+^ HSPCs, proliferation assays were performed without (E) or with (F) the addition of 50 ng/mL SCF and 20 ng/mL IL-3. Ori0 without SCF and IL-3 were tested at three concentrations: 1 ng/mL (triangle, left), 10 ng/mL (triangle, right), and 100 ng/mL (diamond). Shown are the mean (points) and standard deviation (shades) of three parallel replicates. (G–I) Additionally, we performed CFU assays of healthy donors’ CD34^+^ and progenitors incubated on semi-solid medium supplemented with corresponding cytokines and design agonists. IL-3 and SCF were added to all samples. Shown is the fold change to rhG-CSF of the quantified CFUs of granulocytes (CFU-G) (G), granulocyte-macrophages (CFU-GM) (H), and macrophages (CFU-M) (I). The circles represent the obtained values for each condition of three independent experiments with two parallel replicates each. The indicated *p* values were estimated by an ordinary one-way ANOVA followed by a Tukey HSD test.

Since STAT3 phosphorylation occurs at the membrane-distal regions of the intracellular segments of the receptor, while STAT5 is phosphorylated at the membrane-proximal receptor region,^64^ we reasoned that the relative ratio of pSTAT3/5 might be altered by different designs. Western blot analysis after stimulating NFS-60 cells for 5 and 30 min showed increased pSTAT3 (Tyr705) levels for bv6_st2 as compared to B4_st2, confirming the increased potency by enhancing the affinity (Figures 5A–5C and S17). For the oris, however, striking differences were observed: while after 5 and 30 min ori0 and ori2 reached similar pSTAT3 levels and activation kinetics to bv6_st2, markedly lower pSTAT3 levels were found for ori1 for 5 min of stimulation (Figure S17). Designed proteins showed substantially slower STAT5 phosphorylation (pSTAT5, Tyr694) in comparison to rhG-CSF (Figures 5B and 5D). Again, bv6_st2 and ori0 phosphorylated STAT5 more efficiently than B4_st2, ori2, or ori1, but the magnitude of pSTAT5 levels was markedly weaker for all variants compared to rhG-CSF. These results highlight that amplitudes and kinetics of STAT3 and STAT5 phosphorylation are affected by G-CSFR dimerization geometry.

G-CSF induces proliferation and granulocytic differentiation of HSPCs, and until now it has been impossible to dissect these two cellular outcomes intentionally. Different magnitudes and kinetics of STAT3/STAT5 phosphorylation have different outcomes on HSPC functions, with elevated pSTAT3 mainly inducing myeloid differentiation, whereas elevated pSTAT5 mainly induces cell proliferation.^65^ To assess whether the different intensities and kinetics of STAT3/STAT5 phosphorylation also result in different functional outcomes of the designs on primary human HSPCs compared to rhG-CSF, we evaluated the activity of the designs to induce proliferation and myeloid differentiation of these cells. We incubated primary human cord blood-derived CD34^+^ HSPCs in Stemline II Hematopoietic Stem Cell Expansion medium in the presence of 10 ng/mL rhG-CSF or designed agonists at different concentrations using an IncuCyte S3 Live-Cell Analysis System and evaluated cell proliferation over time. We found that the designed proteins alone induced only weak proliferation compared to rhG-CSF (Figure 5E), which was potentiated when the intracellular signaling activation by the designs was enhanced by the addition of the stem cell-activating cytokines IL-3 and stem cell factor (SCF) (Figure 5F).

In line with previous observations, ori0 had the highest proliferative activity, but it was still weaker than rhG-CSF. We next assessed the activity of designs to induce myeloid differentiation using a colony-forming unit (CFU) assay with primary human cord blood-derived CD34^+^ HSPCs. We found that ori0 was as active as rhG-CSF in inducing granulocytic differentiation of HSPCs when combined with IL-3 and SCF (Figures 5G and 5H). Meanwhile, the other design agonists (ori1, ori2, and bv6_st2) did not significantly induce CFU-Gs in comparison with untreated cells. Interestingly, all design agonists had minimal or no effect on the formation of granulocyte-monocyte (CFU-GM) and monocytic (CFU-M) colonies (Figure 5I), suggesting their selective activation of granulocyte progenitors located at the later stage of the myeloid differentiation hierarchy after the granulocyte-monocyte bifurcation, when cells are already committed to granulocyte differentiation and less proliferative. These results further support the role of G-CSFR association geometry in fine-tuning STAT3/STAT5 phosphorylation and potentially inducing specialized granulocytic differentiation of HSPCs.

### Designed G-CSFR agonists modulate intracellular transcriptional programs with hematopoietic bias

**Figure 6:**
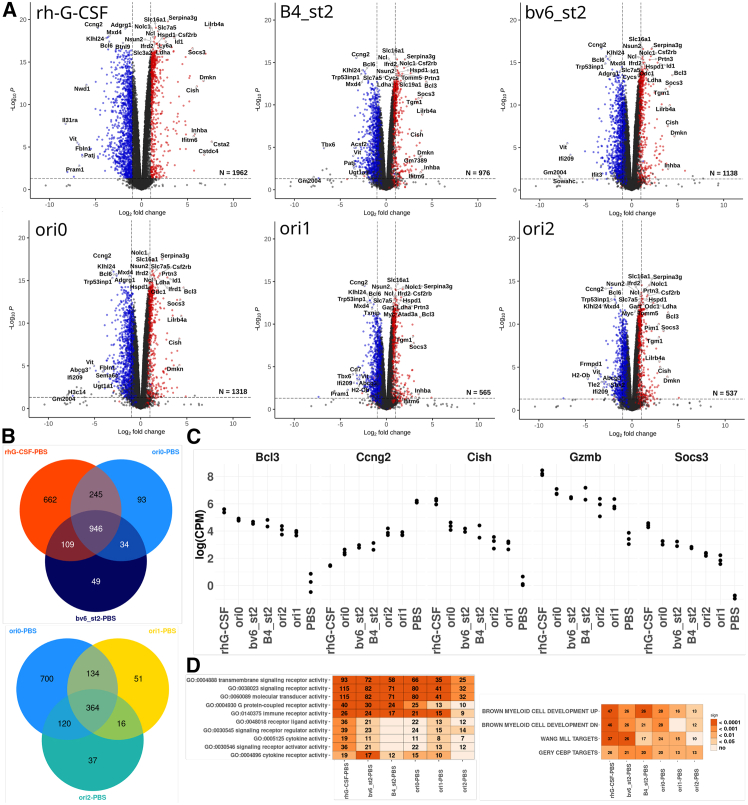
The design agonists regulate G-CSFR downstream signaling to varying degrees (A) The number (*N*) of differentially expressed genes in NFS-60 cells after treatment with rhG-CSF or different designs compared to PBS-treated control. Blue (downregulated genes) and red (upregulated genes) dots in the volcano plots indicate genes that are shared between the corresponding design and rhG-CSF. (B) The Venn diagrams show the numbers of shared and design-specific regulated genes. (C) Examples of the top five differentially expressed hematopoiesis-related genes. (D) Gene Ontology (GO) analysis of significantly regulated signaling pathways in the indicated groups. Each number indicates the number of differentially expressed genes related to the corresponding pathway, and the colors represent the significance score. Extended details on regulated genes and pathways are shown in Figures S22–S24).

To assess the transcriptomic footprints of our design agonists, we carried out RNA sequencing (RNA-seq) of NFS-60 cells undergoing 8-h treatment with 1 nM of design agonists or rhG-CSF. G-CSF regulated expression of 1,950 genes compared to the phosphate-buffered saline (PBS)-treated control group (log fold change [FC] 1, false discovery rate [FDR] < 0.05). The number of differentially expressed genes was lower for all designs and was affected by the inter-TMD spacing (e.g., 1,292 genes for ori0 versus 524 genes for ori1) and binding affinity (e.g., 1,115 genes for bv6_st2 versus 945 genes for B4_st2). Differentially regulated genes overlapped across all design agonists and were largely subsets of the rhG-CSF group (Figures 6A and 6B). The general trend revealed a graded number of regulated genes across the groups, following the order G-CSF > ori0 > bv6_st2 > B4_st2 > ori2 > ori1 > PBS (Figures 6C and S22). Gene set enrichment analysis showed similar enrichment patterns, particularly in the regulation of hematopoiesis/myelopoiesis-related gene sets among rhG-CSF and the design agonists, which followed the same trend. Among them were BROWN MYELOID CELL DEVELOPMENT, WANG MLL TARGETS, SCHRAETS MLL, GERY CEBP TARGETS, signaling receptor activity (Gene Ontology GO:0038023), molecular transducer activity (GO:0060089), and transmembrane signaling receptor activity (GO:b0004888) (Figures 6D and S23).

As with individual genes, the degree of regulation of the enriched gene sets was either similar to the rhG-CSF group or gradually reduced (Figure S24). As an example, almost all genes enriched in the myeloid-specific CEBP_TARGETS signaling were gradually regulated (Figure 6D). No, or almost no, hematopoiesis/myelopoiesis-related signaling pathways were specifically enriched in any of the designs but not in the G-CSF group. Remarkably, rhG-CSF but not design proteins regulated the expression of genes involved in immunomodulation of T lymphocytes, MDSCs, monocytes/macrophages, dendritic cells, and basophils such as IL-18R1, Lilrb4, Lilrb4b, Inhba, Ifitm6, Stfa1, Slfn2, Osm, Ly6i, Il19, Oas1g, Apol9b, Tarm1, Ifit3, Ccr5, Trem1, Acod1, Nlrp12, Mx2, and Il17c. These data argue for the possible specificity of the designs toward hematopoietic cells. Chemokine receptors Cxcr2, Cxcr4, Cx3cr1, and Ccr5 were also regulated by G-CSF but not design proteins. Additionally, VEGFR/Kdr; transcription factors Hoxa1, Hoxb3, Klf3, Junb, Meis3, Egr1, and Hoxc4; cell-cycle regulators Cdkn1a/P21 and Cdkl2; and adhesion molecules Amigo2, Adgre1, Tns1, Trio, and Pcdhga6 were regulated by rhG-CSF only. Simultaneously, a very small set of genes (e.g., Vipr1, Serpinb10, Hus1b, Plcxd1, Rab33a, Trim58, Gimap1) was regulated by design proteins, but not by rhG-CSF, with different degrees of regulation between different designs.

## Discussion

The *de novo* design of cytokine receptor ligands with small, stable structures offers several advantages. This strategy gives room for functional enhancements that typically erode structural stability^66^ and provides building blocks for constructing rigid larger structure to module receptors in novel ways. In this work, we leverage the *de novo*-designed G-CSFR binder (Boskar4)^46^^,^^67^ through affinity and geometry engineering to tune G-CSFR activity by inducing specific signaling patterns and thus distinct cellular activity.

By deriving high-affinity, bivalent binders against the receptor, we could create design agonists with improved receptor dimerization efficiency, which showed increased granulopoietic activity *in vitro* and *in vivo*, in comparison to the parent design. These functionally enhanced agonists remained hyper-thermostable, in contrast to previous studies aiming to improve the stability of rhG-CSF, which only led to marginal improvements in its biophysical properties.^68^^,^^69^^,^^70^ The stability of the design agonists and the diverse binding site sequences reported here are invaluable for de-risking further optimization to cloak any immune epitopes during pre-clinical development. Besides affinity, we sought to explore the role of receptor association geometry, whereby our results highlighted the inter-TMD spacing of G-CSFR subunits as another key parameter of signaling strength. The analysis of the phosphorylation of the main secondary messengers STAT3 and STAT5 revealed that design agonists with higher affinity and shorter inter-TMD spacing selectively increase the intracellular level of pSTAT3 while only minimally inducing pSTAT5. This could be linked to the different locations of STAT3 and STAT5 phosphorylation sites along the intracellular segment of G-CSFR,^64^^,^^71^^,^^72^ where the inter-TMD spacing can affect the balance between these two processes, as pSTAT3 primarily drives differentiation, while pSTAT5 mainly drives proliferation.^71^^,^^73^^,^^74^^,^^75^

Single-molecule fluorescence imaging confirmed the ability of most design agonists to potently dimerize G-CSFR at levels comparable to those of G-CSF. Given the matched binding affinity of all the ori design agonists, the reduced dimerization levels observed for ori2 and ori3 indicate that manipulating the geometry of G-CSFR dimers is accompanied by a loss of 2D association kinetics. Changes in signaling activity and specificity, however, cannot be attributed to changes in dimerization and rather support the idea that design agonists associate G-CSFR dimers with altered geometry. The very similar diffusion constants of all agonist-induced dimers, however, suggest more subtle changes in orientation as compared to the predicted structures, which should significantly reduce mobility. This could be explained by the additional strain imposed by membrane anchoring and additional interactions involving associated JAKs. Nonetheless, the distinct rise in the immobile fraction observed for ori3 points toward changes in endocytic uptake for that particular agonist. This warrants further investigation, as endocytic trafficking of cytokine receptor signaling complexes is emerging as a complex determinant regulating the potency and kinetics of signal activation and the specificity of cellular responses.^58^^,^^76^^,^^77^

Interestingly, single-molecule imaging revealed that ori2 substantially reduced receptor dimerization on the cell membrane, even in the presence of rhG-CSF. This pointed to the capacity of ori2 to impose partial- or sub-agonism in the presence of rhG-CSF. Further analysis of ori2:rhG-CSF combinations highlighted this potential of ori2 to override the proliferative and signaling activity of rhG-CSF under physiolophically and pathologically elevated concentrations of the latter. This capacity of ori2 to induce a basal level of granulopoiesis while antagonizing the effect of elevated levels of endogenous G-CSF can be useful in treating conditions such as inflammation or sepsis, for which targeted G-CSF antagonists are needed.^78^^,^^79^

Further analysis of the design agonists’ effects in primary human HSPCs showed higher-affinity, and shorter-spacing designs led to stronger proliferation, in comparison to other designs, and to more selective formation of granulocytic colonies, in comparison to G-CSF. We infer that these differences might stem from the selective action of the design agonists on more-committed granulocytic progenitors than on the less-differentiated hematopoietic progenitors. The gene-expression patterns indicated differential levels of G-CSFR-triggered transcription activation or inhibition, where the design agonists minimally affect the expression of genes that are not regulated by rhG-CSF. Thus, the design agonists induced only a subset of G-CSFR signaling, without regulating unexpected transcriptional pathways that are not linked to physiological or desired G-CSFR activation. At the same time, the design agonists showed varying degrees of reduced G-CSF-specific transcriptional activity. This was strongest for the gene sets of cytokine regulation and myeloid-specific CEBP targets. Interestingly, only G-CSF regulated gene signatures specific for immune cell regulation, chemotactic activity, or angiogenesis. This corresponds to our initial aims of designing G-CSFR ligands with selective activity on myeloid progenitor cells to induce granulopoiesis or on hematopoietic stem cells to trigger their mobilization from the bone marrow into peripheral blood. The more specific signaling by the design agonists can thus potentially offer a safer means of treating indications such as chemotherapy-induced or inherited neutropenia^31^ or stem cell mobilization.

In conclusion, our findings demonstrate the potential for tailoring G-CSFR agonists to achieve more specific therapies for hematopoietic stem cell disorders and overcome the functional pleiotropy of rhG-CSF. To date, only *de novo*-designed IL-2 and IL-4 mimics, which bind a subset of the natural cytokine’s heteromeric receptor subunits, could achieve more selective biological responses.^80^^,^^81^ Alternatively, tuning of homodimeric receptors could be achieved through engineered agonists with varying affinities and geometries for EPOR^20^^,^^21^ and thrombopoietin receptor.^82^^,^^83^ Our results are the first to establish the tunable modulation potential of G-CSFR. They also warrant the further development of potent, hyperstable, more selective, and tunable sub-agonists of G-CSFR.

## Materials and methods

### Computationally guided library generation for affinity maturation

Two libraries of mutants of six amino acid positions on the Boskar4 receptor-binding sites were generated using either site-saturation mutagenesis or computational design. The mutated positions on the Boskar4 surface were chosen based on two criteria (Figure S1A). The positions in Boskar4 were determined based on which had the smallest average distance to G-CSFR in a modeled complex between the Boskar4 ensemble (PDB: 7NY0^46^) and G-CSFR (PBD: 2D9Q^29^). Residues known to be critical for G-CSF activity were excluded from the final choice.^84^ For the saturation library, high diversity degenerated codons were chosen that contained the original amino acid and other random amino acids, constructing a library of a theoretical diversity (number of distinct gene variants) of 4.0 × 10^6^ and an amino acid diversity (number of distinct protein variants) of 3.2 × 10^6^. Finally, the library was generated by PCR (Tables S2 and S3) using one pair of primers (primers 3 and 4), each containing 3 degenerated sites to linearize an *E. coli* display system plasmid^48^ encoding a fusion between the N-terminal part of intimin and Boskar4 (pNB4, Table S4; Figures S1B and S1C). The obtained PCR product was purified with the Wizard SV Gel and PCR Clean-Up System (catalog no. A928, Promega) and blunt-end ligation was performed followed by electroporation of freshly made competent cells^85^ to yield 3.6 × 10^7^ total transformants. The *E. coli* strain DH10B T1^R^ (catalog no. C640003, Invitrogen) was used for all display experiments.

In more detail, prior to transformation, 0.4 U/μL T4 polynucleotide kinase (EK0031, Thermo Scientific) was incubated with 5 ng/μL linearized PCR product for 30 min at 37°C, according to the manufacturer’s instructions. After that, 0.25 U/μL T4 DNA ligase (catalog no. EL0011, Thermo Scientific) was added and incubated overnight at 16°C. The ligation product was purified with the same PCR Clean-Up System mentioned above and eluted into 20 μL filtered double-distilled H_2_O. The complete 20 μL were transformed into electrocompetent *E. coli* as described by Tu et al. with the following changes.^85^ In short, 50 mL freshly grown *E. coli* exhibiting an optical density at 600 nm (OD_600_) of ∼0.6 were washed two times with 35 mL filtered H_2_O. After washing, the bacterial pellet was resuspended with the purified ligation mix. The electroporation was performed at 1,250 V and 5 ms. The cells were grown in 1 mL SOC Outgrowth Medium (catalog no. B9020, NEB) for 2 h in a standard glass tube at 30°C with 160 rpm. A 10-fold dilution series of the cells was made in SOC medium, and they were plated on agar plates containing 34 μg/mL chloramphenicol and 2% (w/v) d-glucose to estimate the total number of transformants. The rest of the sample was plated on five agar plates of the same type and incubated at 30°C for ∼20 h. The bacterial lawn was scraped from the plates and well mixed in 10 mL lysogeny broth (LB) medium. From 500 μL of bacterial suspension, the display plasmid containing the Boskar4 library was isolated and analyzed by Sanger sequencing with primers 19 and 20.

For the designed library, the Damietta sp application^47^^,^^86^ was used to estimate the free energy change of each single mutant of the same position and same amino acids per position as the control library in a non-combinatorial manner. For each of the six positions, five amino acids with the lowest free energy change plus the original amino acid of Boskar4 at the corresponding position were used to define the designed, size-reduced library with a theoretical diversity of 3.89 × 10^4^. The library was generated by PCR in the same way as the original but using three pairs of primers (primers 5–10, Table S2), such that the final library contained 15.6% of the desired amino acids. The primer composition was designed by using the online tool of SwiftLib.^87^ The designed library had a final theoretical diversity of 2.5 × 10^5^, an amino acid diversity of 2.3 × 10^5^, and 2.7 × 10^7^ total transformants were yielded.

### Screening procedure for enhanced binders

The FACS-based screening of bacterially displayed Boskar4 variants followed a slightly adapted procedure from that described by Salema et al*.*^48^ In brief, G-CSFR was first fluorescently labeled. This was done by incubating 100 μg rhG-CSFR (catalog no. 381-GR/CF, R&D Systems) in 950 μL PBS for 2 h at room temperature (RT) with Biotin-NHS (catalog no. H1759, Sigma) in a 1:20 molar ratio (G-CSFR:Biotin-NHS). The reaction was stopped with 50 μL Tris (1 M pH 7.5). After 1 h of incubation on ice, the product was purified with a desalting column (Sephadex G25 PD-10, GE Healthcare), and the elution fractions were concentrated with Amicon Ultra centrifugal filter units (3 kDa; catalog no. UFC800324, Merck) to a concentration of 0.24 mg/mL (3.6 μM). Aliquots of the biotinylated protein (hereafter referred to as BioGCSFR) were stored at −20°C until use.

For the first sorting, DH10B T1^R^ *E. coli* cells were transformed with the display-vector pN containing a Boskar4 library. The bacteria were freshly grown on LB agar plates containing 34 μg/mL chloramphenicol and 2% (w/v) d-glucose at 30°C yielding ∼3.0 × 10^8^ total transformants. Following the harvesting of bacteria from the agar plates, 1 mL resuspended bacterial culture exhibiting an OD_600_ of 3.3 was subjected to two successive washes, each involving 1 mL LB medium. Subsequently, the culture was diluted to an initial OD_600_ of 0.33 by adding it to 10 mL LB medium containing 34 μg/mL chloramphenicol. The cells were cultivated at a temperature of 30°C and an agitation rate of 160 rpm. After a duration of 1 h, 50 μM isopropyl β-d-1-thiogalactopyranoside (IPTG) was introduced into the culture, and the cultivation was continued for an additional 3 h under the same growth conditions. Cells equivalent to 1 mL of a cell resuspension possessing an OD_600_ of 1 were collected, subjected to 2 washes with 1,000 μL of PBS, and eventually resuspended in 400 μL PBS. We incubated 190 μL of this cell suspension with 10 nM BioGCSFR for 1 h at RT, followed by 1 wash with 1,000 μL PBS. The washed cells were resuspended in 200 μL PBS containing 0.375 μL phycoerythrin (PE)-streptavidin (catalog no. 405203, BioLegend) and were subsequently incubated for 30 min at 4°C. Finally, the sample was washed one more time with 1,000 μL PBS and resuspended in 1,000 μL PBS. FACS was performed with a BD FACSMelody Cell Sorter having set the photomultiplier tube (PMT) voltage for PE to 517 V, side scatter (SSC) to 426 V, and an SSC threshold to 673 V. No gating was applied. All flow cytometry results were analyzed using FlowJo version 10.1 software (BD Life Sciences). We mixed 300 μL of the sample with 4 mL ice-cold PBS, and the flow rate was adjusted to result in an event rate of ∼8,000 events per second; 100,000 events were recorded per sample. The top ∼0.1% of the total population depending on an SSC-A/PE-A plot were sorted into a 1.5-mL tube provided with 200 μL LB medium and cooled to 5°C. The sorting was continued until at least 20-fold more processed events were screened than the theoretical diversity of the correspondent library was. All sorted cells were plated on appropriate LB agar plates and incubated for ∼20 h at 30°C. The plates were harvested and grown overnight in LB medium containing 34 μg/mL chloramphenicol and 2% (w/v) d-glucose at 30°C under static conditions. From this culture, 1 mL resuspended bacteria with an OD_600_ of 3.3 used for the next selection cycle performed in the same way as the first.

This was followed by evaluating the relative binding of the top clones using plate-based assays. At this stage, single colonies of bacteria pools that were enriched for 3 or 5 cycles of FACS-based sorting (as described in the section above) were grown overnight in 100 μL LB (supplemented with 34 μg/mL chloramphenicol and 50 μM IPTG) in a 96-well plate sealed with Breathe-Easy sealing membrane (Z380059, Sigma-Aldrich) at 30°C with 1,100 rpm on a table-top shaker (Thermomixer comfort, Eppendorf) covered with aluminum foil. The next day, the cells were centrifuged at 3,200 × *g* for 5 min, the supernatant was decanted, and the cells were resuspended in 150 μL PBS. The washing was repeated the same way one more time, and the cells were resuspended in 50 μL PBS containing 10 nM BioGCSFR. After the plate was incubated for 1 h at RT, the cells were washed once with 200 μL PBS and resuspended in 50 μL PBS. To visualize the binding activity of each displayed Boskar4 variant to BioGCSFR, two separate secondary stainings, one fluorescent and one chemiluminescent, were performed as described in the following paragraphs.

For the fluorescent staining, 25 μL of the cells were mixed with 25 μL PBS containing 0.094 μL PE-streptavidin followed by a 30-min incubation at 4°C in the dark. After washing the cells one more time with PBS, the OD_600_ and the fluorescence (excitation wavelength 495 nm, emission wavelength 574 nm) were measured on a plate reader (Synergy H4 Hybrid Microplate Reader, BioTek).

For the chemiluminescence stain, 25 μL of the cells were mixed with 25 μL PBS containing 1.25 μg/mL Avidin-horseradish peroxidase (HRP) conjugate (Invitrogen, catalog no. 434423) followed by a 30-min incubation at 4°C in the dark. Afterward, the cells were washed once with 200 μL PBS and resuspended in 100 μL PBS. The OD_600_ of each well was measured with the plate reader. In a separate dark plate (Greiner Bio One, catalog no. 655097) 12.5 μL cell suspension was added to 75 μL PBS. Then, 12.5 μL enhanced chemiluminescence (ECL)-substrate-solution (Bio-Rad, catalog no. 1705061,) was pipetted quickly into each well, and the plate was sealed with parafilm and mixed vigorously for 10 s on a vortex orbital shaker. Then, the luminescence of each well was measured on the plate reader (Synergy H4 Hybrid Microplate Reader, Agilent BioTek) set to an integration time of 1 s, a gain of 135, normal read speed and a 100-ms delay, and a read height of 1 mm.

Both readouts were analyzed by normalizing to the corresponding cell density and calculating a *Z* score for each sample over all wells of the same condition. From the top 5 variants of each condition, plasmids were isolated with a plasmid preparation kit (Macherey-Nagel, catalog no. 740588,) from a 3-mL overnight culture grown at 30°C, with 160 rpm in LB with 34 μg/mL chloramphenicol and 2% (w/v) d-glucose.

Finally, to obtain the expression-normalized binding of the top clones with unique sequences, another FACS experiment was carried out. The individual bacteria clones carrying the display vector pN with intimin fused either to Boskar4 or enhanced Boskar4 variant (bv1–bv16) were grown overnight at 30°C with 160 rpm in 3 mL LB with the addition of 34 μg/mL chloramphenicol and 50 μM IPTG. The cells were pelleted at 4,000 × *g* for 3 min, the supernatant was discarded and the sample was resuspended in 1,000 μL filtered PBS. This washing step was repeated one more time, and the cells were resuspended in 1,000 μL PBS. We incubated 45 μL cell suspension for 90 min at RT in a total volume of 55 μL with 10 nM BioGCSFR and 1:200 Myc-Tag (9B11) mouse monoclonal antibody (Cell Signaling Technology, catalog no. 2276). After 1 wash with 500 μL PBS, the cells were resuspended in 200 μL PBS containing 0.375 μL PE-streptavidin (BioLegend, catalog no. 405203) and 0.5 μL anti-mouse-immunoglobulin G1 (IgG1)conjugated to Alexa Fluor 488 (Invitrogen A-21202,) and incubated for 30 min at 4°C in the dark. Finally, the sample was washed one more time with 500 μL PBS and resuspended in 1,000 μL PBS; 100,000 events were measured by FACS.

### Protein expression and purification

First, a set of affinity-enhanced Boskar4 variant genes (bv1, bv2, bv6, bv8, bv15, and bv16), which were identified from the affinity maturation screens, were used to generate short tandem fusions (st2 [compare Figure S4]). In more detail, a single Boskar4 variant gene was amplified by PCR from pN separately using two different primer pairs (15 and 16 or 17 and 18; Table S2). The two obtained PCR fragments were assembled by NEBuilder HiFi DNA Assembly Master Mix (New England Biolabs, catalog no. E2621) into a pET28a backbone generated by PCR with primers 13 and 14. Additionally, the monomer of bv6 was cloned from pN to pET28a.

*E. coli* BL21(DE3) carrying a certain expression construct was grown to an OD_600_ of ∼0.6 in LB medium containing 40 μg/mL kanamycin at 37°C and 160 rpm. Cells were induced with 0.5 mM IPTG and the protein expression was performed at 25°C and 160 rpm for ∼16 h. The cells were harvested at 8,000 × *g* for 30 min, the supernatant was decanted, and the pellet was lysed by sonication in lysis buffer (50 mM Tris pH 8, 100 mM [for st2 designs] or 1 M NaCl [for ori designs], 20 μg/mL DNase [ITW Reagents, catalog no. A3778], and protease inhibitor cocktail [Roche, catalog no. 04693132001]). The lysate was centrifuged at 16,000 × *g* for 50 min, and the supernatant was used to perform nickel immobilized metal affinity chromatography. Finally, SEC was performed with PBS on the concentrated sample with a HiLoad 16/600, Superdex 200 pg (Merck, catalog no. GE28-9893-35) column as indicated in the corresponding figure legend description. Fractions that were supposed to contain the protein of the expected size were concentrated by ultrafiltration (10 kDa, Millipore, catalog no. UFC9010), and aliquots were frozen at −20°C until use.

### Protein folding and thermal stability

CD spectra were recorded using a JASCO J-810 spectrometer with 0.3-mL samples at a protein concentration of 0.1 mg/mL in PBS buffer (pH 7.1) placed in 2-mm path length cuvettes. The spectral scans of the mean residual ellipticity of three accumulations were measured at a resolution of at least 0.5 nm over a range of 250–190 nm. NanoDSF was conducted on a Prometheus NT.48 and standard Prometheus capillaries (Nanotemper, catalog no. PR-C002). A temperature ramp of 1°C/min from 20°C to 110°C to 20°C was applied for melting and cooling, using 1 mg/mL protein samples in PBS (unless another buffer is specified in the corresponding figure legend).

### Affinity determination with SPR

To determine the receptor-binding affinity of Boskar4 variants, multi-cycle kinetics experiments were performed on a Biacore X100 system (GE Healthcare Life Sciences). Recombinant human G-CSFR (R&D Systems, catalog no. 381-GR/CF) was diluted to 50 μg/mL in 10 mM acetate buffer at pH 5.0 and immobilized on the surface of a CM5 sensor chip (GE Healthcare, catalog no. 29149604) using amine coupling chemistry. The protein samples were diluted in a running buffer (PBS with 0.05% v/v Tween 20). The measurements were performed at 25°C at a flow rate of 30 μL/min. Four sequential concentrations of the sample solution were used as follows: for Boskar4—5,000, 2,500, 625, and 156.3 nM; for bv6—125, 63.5, 31.3, and 15.6 nM; for all st2 variants (bv1_st2–bv16_st2) and oris (ori0–ori4)—20, 10, 5, and 2.5 nM). These samples were injected over the functionalized sensor chip surface for 180 s, followed by a 600-s dissociation phase with running buffer. At the end of each run, the sensor surface was regenerated with a 60-s injection of 50 mM NaOH. The reference responses and zero-concentration sensograms were subtracted from each dataset (double-referencing). Association rate constants (k_a_), dissociation rate constants (k_d_), and apparent equilibrium dissociation constants (K_D_) were obtained using Biacore X100 Evaluation software following a 1:1 binding kinetic.

### Generation of ori designs

To generate preliminary constructs of the ori designs containing a rigid helix-linker fusing two binding protein monomers, two bv6 monomers were connected N- to C-terminally (helix 4 of monomer 1 to helix 1 of monomer 2) with variable lengths of polyalanine stretches. The obtained sequences were modeled by AF2, and four constructs (ori1, ori2, ori3, and ori4) with an increasing number of connecting alanines were settled as a starting set of designs. The obtained AF2 structures were used as input to optimize the sequence of the helix-linker region with Damietta. Depending on the construct, up to 14 positions (supplemental methods) were mutated utilizing the few-to-many-to-few combinatorial sampler of Damietta (version 0.32^47^). The designs’ structural stability was analyzed using a tempering molecular dynamics routine, and candidates were ranked by their conformational homogeneity score, as previously described by Skokowa et al.^46^ This molecular dynamics analysis was performed for all unique variants that were generated by Damietta, and the most stable variant of each construct was considered to be the final design. To generate a binding protein in the same fashion that minimizes the distance of the two TMDs of the bound G-CSFRs, hypothetical complexes of rigid tandem fusions with varying polyalanine-helix stretches and two bound G-CSFRs were created manually. Based on the expected distance, possible steric hindrances, and overall length of the rigid helix-linker, ori0 was selected and a final design was obtained as described above.

The ori expression constructs were generated the same way as explained for the short tandem fusions (as described in the “Protein expression and purification” subsection). In more detail, the two fragments (fr1 and fr2) encoding each one copy of the bv6 monomer and the corresponding rigid helix-linker were amplified and assembled together with the pET28a backbone. The first fragment was generated with primer 15 plus the corresponding primer for each construct (primers 21, 23, 25, 27, or 29) and the second fragment with primer 18 plus the corresponding primer (primers 22, 24, 26, 28, or 30, Table S2).

### NFS-60 cell proliferation assays

The biological activity of various designs in comparison to rhG-CSF was assayed using the murine NFS-60 cell line, which is commonly used for quantifying rhG-CSF activity.^88^^,^^89^ NFS-60 cells^50^^,^^51^ were maintained at 5% CO_2_ and 37°C in NFS-60 medium (RPMI 1640 medium with additional 1 mM l-glutamine, 1 mM Na-pyruvate, 10% fetal calf serum [FCS], 1% antibiotic-antimycotic [Gibco, catalog no. 15240062], and 12.5% KMG-2: 5637-CM [Conditioned Medium from CLS Cell Lines Service]). Prior to the proliferation assay, the cells were washed 3 times with NFS-60 medium without KMG-2. The assay was performed in black 96-well plates (PerkinElmer, catalog no. 6005660). For this purpose, 45,000 cells/well in a total volume of 150 μL NFS-60 medium without KMG-2 were cultured under maintenance conditions with different concentrations of designs or rhG-CSF for 48 h. Then, 30 μL CellTiter-Blue Reagent (Promega, catalog no. G808) was added to each well, and the cells were further cultivated for approximately 90 min under maintenance conditions. Subsequently, the fluorescence of each well was recorded with a plate reader (Synergy H4 Hybrid Microplate Reader, Agilent BioTek) with an excitation wavelength of 560 nm and emission at 590 nm. The EC_50_ of each design or rhG-CSF was determined by fitting the obtained fluorescence values to their corresponding concentrations using a four-parameter sigmoidal function and the Nelder-Mead Simplex algorithm from the Python module SciPy.^90^ For the analysis of the maximum response of proliferative activity (E_max_), the mean of the three highest concentrations was estimated for each sample, such that all values considered for the analysis were in the activity plateau. These means were normalized to the maximum response within each experiment to account for the general variability of cell activity between the independent replicates. For statistical analysis, an ordinary one-way ANOVA followed by a Tukey honestly significant difference (HSD) test was performed.

### Testing of granulopoietic activity of designed proteins *in vivo*

To test the effect of the designs on zebrafish granulopoiesis, equal volumes (4 nL) of Moevan_control (4 mg/mL), rhG-CSF (2 mg/mL), and Boskar4 variants bv6_st2 (2 mg/mL) and bv8_st2 (2 mg/mL) were injected into the cardinal vein of transgenic *Tg(mpx:GFP)*^52^ larvae at 1.5 days post-fertilization (dpf). Injected larvae were incubated at 28°C. To quantify neutrophils 24 h upon injection, larvae were positioned and oriented laterally within cavities formed in 1% agarose on a 96-well plate and then imaged using an SMZ18 Nikon fluorescence stereomicroscope. The number of GFP-expressing neutrophils was automatically determined by Imaris software using the spot detection tool. FC of the neutrophils was calculated by normalizing to the average of uninjected mpx:*gfp* counterparts at the same developmental stage. Zebrafish lines were maintained according to standard protocols and handled in accordance with European Union animal protection directive 2010/63/EU and approved by the local government (Tierschutzgesetz §11, Abs. 1, Nr. 1, husbandry permit 35/9185.46/Uni TÜ). All experiments described in the present study were conducted on larvae younger than 5 dpf.

For the mouse treatment experiments, B6.SJL-PtprcaPepcb/BoyCrl (Ly5.1) mice, aged between 6 and 8 weeks, were treated with i.p. injections of rhG-CSF, or Boskar4 variants bv6_st2 and bv8_st2. A concentration of 300 μg/kg was used for each protein, injecting mice every second day for a total of five injections. Bone marrow cells of treated mice were isolated by flushing with a 22G syringe and subsequent filtering through a 45-μm cell strainer. Red blood cells were lysed from mouse bone marrow, and the hematological profiles of the isolated cells were acquired using Analyzer Element HT5 (Heska). The absolute cell counts of neutrophil and monocyte populations were recorded. Mice were maintained under pathogen-free conditions in the research animal facility of the University of Tübingen, according to German federal and state regulations (Regierungspräsidium Tübingen, M 05-20 G).

### Single-molecule imaging and analysis

For cell surface labeling, G-CSFR was N-terminally fused to the ALFA-tag. Additionally, to assess the co-expression of JAK2ΔTK it was C-terminally fused to murine (m)EGFP. ALFAtag-G-CSFR was encoded on a pSems vector, including the signal sequence of Igκ (pSems-leader) and JAK2ΔTK-mEGFP on a pSems vector with its native signal sequence.^91^ HeLa cells (ACC 57, DSMZ Germany) were cultured as previously described.^14^ For transient transfection, cells were incubated for 4–6 h with a mixture of 150 mM NaCl, 10 μL 1 mg/mL polyethylenimine (PEI MAX, Polysciences 24765), and 1,500 ng (ALFA-tag-G-CSFR) and 2,500 ng (JAK2ΔTK-mEGFP) of the desired constructs. Labeling, washing, and subsequent imaging were performed the day after transfection and after mounting the coverslips into custom-made incubation chambers with a volume of 1 mL. Cells were equilibrated in medium with fetal bovine serum (FBS) but lacking phenol red supplemented with an oxygen scavenger and a redox-active photoprotectant (0.5 mg/mL glucose oxidase [Sigma-Aldrich], 0.04 mg/mL catalase [Roche], 5% w/v glucose, 1 μM ascorbic acid, and 1 μM methylviologene) to minimize photobleaching.^92^

Selective cell surface receptor labeling was achieved by using anti-ALFA-tag NBs, which were site-specifically labeled by maleimide chemistry via a single cysteine residue at their C termini.^92^ NBs labeled with Cy3B (degree of labeling [DOL]: 1.0) and ATTO 643 (DOL: 1.0) were added at concentrations of 3 nM each at least 10 min before imaging. Coverslips were precoated with poly-l-lysine-graft-poly(ethylene glycol) to minimize unspecific binding of NBs and functionalized with RGD peptide for efficient cell adhesion.^93^

Single-molecule imaging was carried out by dual-color total internal reflection fluorescence microscopy using an inverted microscope (IX71, Olympus) equipped with a spectral image splitter (DualView, Optical Insight) and a back-illuminated electron multiplying CCD camera (iXon DU897D, Andor Technology). Fluorophores were excited by simultaneous illumination with a 561-nm laser (CrystaLaser; ∼32 W cm^−2^) and a 642-nm laser (Omicron: ∼22 W cm^−2^). Image stacks of 150 frames were recorded for each cell at a time resolution of 32 ms per frame, with at least 10 cells recorded in each experiment. Ligands were incubated for 10 min before imaging. All imaging experiments were carried out at RT. Prior to image acquisition, the presence of JAK2ΔTK-mEGFP was confirmed through emission with a 488-nm laser (Sapphire LP, Coherent).

Dual-color time-lapse images were evaluated using in-house developed MATLAB software (SLIMfast4C, https://zenodo.org/record/5712332) as previously described in detail.^92^ After channel registration based on calibration with fiducial markers, molecules were localized using the multi-target tracking algorithm.^94^ Immobile emitters were filtered out by spatiotemporal cluster analysis.^95^ For co-tracking, frame-by-frame co-localization within a cutoff radius of 100 nm was applied, followed by tracking of co-localized emitters using the utrack algorithm.^96^ Molecules co-diffusing for 10 frames or more were identified as co-localized. Relative levels of co-localization were determined based on the fraction of co-localized particles.^93^ Diffusion properties were determined from pooled single trajectories using mean squared displacement analysis for all trajectories with a lifetime greater than 10 frames. Diffusion constants were determined from the mean squared displacement by linear regression.

### Drug interaction analysis between rhG-CSF and ori2

Individual and combined treatments of the NFS-60 cell line using rhG-CSF and ori2 was performed as described for endpoint proliferation assays in the preceding materials and methods sections, with the exception that the cell growth maintenance medium contained 5 ng/mL mouse IL-3 (MedChemExpress, catalog no. HY-P70685) instead of 12.5% KMG-2: 5637-CM (conditioned medium from CLS Cell Lines Service). All combination treatments using rhG-CSF (drug A) and ori2 (drug B), both around the EC_50_ and at high concentrations, were performed under the same experimental conditions. The proliferation activity percentage E was calculated based on the highest ($E_{\max}$) and lowest ($E_{\min}$) fluorescence readouts within each independent experiment whereby:

(Equation 1) $$E=\frac{x-E_{\min}}{E_{\max}-E_{\min}}$$

The measured proliferation activities of the separate treatments were fitted to their corresponding concentrations using a four-parameter sigmoidal function and the Nelder-Mead Simplex algorithm as implemented in the SciPy library.^90^ The function of these fits (E(a) and E(b)) were used to calculate the expected concentrations $A_{x}$ and $B_{x}$ for the CI,^97^^,^^98^ as follows:

(Equation 2) $$CI=\frac{a}{A_{x}}+\frac{b}{B_{x}}$$

where $A_{x}$ and $B_{x}$ are the expected concentrations of individual drugs that yield the same proliferation activity as observed for the combination treatment $E_{a+b}$, while a and b are the applied concentrations for combination treatment.

### Analysis of STAT3/5 and ERK1/2 phosphorylation

To quantify the phosphorylation of key secondary messengers downstream of G-CSFR signaling in NFS-60 cells, we used western blot analysis. Initially, the cells were washed two times with NFS-60 medium (RPMI 1640 medium with additional 1 mM l-glutamine, 1 mM Na-pyruvate, 10% FCS, 1% antibiotic-antimycotic (Gibco, catalog no. 15240062)) and starved at 300,000 cells/mL for 16 h at 5% CO_2_ and 37°C. Subsequently, the starved cells were treated with different G-CSFR agonists. To evaluate the effect of different design agonists, treatment with either PBS buffer or 1 nM of one of the following agonists: rhG-CSF, B4_st2, bv6_st2, ori0, ori1, or ori2 was performed for 5 or 30 min. Alternatively, for G-CSF:ori2 interaction studies, the cells were treated with PBS buffer, 26 pM rhG-CSF, 340 nM ori2, or a combination of the latter two for 30 min.

Following the treatment, the cells were maintained at low temperatures on ice. They were harvested using a refrigerated centrifuge operating at 4°C at 300 × *g* for 2 min. The cells were washed once with ice-cold PBS, and the dry pellets were frozen at −70°C until further use. Whole-cell lysates were obtained by lysing 1 × 10^6^ cells in 200 μL Laemmli buffer (30% glycerol, 6% SDS, 7.5% β-mercaptoethanol, and 0.75% bromophenol blue in 200 nM Tris-HCl pH 6.8), followed by heating at 95°C for 5 min and spinning down.

Proteins were separated by SDS-PAGE and transferred to polyvinylidene fluoride membranes (Invitrogen). Membranes were stained with total protein stain kit (LI-COR Biosciences, catalog no. 926-11010) and imaged according to the manufacturer’s protocol using the Sapphire (Azure Biosystems) imager. After imaging, membranes were blocked with 5% non-fat dry milk-TBS-T (10 mM Tris-HCl pH 8.0, 150 mM NaCl, 0.1% Tween 20) for 1 h at RT, washed 3 times for 5 min with TBS-T buffer, and subsequently incubated with primary antibodies in 8 mL 5% BSA-TBST buffer overnight at 4°C or for 2 h at RT. After washing 4 times for 5 min with TBS-T, membranes were incubated with secondary antibodies in 8 mL milk-TBS-T buffer for 1 h at RT and then washed 4 times for 5 min with TBS-T. The following antibodies and corresponding dilutions were used: primary rabbit antibodies against STAT5 1:1,000 (Cell Signaling Technology, catalog no. 94205S), phosphorylated STAT5 (Tyr694) 1:750 (Cell Signaling Technology, catalog no. 9351S), STAT3 1:1,000 (Cell Signaling Technology, catalog no. 12640S), phosphorylated STAT3 (Tyr705) 1:1,000 (Cell Signaling Technology, catalog no. 9145L), ERK1/2 1:1,000 (Cell Signaling Technology, catalog no. 9102S), and phosphorylated ERK1/2 (Thr202/Tyr204) 1:1,000 (Cell Signaling Technology, catalog no. 9101S); secondary HRP-conjugated anti-rabbit antibody 1:3,000 (Jackson ImmunoResearch, catalog no. 111-035-003). A molecular weight marker (PageRuler Prestained Protein Ladder, Thermo Fisher Scientific, catalog no. 26619) was used to confirm the detection of target proteins.

The protein bands were detected using Clarity (Bio-Rad) or Radiance Plus (Azure Biosystems) ECL western blotting substrate kits and imaged using the Sapphire (Azure Biosystems) imager. Membrane images were analyzed using Image Studio Light 5.2 software (LI-COR Biosciences). Signal intensities from target proteins were normalized by total protein staining.

### Evaluation of time-dependent effects of the designed agonists on the proliferation of CD34^+^ HSPCs

Cells were incubated in poly-l-lysine-coated 96-well plates (2 × 10^4^ cells/well) in Stemline II Hematopoietic Stem Cell Expansion medium (Sigma-Aldrich, catalog no. 50192) supplemented with 10% FBS, 1% penicillin/streptomycin, 1% l-glutamine and 50 ng/mL SCF, 20 ng/mL IL-3, and 10 ng/mL rhG-CSF, or designed agonists at different concentrations using an IncuCyte S3 Live-Cell Analysis System (Essen Bio) with a 10× objective at 37°C and 5% CO_2_. Cell proliferation over time was analyzed using IncuCyte S3 software. Experiments in this study involving human samples were conducted according to the Declaration of Helsinki, and study approval was obtained from the ethical review board of the Medical Faculty, University of Tübingen.

### CFU assay with human HSPCs

Human cord blood CD34^+^ cells were isolated from the mononuclear cell fraction by Ficoll density gradient centrifugation with subsequent magnetic bead separation using the Human CD34 Progenitor Cell Isolation Kit (Miltenyi Biotech Germany, catalog no. 130-046-703). We plated 1 × 10^4^ cells/mL CD34^+^ cells in 35-mm cell culture dishes in 1 mL Methocult H4230 medium (STEMCELL Technologies) supplemented with 2% FBS, 10 μg/mL 100× antibiotic-antimycotic solution (Sigma), 50 ng/mL SCF, 20 ng/mL IL-3, and 20 ng/mL rhG-CSF, or 100 ng/mL designed agonists. Cells were cultured at 37°C and 5% CO_2_. Colonies were counted on day 14.

### RNA-seq analysis

NFS-60 cells were washed two times with NFS-60-medium (RPMI 1640 medium with additional 1 mM l-glutamine, 1 mM Na-pyruvate, 10% FCS, 1% antibiotic-antimycotic [Gibco, catalog no. 15240062 ]) and starved at 300,000 cells/mL for 16 h at 5% CO_2_ and 37°C. We stimulated 1.5 million cells per sample in a T25 cell-culture flask with 1 nM of the selected design or rhG-CSF for 8 h. Subsequently, the cells were washed twice with PBS, lysed in 350 μL RTL buffer, and stored at −70°C until RNA extraction. At least 500 ng RNA per sample was used to prepare libraries for RNA-seq. The RNA Integrity Number (RIN) for RNA quality assessment was measured using a 2100 Bioanalyzer (Agilent). All RNA samples showed RIN >9.6 (maximum = 10), demonstrating the high quality of the RNA samples. Directional mRNA library preparation (poly(A) enrichment) and sequencing were done by Novogene (https://www.novogene.com). The libraries were sequenced on an Illumina NovaSeq 6000 using a paired-end 150-bp sequencing mode with a minimum of 6 Gb of raw data per sample. For data analysis, nf-core/rnaseq (https://nf-co.re/rnaseq^99^) was used, an RNA-seq analysis pipeline that takes a sample sheet and FASTQ files as input, performs quality assessment, trimming, and alignment, and produces a gene count (Table S1) and extensive quality control report. The nf-core/rnaseq pipeline was run with the following parameters: --profile ‘docker,' --genome ‘GRCm38,' --aligner ‘star_salmon,' pipeline version 3.10. Raw sequencing read counts were analyzed with the R package edgeR version 3.42.2.^100^ Of the initial 45,706 transcripts, 11,801 showed a sufficient level of expression. Normalized log2 cpm values were used for principal-component analysis and the descriptive part of the analysis. Differential expression was determined by fitting a quasi-likelihood negative binomial generalized log-linear model to count data. Resulting candidate genes that passed thresholds of absolute log2 FC >1 and FDR <0.05 were tested for gene set enrichment using the R package clusterProfiler version 4.8.1.^101^ Two gene set collections were used: GO terms and the MSigDB m2 mouse collection version 2022.1.

## Data availability

All relevant data for this article are provided in the supplemental files. The flow cytometry data are available at the FlowRepository database under the identifier FR-FCM-Z8CH (https://flowrepository.org/id/FR-FCM-Z8CH). Any additional intermediary data are available upon request.

## Acknowledgments

This project received funding from the 10.13039/501100012318IMPRS (T.U. and K.M.), the 10.13039/501100000781European Research Council under the European Union’s Horizon 2020 research and innovation program (grant agreement no. 863952 [ACE-OF-SPACE]) (P.M.), the M. Schickedanz Kinderkrebsstiftung (M.E., J.S., and N.A.), 10.13039/501100001659Deutsche Forschungsgemeinschaft (DFG, no. 500215849—M.E., B.H.-A., J.S., and V.H.; DFG, no. PI 405/15-2 – 326558201; and SFB, no. 1557, P13 – 467522186—J.P.), the 10.13039/501100005677José Carreras Leukämie-Stiftung (DJCLS10R/2024—M.E. and J.S.), internal funds from the 10.13039/501100004189Max Planck Society (A.L.), BMBF MyPred (J.S.), and the InnoChron COST EU action (J.S.). The authors would like to thank Luis Ángel Fernández for providing the bacterial display system plasmid and Regine Bernhard, Gabriele Hikade, and Hella Kenneweg for technical assistance.

## Author contributions

Conceptualization, T.U., K.W., P.M., J.S., and M.E.; methodology, T.U., N.A., J.P., J.S., and M.E.; investigation, T.U., C.P., M.E.-R., M.R., J.H., N.A., I.T., V.H., K.M., S.K., and M.K.; resources, C.L., B.H.-A., P.M., A.L., J.P., J.S., and M.E.; supervision, P.M., A.L., J.P., J.S., and M.E.; funding acquisition, P.M., A.L., J.P., J.S., and M.E.

## Declaration of interests

The designed agonist templates described in this study are originally described in WO2021123033A1 (inventors: B.H.-A., M.E., and J.S.), which was filed by Eberhard Karls Universität Tübingen and Max-Planck Gesellschaft zur Förderung der Wissenschaften e.V. M.E. is a co-founder of Heliopolis Biotechnology.

## Supplemental information

Document S1. Figures S1–S24 and Tables S2–S4

Table S1. Counts matrix for RNA-sequencing reads

Document S2. Article plus supplemental information
