# Supplementary material for: Tuning of G-CSFR signaling by de novo-designed agonists
Source: Mol Ther. 2025 Aug 29;33(11):5741–59. doi: 10.1016/j.ymthe.2025.08.031 (PMC12628175; doi:10.1016/j.ymthe.2025.08.031)
Supplement: Document S1. Figures S1–S24 and Tables S2–S4 [file mmc1.pdf]

YMTHE, Volume *33*

Supplemental Information

Tuning of G-CSFR signaling

by *de novo*-designed agonists

Timo Ullrich, Christoph Pollmann, Malte Ritter, Jérémy Haaf, Narges Aghaallaei, Ivan Tesakov, Valeriia Hatskovska, Maya El-Riz, Kateryna Maksymenko, Sergey Kandabarau, Maksim Klimiankou, Claudia Lengerke, Karl Welte, Birte Hernandez-Alvarez, Patrick Müller, Andrei Lupas, Jacob Piehler, Julia Skokowa, and Mohammad ElGamacy

Supplemental figures

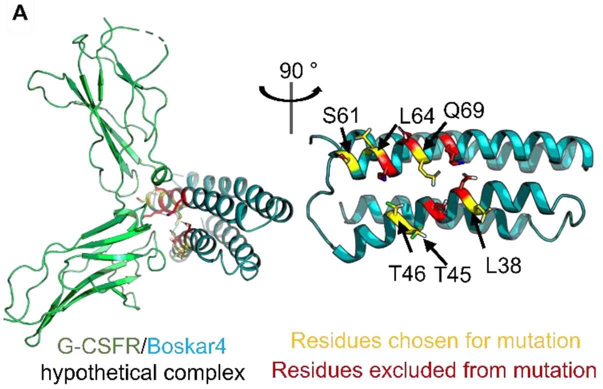

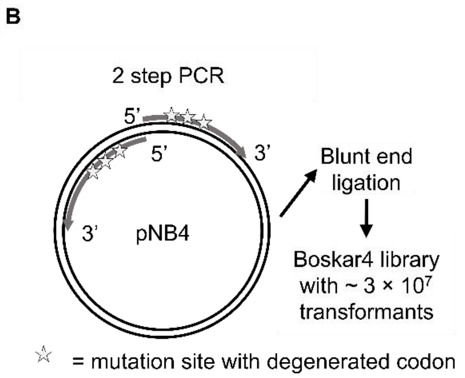

C

Boskar4 saturation library

Position (in 7NY0)	Codons (reverse complement)	Coded amino acids
L38	NDT (AHN)	F, L, I, V, Y, H, N, D, C, R, S, G
T45	NNT (ANN)	F, L, I, V, S, P, T, A, Y, H, N, D, C, R, G
T46	NNT (ANN)	F, L, I, V, S, P, T, A, Y, H, N, D, C, R, G
S61	NDT	F, L, I, V, Y, H, N, D, C, R, S, G
L64	NDT	F, L, I, V, Y, H, N, D, C, R, S, G
Q69	VDG	L, M, V, Q, K, E, R, G

Theoretical diversity	4.0E+06
Amino-acid diversity	3.1E+06
Total transformants	3.5E+07

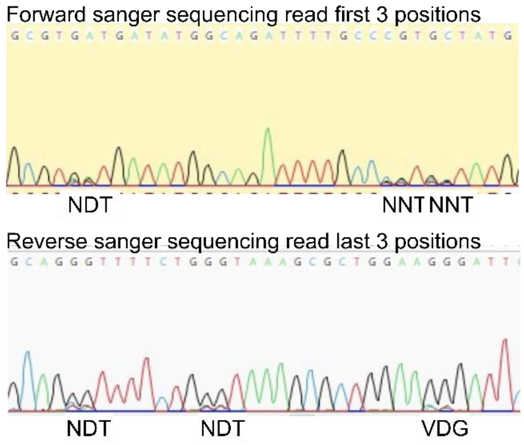

D

Boskar4 library designed with Damietta

Position (in 7NY0)	aa lowest energy change	Codons (reverse complement)	Present
L38	R, F, L, N, I	CKA, WWC (TMG, GWW)	F(*) I(*) L(*) N(*) R(*) Y(0)
T45	Y, F, R, N, H	HNC (GND)	C(0) F(*) H(*) I(0) L(0) N(*) P(0) R(*) S(0) T(*) Y(*)
T46	N, L, D, I, S	RAC, WYA (GTY, TRW)	D(*) I(*) L(*) N(*) S(*) T(*)
S61	F, Y, R, D, N	NDC	C(0) D(*) F(*) G(0) H(0) I(0) L(0) N(*) R(*) S(*) V(0) Y(*)
L64	Y, F, R, H, S	YNC	C(0) F(*) H(*) L(*) P(0) R(*) S(*) Y(*)
Q69	M, K, L, V, I	MWA, RTG	I(*) K(*) L(*) M(*) Q(*) V(*)

Theoretical diversity	2.5E+05
Amino-acid diversity	2.3E+05
Total transformants	2.7E+07

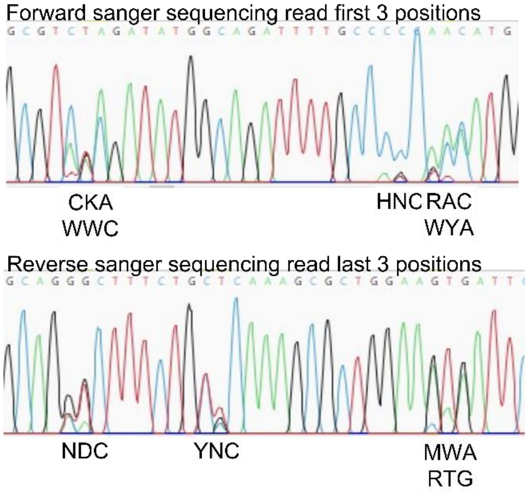

(*) = wanted aa (top 5 aa at the given positions or "wt"-aa) (over all 15.63 %)
(0) = unwanted aa (aa added because of used degeneration)

Figure S1. (A) Side view on Boskar4 (cyan) in a modeled complex with G-CSFR (green) and detailed view on Boskar4 with marked residues based on 7NY0 considered for mutagenesis (yellow) based on the

minimal distance to G-CSFR residues. Residues that were thought to be critical for G-CSF activity (red) regarding to Young *et al.* [1] were excluded from mutagenesis also if they fulfilled the distance criteria. **(B)** Overview of library generation via degenerated codons and blunt end ligation. **(C)** Overview of the saturation library of Boskar4 for affinity maturation. **(D)** Overview of the Damietta *in silico* designed library of Boskar4 which was estimated from a reduced set of the same residues as the saturation library.

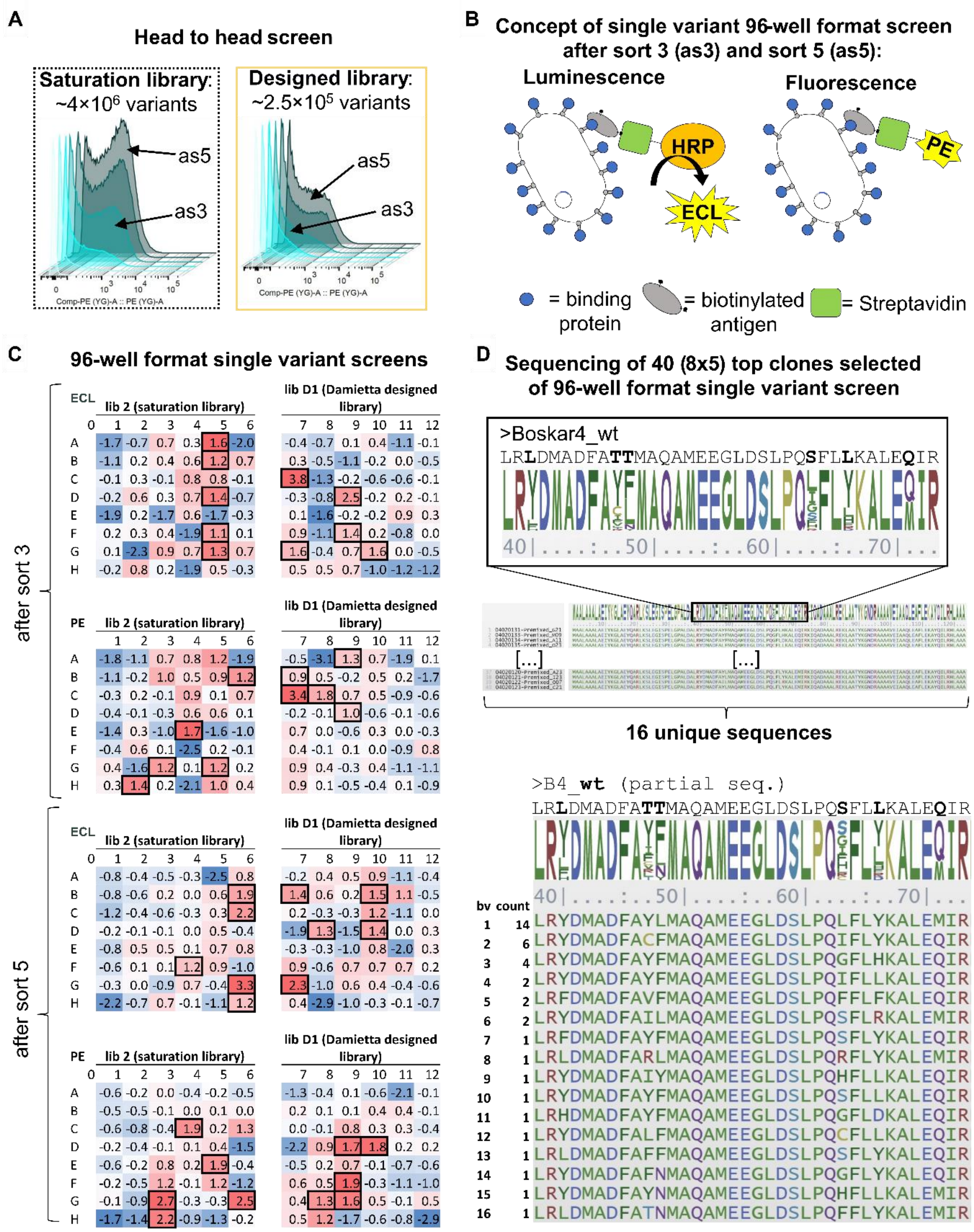

Figure S2. (A) Fluorescence measured with FACS of bacteria carrying either the saturation library (dotted line) or the Damietta designed library (light orange) over 5 steps of enrichment (light cyan to dark gray) in a head-to-head screening experiment. **(B)** Concept of single variant 96-well format screen after sort 3 (as3) and sort 5 (as5). Two different readout methods were utilized to estimate the relative binding activity of each variant. Luminescence was applied by a horse radish peroxidase (HRP) avidin conjugate with

enhanced chemiluminescence substrate (ECL), and fluorescence was measured by streptavidin phycoerythrin (PE) conjugate. **(C)** Results of the 96-well format single variant screens after enrichment of the library with FACS. Each value corresponds to the z-score given by a single variant (single well on the plate) to all other variants from the same condition. In total, 8 such screens were made under different conditions (PE or ECL, after sort 3 or after sort 5, saturation or designed library), and top 5 clones of each condition were picked and sequenced. **(D)** Sixteen unique Boskar4 variants (bv1 to bv16) were identified among the 40 analyzed.

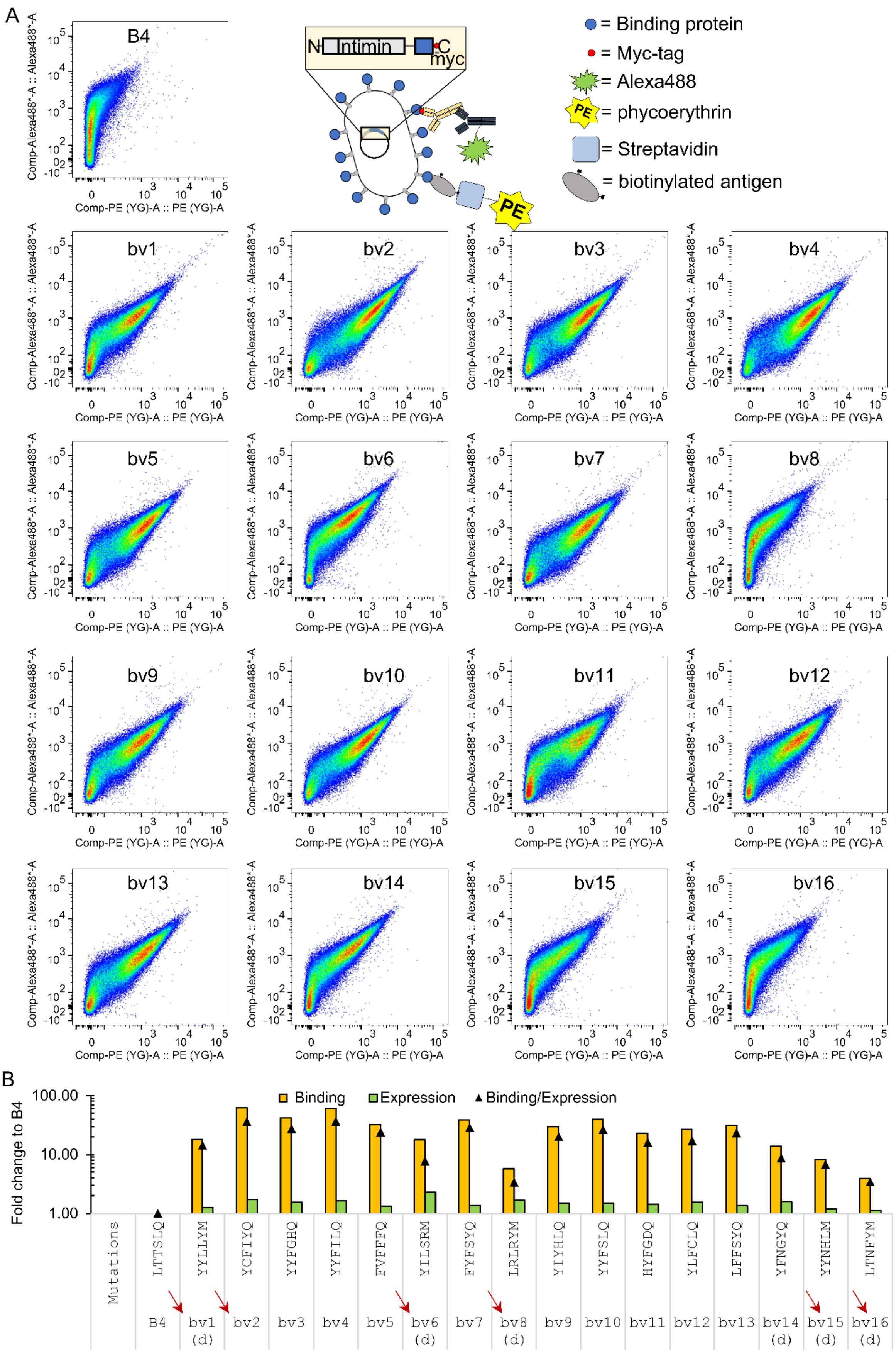
A
N Intimin C
myc
= Binding protein
= Myc-tag
= Alexa488
PE = phycoerythrin
= Streptavidin
= biotinylated antigen
B4
bv1
bv2
bv3
bv4
bv5
bv6
bv7
bv8
bv9
bv10
bv11
bv12
bv13
bv14
bv15
bv16
Comp-Alexa488*-A :: Alexa488*-A
Comp-PE (YG)-A :: PE (YG)-A
B
Binding
Expression
Binding/Expression
Fold change to B4
100.00
10.00
1.00
Mutations
LTTSLQ
YYLLYM
YCFIYQ
YYFGHQ
YYFILQ
FVFFFQ
YILSRM
FYFSYQ
LRLRYM
YIYHLQ
YYFSLQ
HYFGDQ
YLFCLQ
LFFSYQ
YFNGYQ
YYNHLM
LTNFYM
B4
bv1 (d)
bv2
bv3
bv4
bv5
bv6 (d)
bv7
bv8 (d)
bv9
bv10
bv11
bv12
bv13
bv14 (d)
bv15 (d)
bv16 (d)

Figure S3. (A) The 16 new affinity-enhanced Boskar4 variants (bv1 to bv16) and Boskar4 (B4) were analyzed on the surface of *E. coli* by FACS for their binding activity to BioGCSFR (10 nM, PE channel) and the expression level of the corresponding variant (anti-myc labeling, Alexa488 channel). **(B)** Based on the measurements in (A) the binding to G-CSFR (orange bars), expression of the binder (green bars), and expression-normalized binding to G-CSFR (black triangles) of the affinity-enhanced Boskar4 variants normalized to Boskar4 was analyzed. Sequences selected for further characterization are marked with dark red arrows. The small "d" in parentheses (d) indicates that the variant was selected from the designed library. Additionally, the corresponding mutation set of each enhanced variant is indicated in comparison to Boskar4.

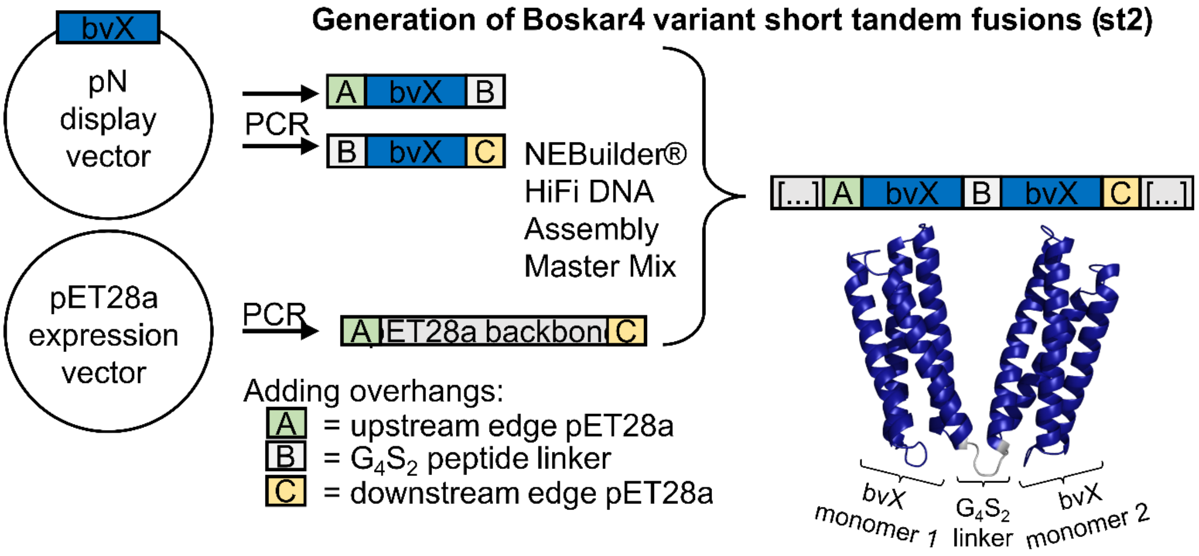

Figure S4. The Boskar4 variant short tandem fusions (st2) were generated by the assembly of three fragments consisting of the expression vector, two copies of a Boskar4 variant (bvX) and an overlap including the G_4S_2 peptide linker. The same approach was also used to generate the rigid tandem fusions (ori0 to ori4) by encoding a rigid helix linker instead of a flexible GS-peptide-linker.

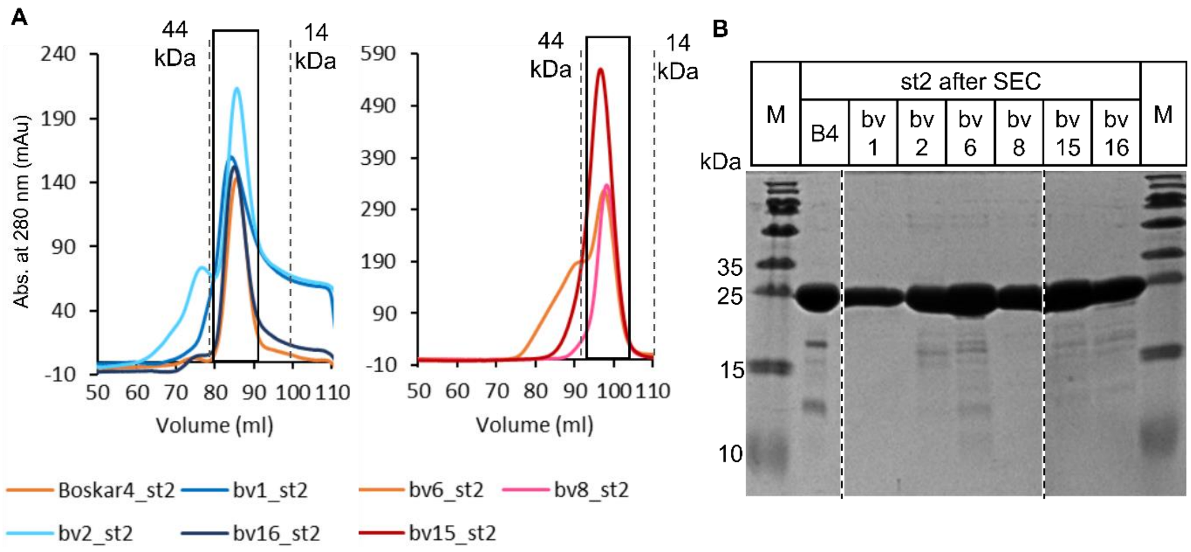

Figure S5. (A) Preparative size exclusion chromatography of Boskar4 short tandem fusion (st2) variants after Nickel IMAC on two separate SEC-columns of the same type (HiLoad 16/600, Superdex 200 pg). Dotted lines mark the molecular weight standard for the corresponding column. All fractions of the main peak (black box) were collected and concentrated by ultrafiltration (10 kDa cut-off). **(B)** A representative sample of each protein and a protein molecular weight marker (M) (11852124, Thermo Fisher Scientific) was loaded on a SDS-PAGE (18% PA, 150 V, 1 h) which was subsequently stained with Coomassie. The expected molecular weight of Boskar4_st2 and variants (with not cleaved TEV-cleavage site and His-tag) is ~29 kDa.

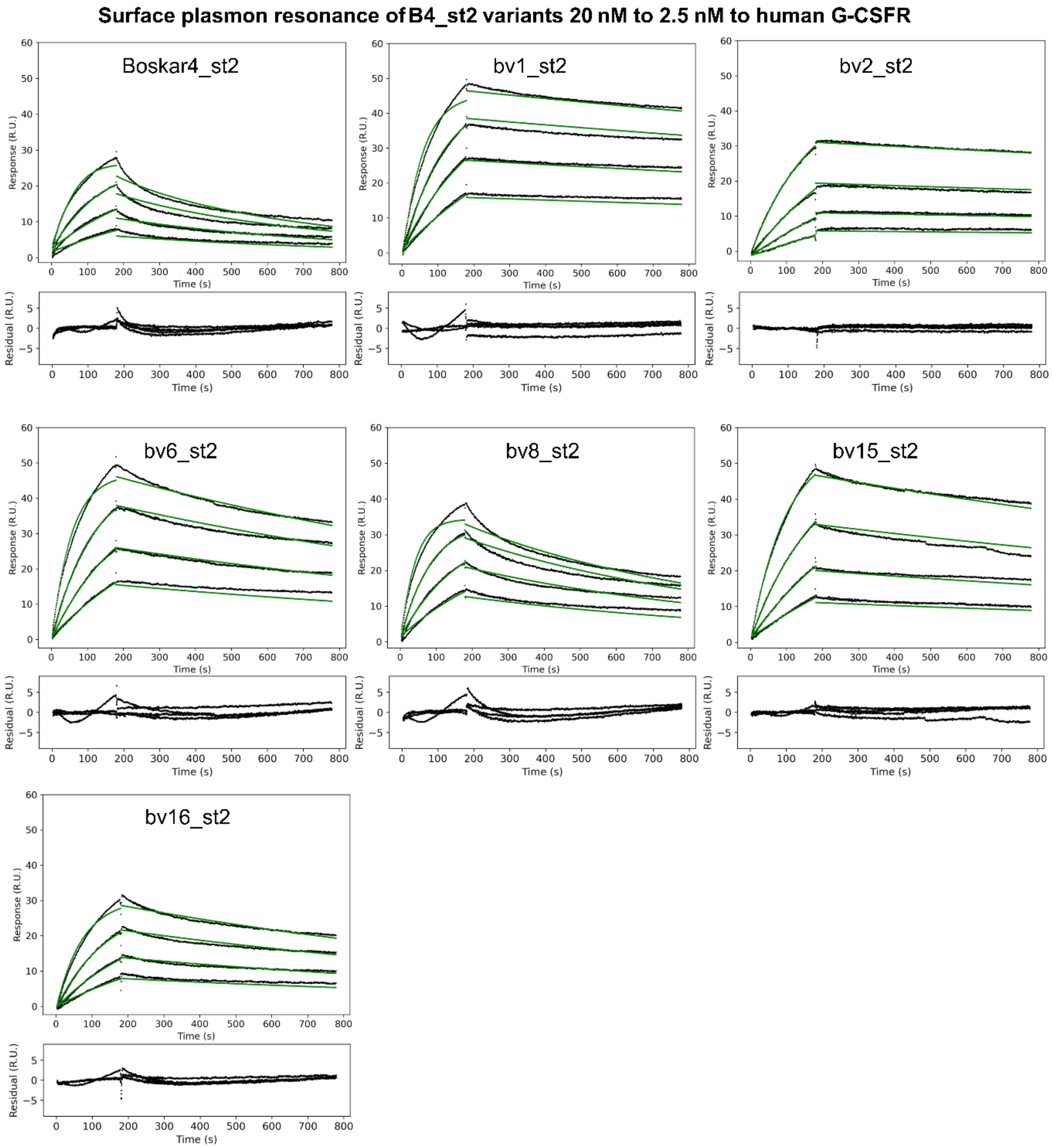

Figure S6. Six of the enhanced variants and Boskar4 itself (B4) were chosen to generate short tandem fusions (st2) and measured with surface plasmon resonance (SPR) against immobilized hG-CSFR. The association rate constant (k_a), the dissociation rate constant (k_d), and the apparent equilibrium dissociation constant (K_D) were estimated from the corresponding fits (green lines) of a two-fold titration series from 20 nM to 2.5 nM (compare Table 1).

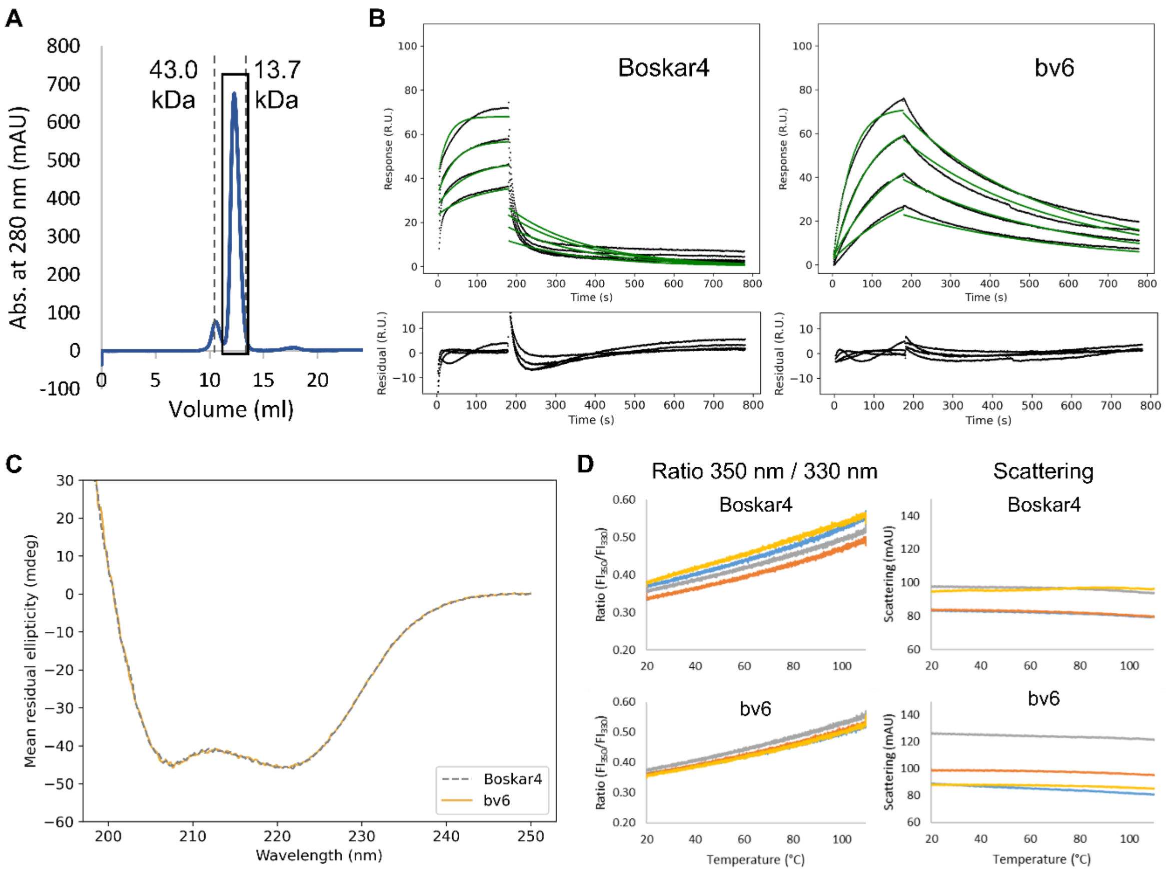

Figure S7. (A) Preparative size exclusion chromatography of monomeric bv6 on a Superdex® 75 10/300 GL with an expected size of ~15 kDa. Dotted lines mark the molecular weight standard for the corresponding column. All fractions of the main peak (black box) were collected and concentrated by ultrafiltration (10 kDa cut-off). **(B)** Surface plasmon resonance measurement (black line) and the corresponding fit (green lines) of a two-fold dilution series of monomeric Boskar4 and the variant bv6. The highest concentration used for Boskar4 was 1250 nM and for bv6 125 nM. The obtained binding parameters are listed in Table 1. **(C)** The single-domain Boskar4 (B4; gray) and an example variant (bv6; orange) were analyzed with circular dichroism and show strong helical signal. **(D)** Thermostability measurement with nanoDSF of 1 mg/mL of monomeric Boskar4 and bv6 with 4 technical replicates, each indicating the proteins to be stable to at least 110 °C.

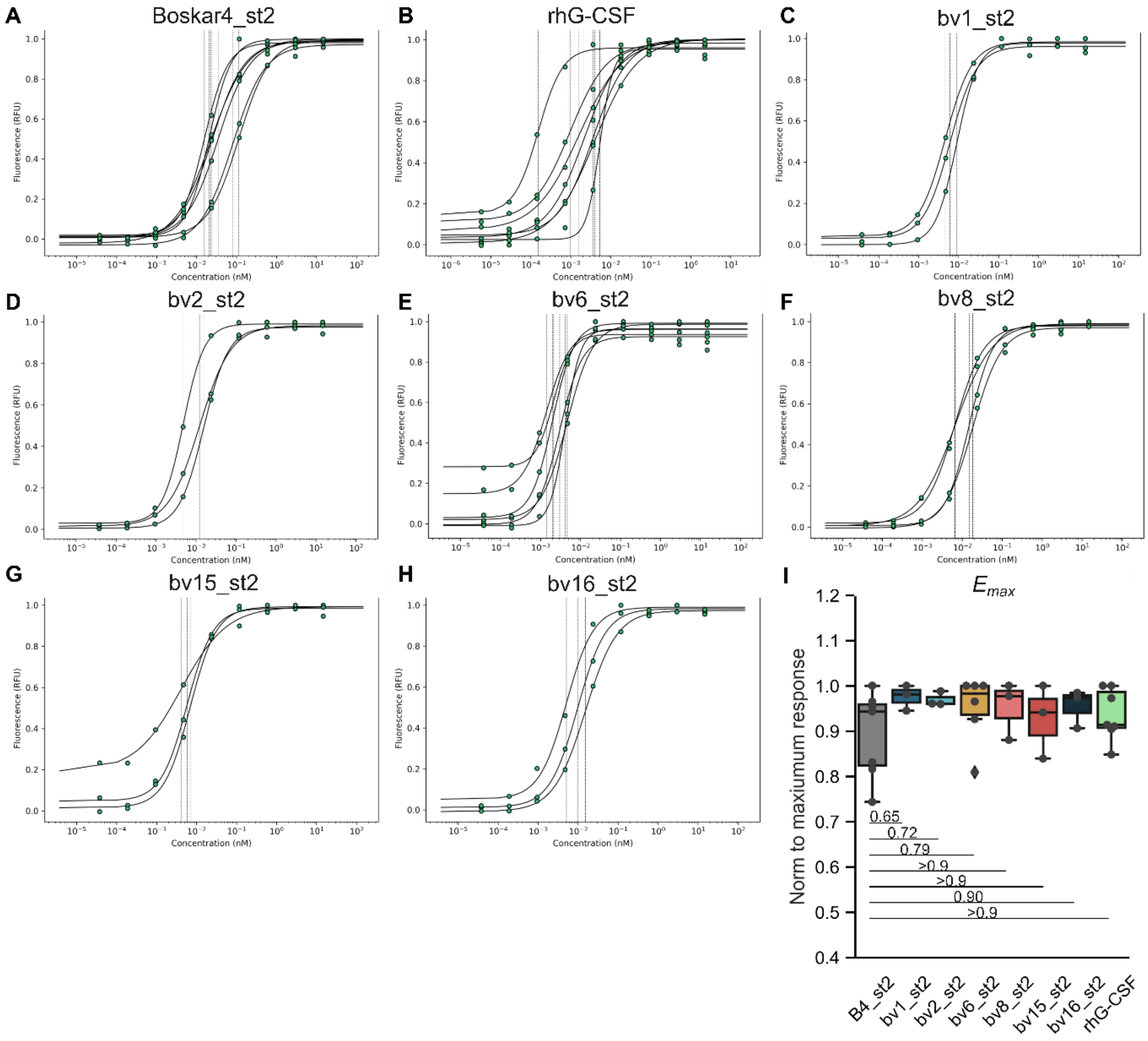

Figure S8. (A-H) Dose-response NFS-60 cell proliferation assays to determine the half-maximal effective concentration (*EC_{50}*) of Boskar4_st2 variants and human recombinant G-CSF (hrG-CSF). The fits to determine the *EC_{50}* (pM) of at least three independent experiments per G-CSFR agonist are shown (compare also Table 1 and Fig. 2C). **(I)** Maximum proliferative activity (*E_{max}*) normalized to the maximum response within each independent experiment represented in (A-H). For statistical analysis, an ordinary one-way ANOVA was performed, followed by a Tukey HSD test.

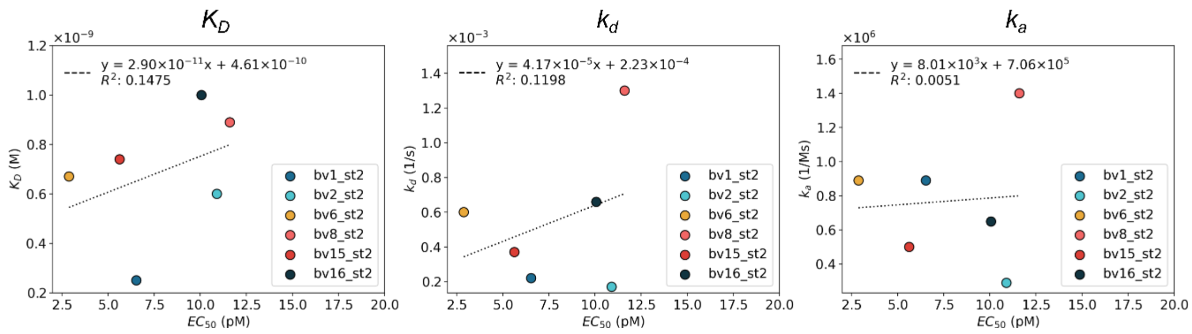

Figure S9. Shown is the correlation of a linear fit between the apparent affinity (K_D), association rate constant (k_a) or dissociation rate constant (k_d) and the obtained NFS-60 activity (EC_{50}) for short tandem fusions of Boskar4 (B4) or the affinity-enhanced variants (bv1, bv2, bv6, bv8, bv15, bv6). The coefficient of determination (R^2) is shown in the left upper corner.

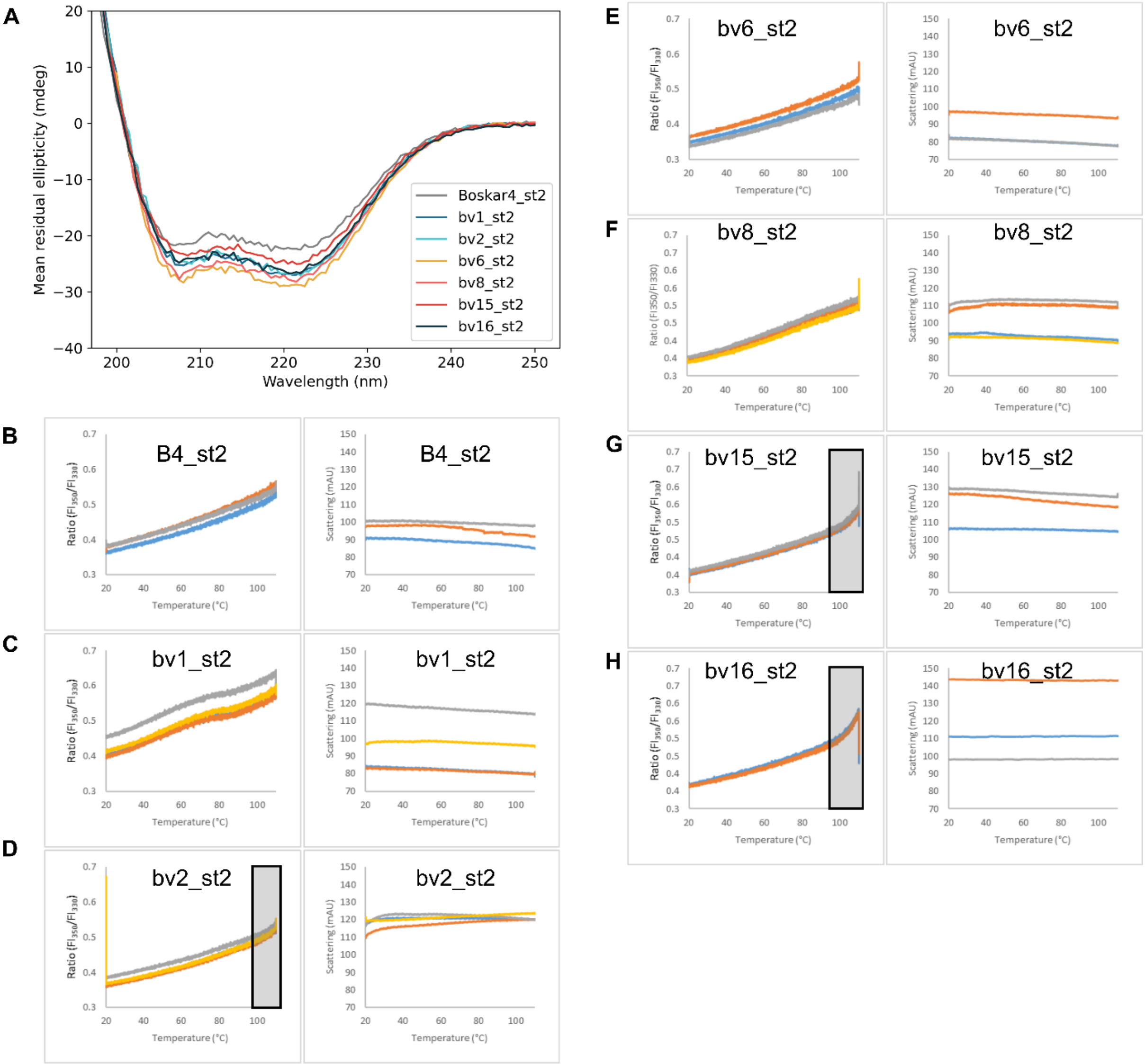

Figure S10. (A) Circular dichroism (CD) measurement of Boskar4_st2 and variants (bv1 to bv16) in PBS at 0.1 mg/mL shows expected alpha-helical signal for all samples. **(B-H)** Thermostability measurement with nanoDSF of Boskar4_st2 (B4_st2) and variants (bv1_st2 to bv16_st2) with at least 3 technical replicates. All samples were diluted to 1 mg/mL in PBS, except for bv1_st2, which was used at a concentration 0.5 mg/ml. The gray boxes indicate areas in which a melting onset was observed.

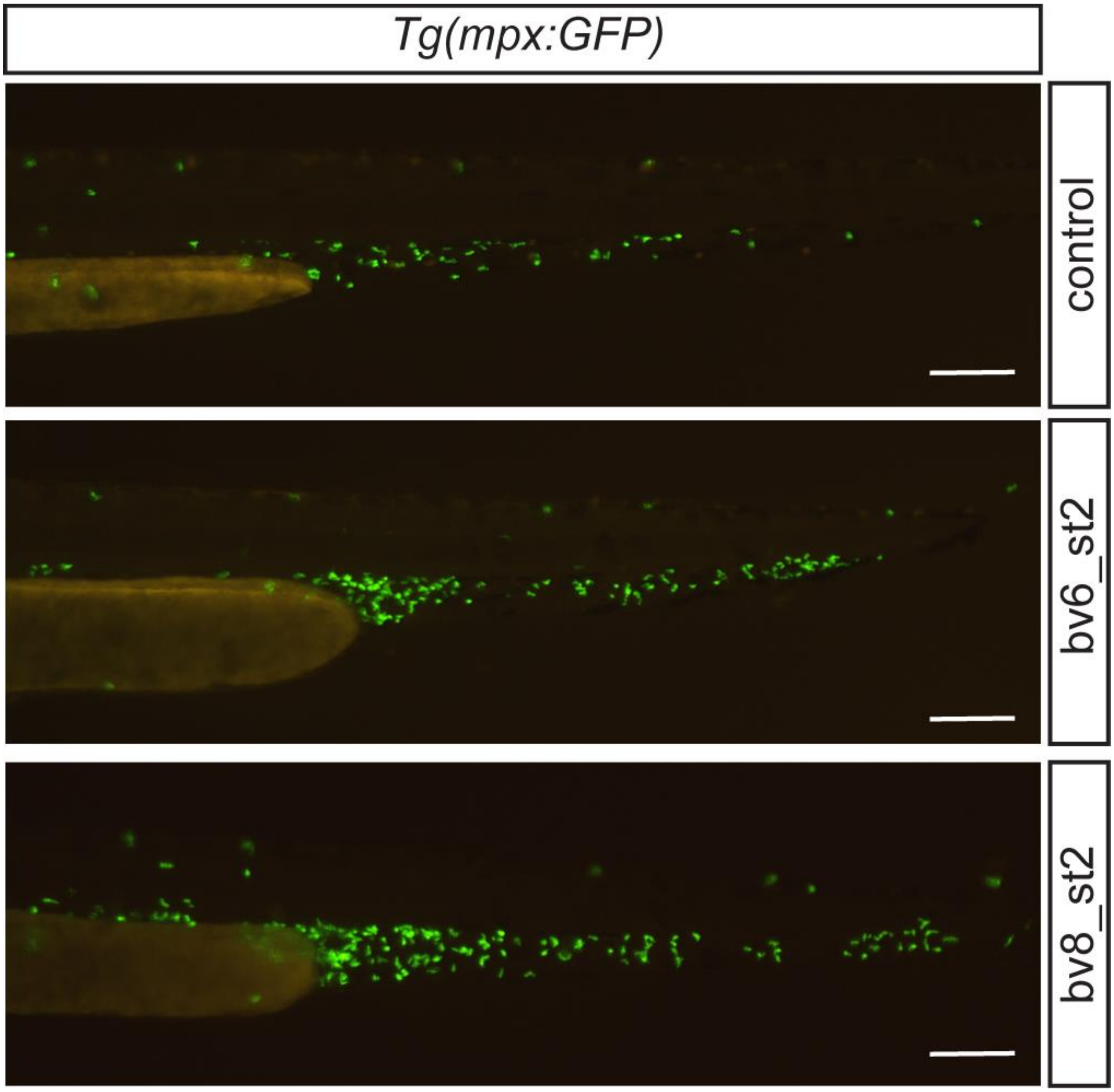

Figure S11. Representative images of transgenic zebrafish embryos Tg(mpx:GFP) treated by either Moevan_control (4 mg/mL), bv6_st2 (2 mg/mL), or bv8_st2 (2 mg/mL) at 24-hours post-injection (a single 4 nL injection). The numbers of visible GFP-expressing neutrophils in the tail region are elevated in bv6_st2- and bv8_st2-treated embryos. Scale bars, 200μm.

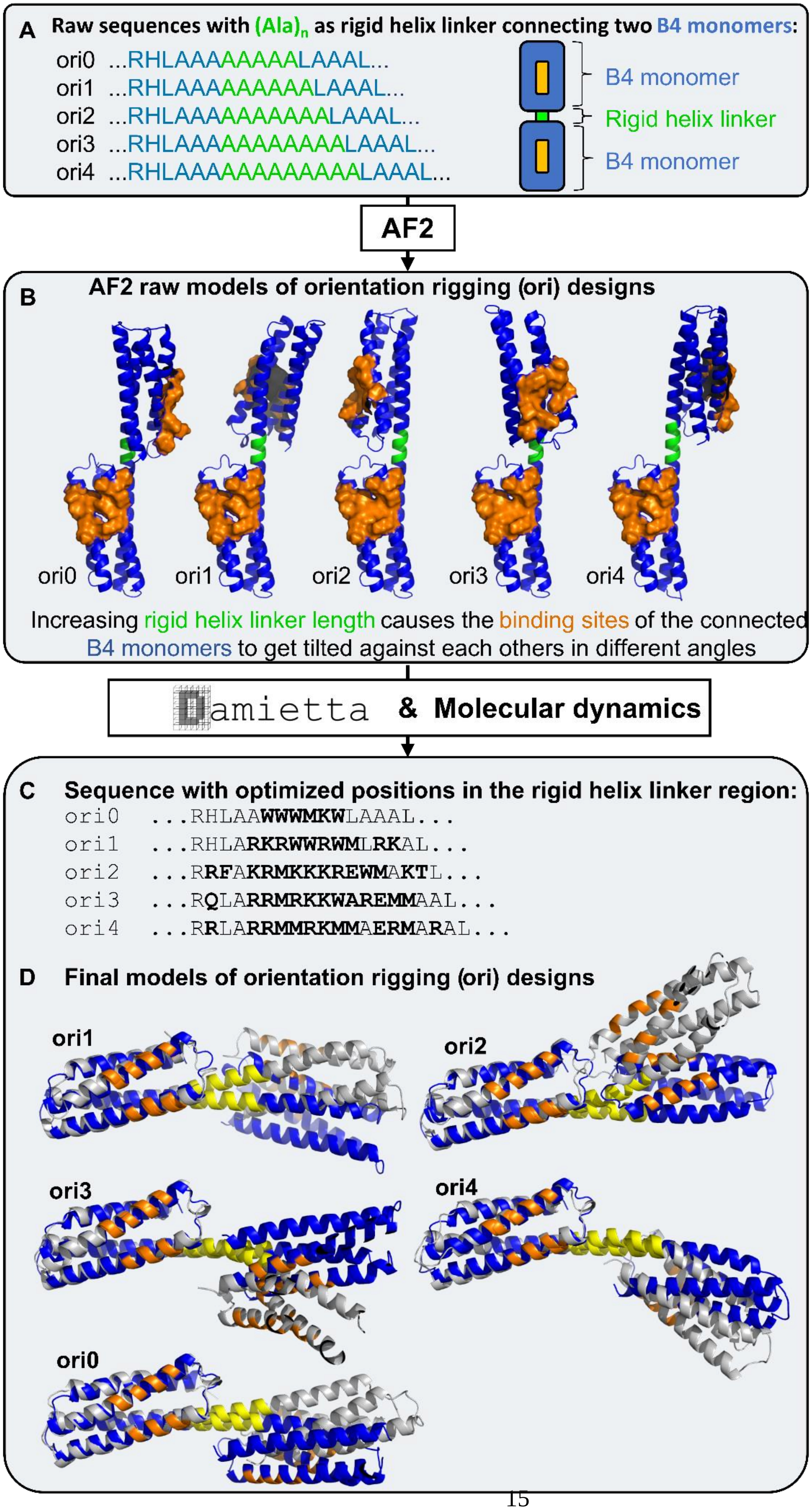
A Raw sequences with (Ala)n as rigid helix linker connecting two B4 monomers:
ori0 ...RHLAAAAAAAALAAAL...
ori1 ...RHLAAAAAAAAALAAAL...
ori2 ...RHLAAAAAAAAAALAAAL...
ori3 ...RHLAAAAAAAAAAALAAAL...
ori4 ...RHLAAAAAAAAAAAALAAAL...
B4 monomer
Rigid helix linker
B4 monomer
AF2
B AF2 raw models of orientation rigging (ori) designs
ori0
ori1
ori2
ori3
ori4
Increasing rigid helix linker length causes the binding sites of the connected B4 monomers to get tilted against each others in different angles
Damietta & Molecular dynamics
C Sequence with optimized positions in the rigid helix linker region:
ori0 ...RHLAAWWWMKWLAAAL...
ori1 ...RHLARKRWWRWMLRKAL...
ori2 ...RRFAKRMKKKREWMAKTL...
ori3 ...RQLARRMRKKWAREMMAAL...
ori4 ...RRLARRMMRKMMAERMARAL...
D Final models of orientation rigging (ori) designs
ori1
ori2
ori3
ori4
ori0

Figure S12. Generation of the orientation-rigging designs (oris) tuning G-CSFR activity. **(A)** Raw sequence design. **(B)** AlphaFold2 raw models. **(C)** Selected residues (bold) in the rigid helix linker region were optimized with Damietta, and the most stable designs were identified with molecular dynamics (MD). **(D)** The MD input structure of each top hit is displayed in blue, and the last frame of the MD-simulation is indicated in gray. The linker helix area is depicted in yellow, and the G-CSFR binding sites are highlighted in orange.

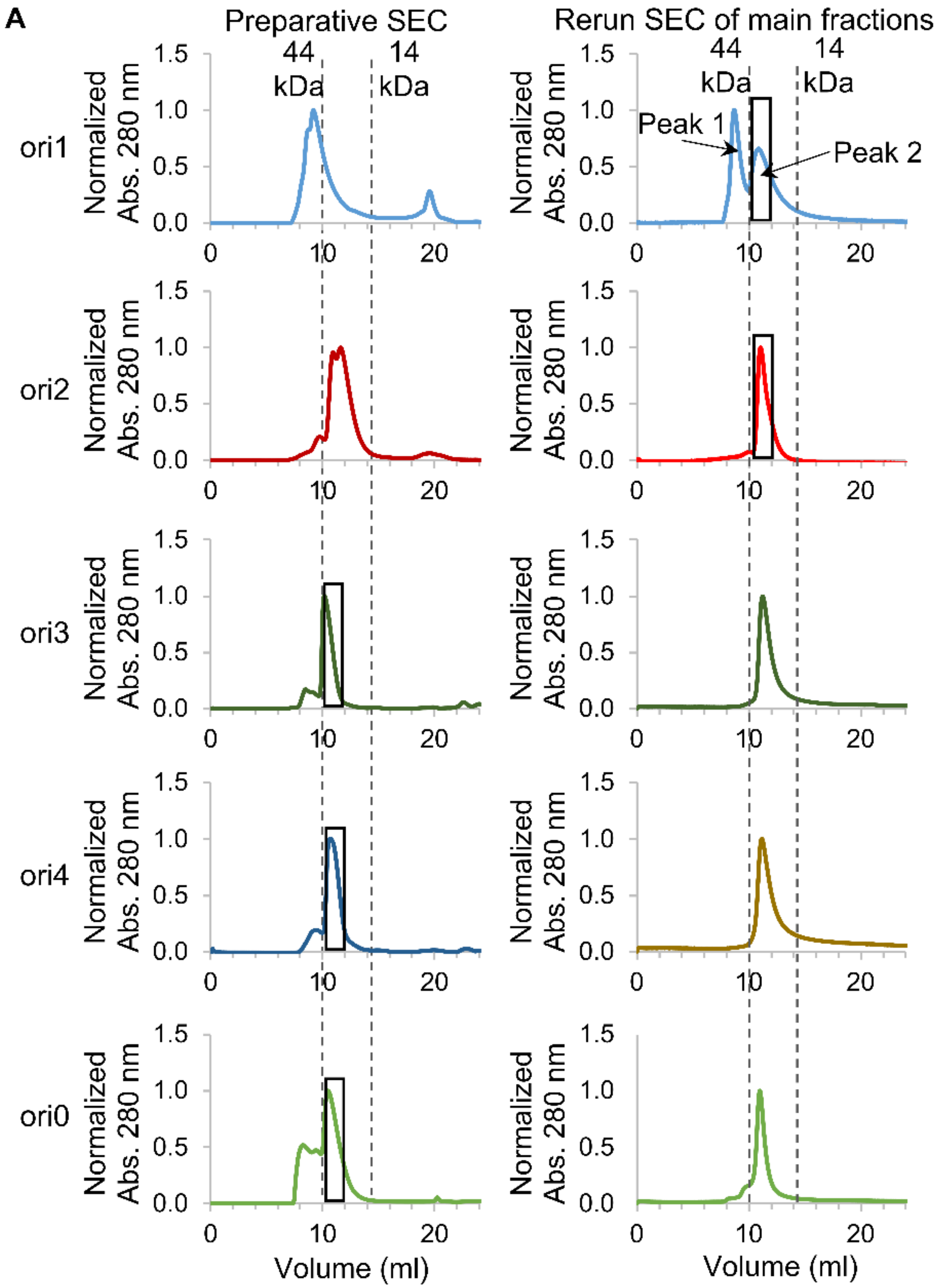

Figure S13. (A) Size exclusion chromatography of ori0, ori1, ori2, ori3 and ori4 after Nickel IMAC was performed on a Superdex Increase 75 10/300 GL (Cytiva). The dotted lines mark the molecular weight standard for the corresponding column. All fractions of the main peak were collected and concentrated by

ultrafiltration (10 kDa cut-off). Part of the collected fractions were rerun on the same column to validate the purity and correct molecular mass.

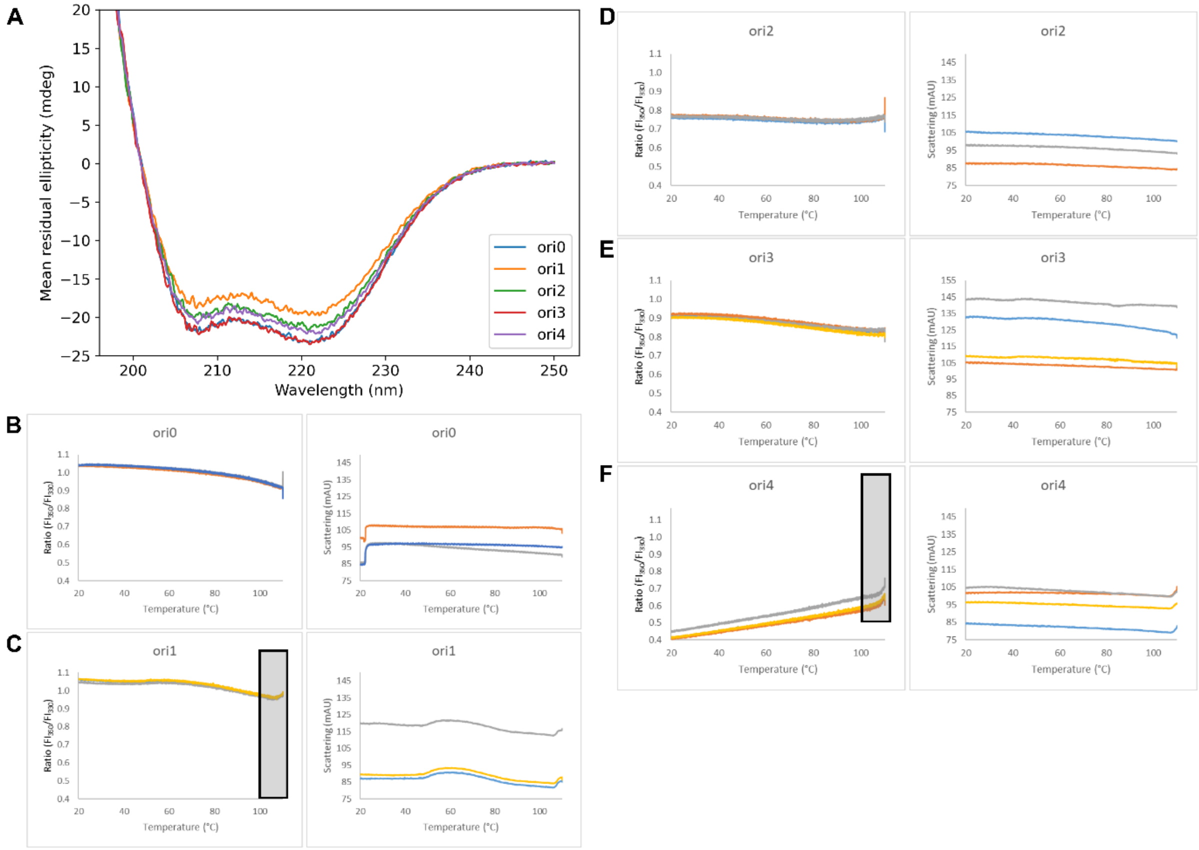

Figure S14: (A) Circular dichroism (CD) measurement of ori0, ori1, ori2, ori3 and ori4 in PBS at 0.1 mg/mL showed the expected alpha-helical signal for all samples. **(B-F)** Thermostability measurement with nanoDSF of the rigid tandem fusions (ori0 to ori4) with at least 3 technical replicates. All samples were diluted to 1 mg/mL in PBS. The gray boxes indicate areas in which a melting onset was observed.

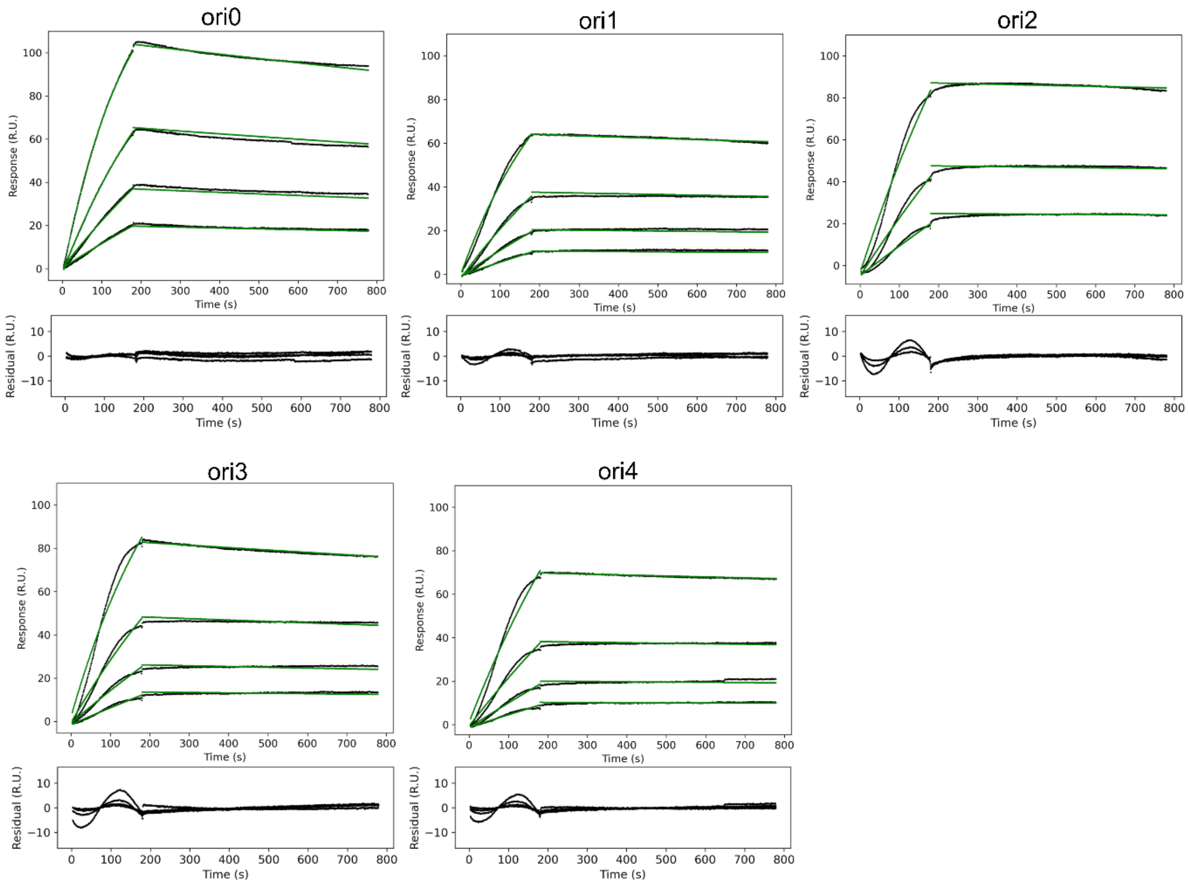

Figure S15. Surface plasmon resonance (SPR) measurement of oris against immobilized hG-CSFR. The association rate constant (k_a), the dissociation rate constant (k_d), and the apparent equilibrium dissociation constant (K_D) were estimated from the corresponding fits (green lines) of a titration series ranging from 20 nM to 2.5 nM (compare Table 1).

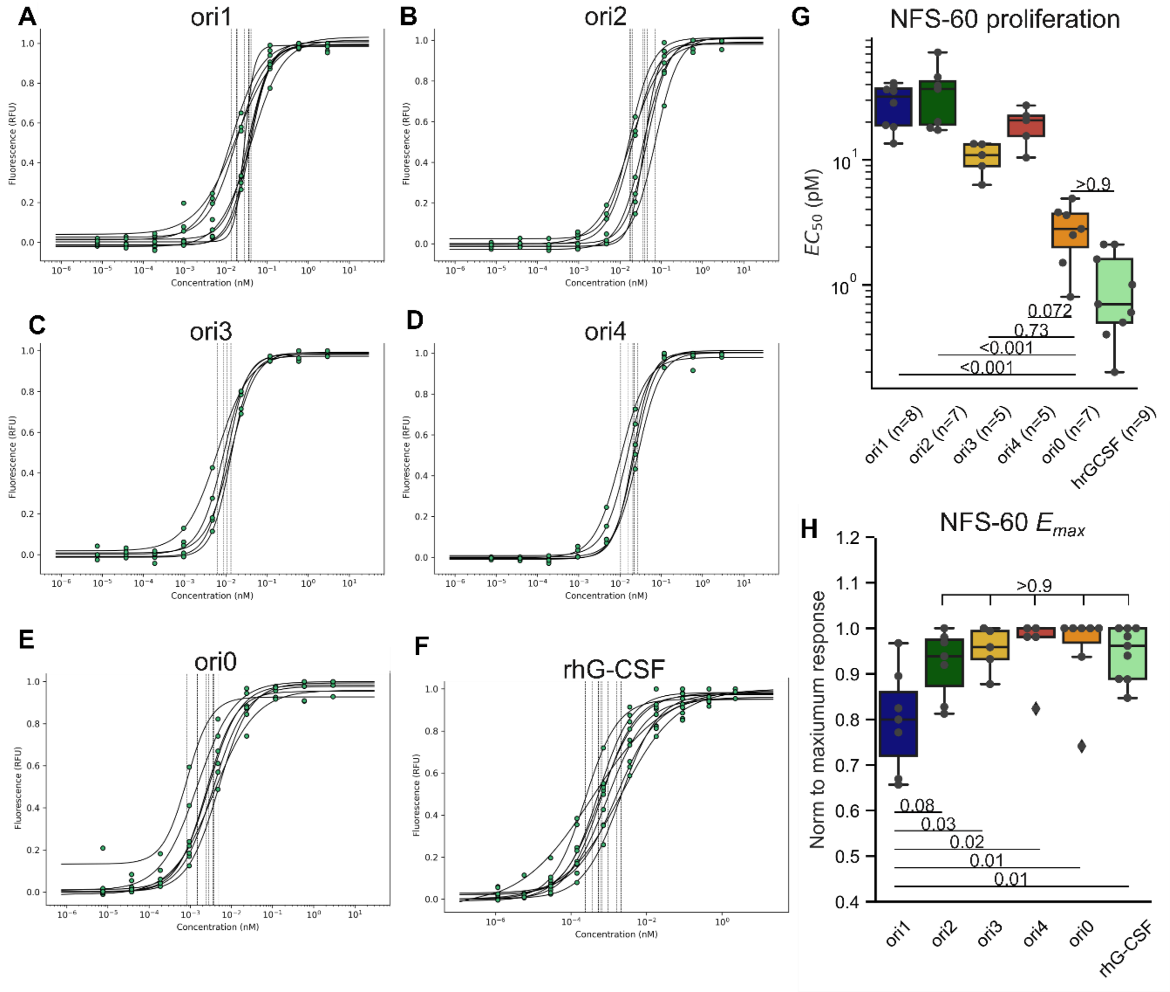

Figure S16. (A-F) Dose-response NFS-60 cell proliferation assays to determine the half-maximal effective concentration (EC_{50}) of the oris and G-CSF (rhG-CSF, Lenograstim). Given are the fits to determine the EC_{50} of at least five independent experiments per G-CSFR agonist (compare also Table 1 and Fig. 3C). **(G, H)** Box plot of the EC_{50} (pM) values (G) and the maximum proliferative activity (E_{max}) normalized to the maximum response (H) within each independent experiment represented in (A-F). For statistical analysis in (G) and (H), ordinary one-way ANOVA, followed by a Tukey HSD test was performed.

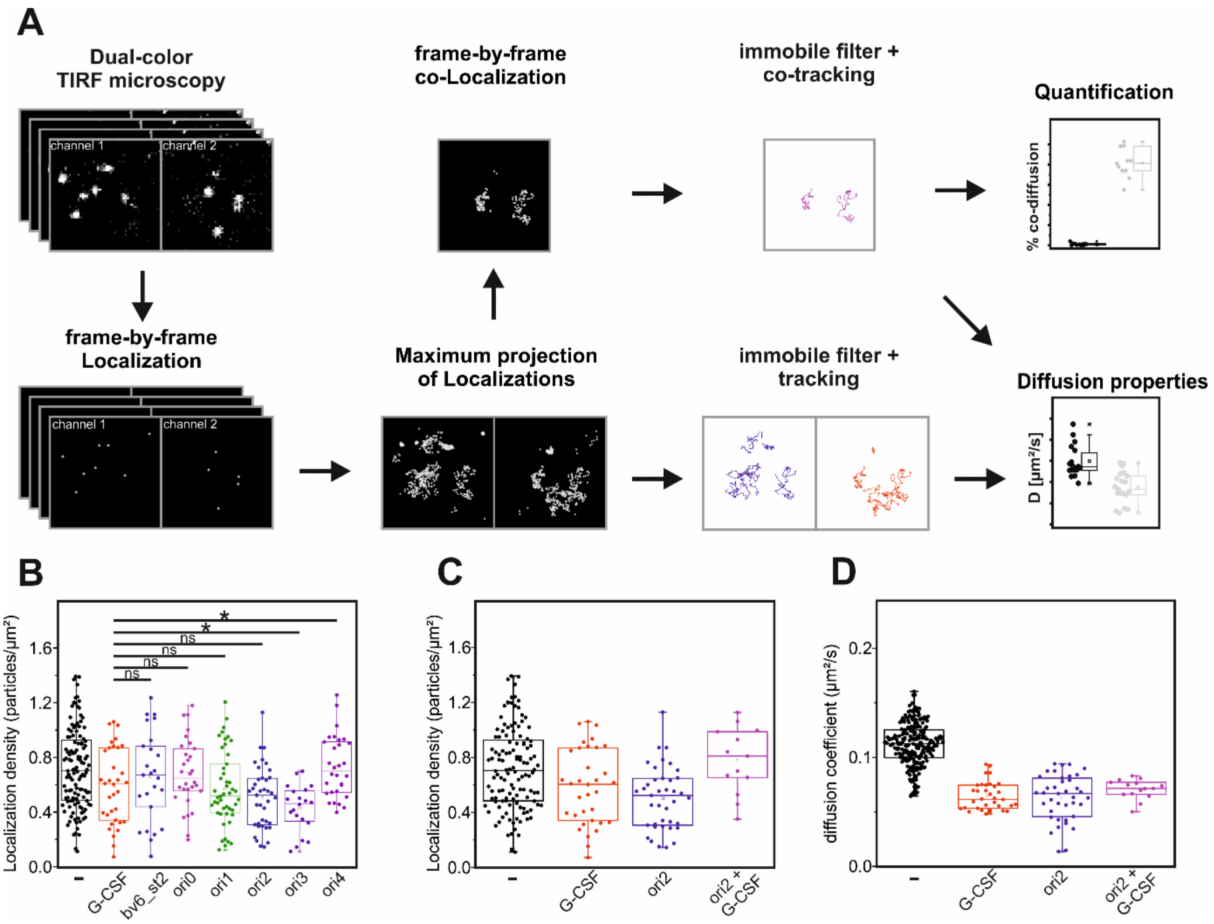

Figure S17. The dimerization of G-CSFR was analyzed with single molecule imaging for G-CSF and the design agonists. **(A)** Schematic representation of Single molecule co-tracking (SMCT) workflow. **(B,C)** Receptor density distribution for SMCT experiments. **(D)** Diffusion coefficients of G-CSFR and induced complexes in competition assay.

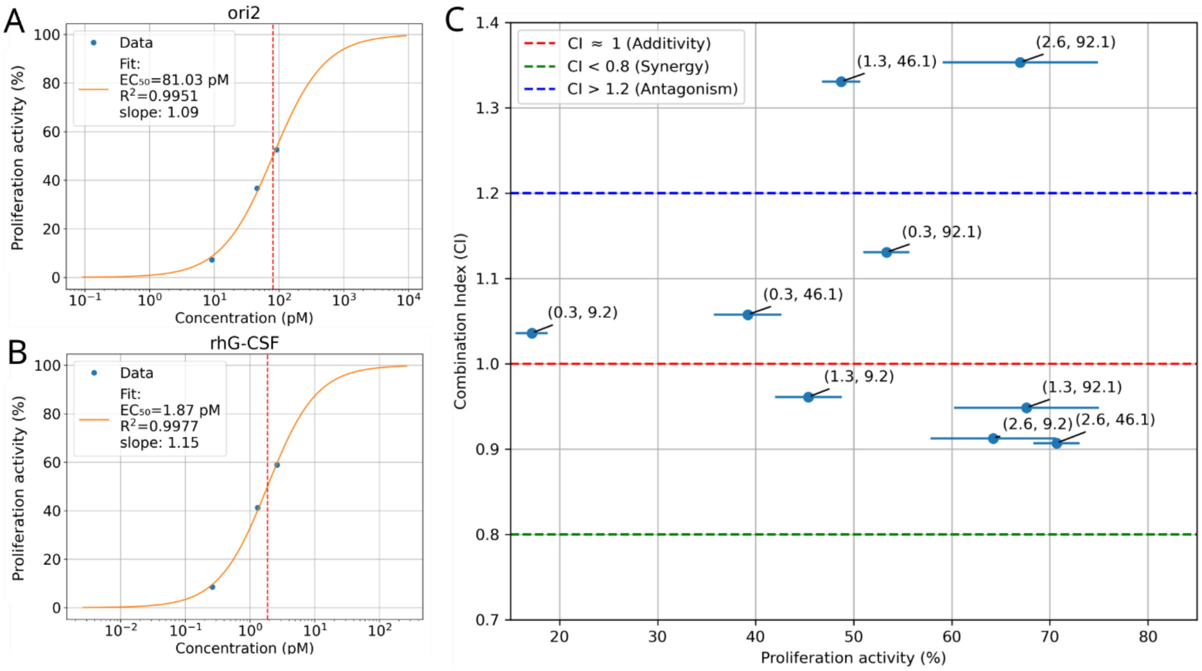

Figure S18. rhG-CSF and ori2 interact additively at low concentrations. (A, B) concentrations close to the proliferative EC_{50} values (in NFS-60 cells) of rhG-CSF and ori2 were first used to evaluate the effect combination treatment. **(C)** A matrix of combination treatments in NFS-60 (Fig. 4G) showed a largely additive effect, with the exception of two concentrations as judged by the corresponding combination index (CI; see Methods section). The concentrations (rhG-CSF (pM), ori2 (pM)) are indicated for each combination treatment, along with the standard deviation of the observed activities (blue horizontal lines). The dotted lines for additivity, synergy, and antagonism are representatively set at levels ± 20% of the additive line to distinguish clearer effects of synergy or antagonism.

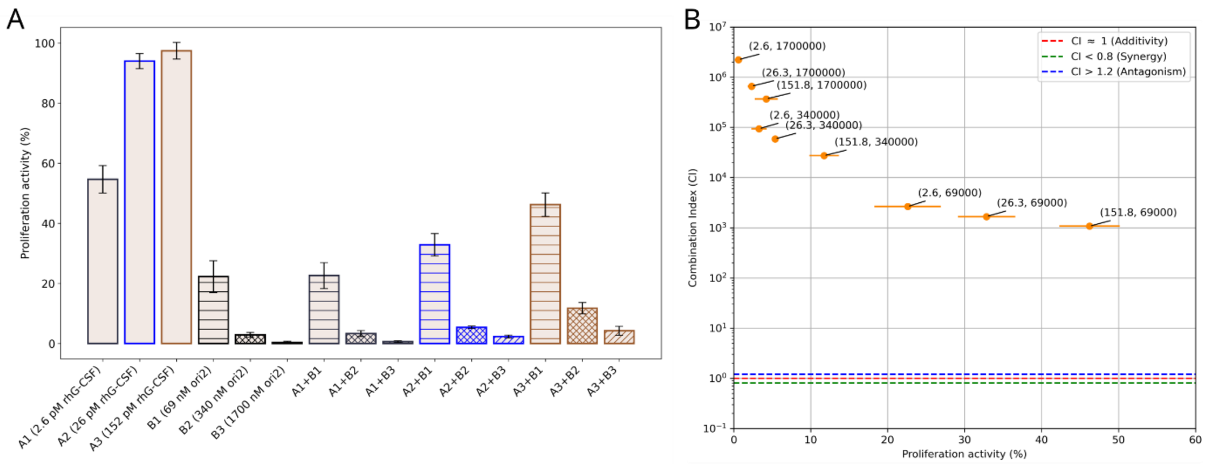

Figure S19. Higher concentrations of ori2 override rhG-CSF activity. (A) NFS-60 cell proliferation activity of rhG-CSF and ori2 for separate treatments (A1 to A3, and B1 to B3) and combined treatments (A1+B1, A2+B2, etc.) with self-inhibitory concentrations of ori2 (69 nM, 340 nM, and 1,700 nM) and G-CSF concentrations comparable to physiological levels (50 pg/mL), 10 times higher concentrations observed during bacterial infections (500 pg/mL), and the highest measured concentrations during sepsis (2,885 pg/mL). The plot shows the mean and standard deviation values of two independent experiments with three parallel replicates each. **(B)** The measured activities of the combination treatments shown in (A) were plotted against their corresponding combination index (CI). The concentrations (rhG-CSF (pM), ori2 (pM)) are indicated for each combination, along with the standard deviation of the observed activities (blue horizontal lines). The dotted lines for additivity, synergy, and antagonism are representatively set at levels ± 20% of the additive line to distinguish clearer effects of synergy or antagonism.

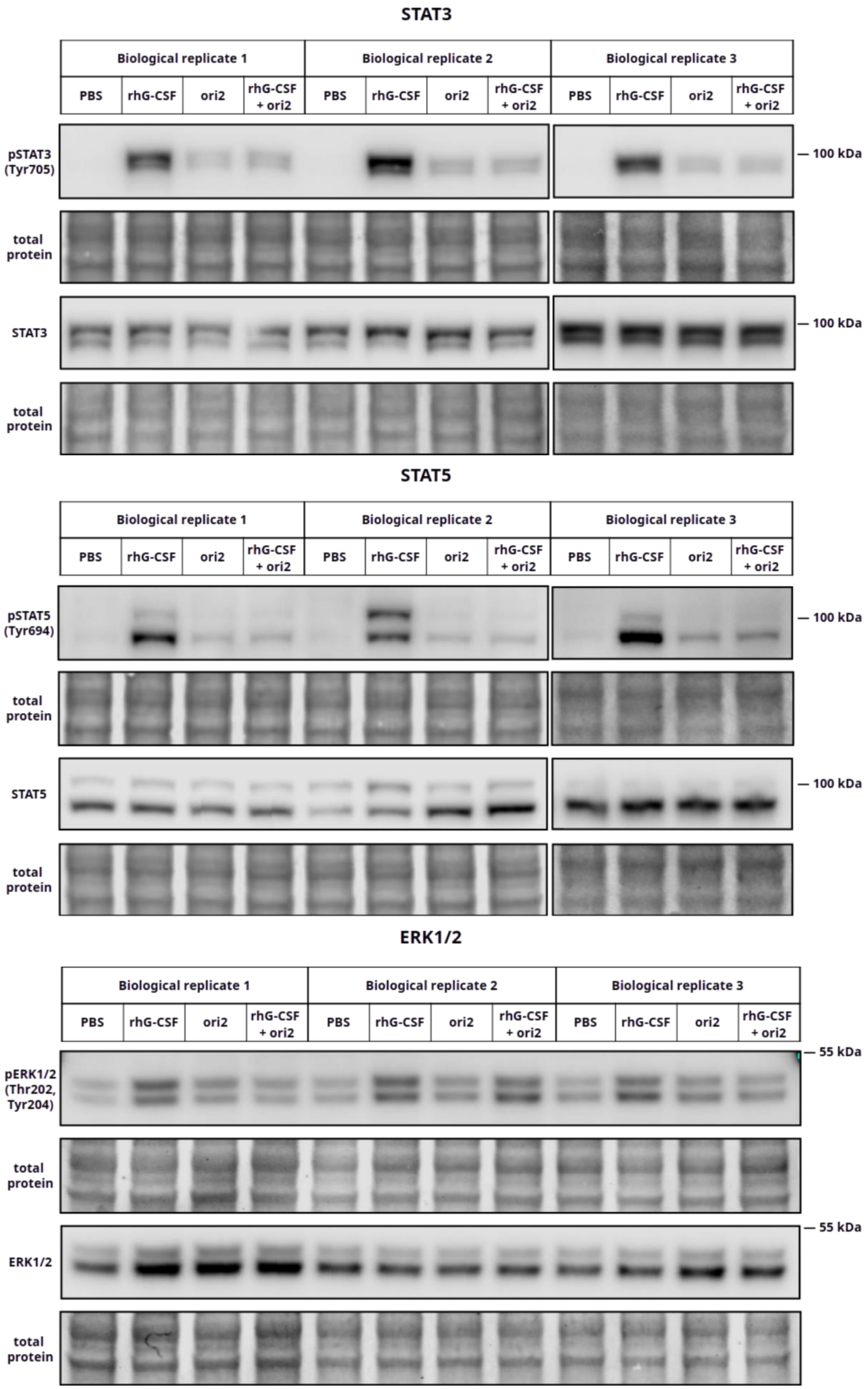
STAT3
Biological replicate 1
Biological replicate 2
Biological replicate 3
PBS
rhG-CSF
ori2
rhG-CSF + ori2
pSTAT3 (Tyr705)
total protein
STAT3
total protein
— 100 kDa
— 100 kDa
STAT5
Biological replicate 1
Biological replicate 2
Biological replicate 3
PBS
rhG-CSF
ori2
rhG-CSF + ori2
pSTAT5 (Tyr694)
total protein
STAT5
total protein
— 100 kDa
— 100 kDa
ERK1/2
Biological replicate 1
Biological replicate 2
Biological replicate 3
PBS
rhG-CSF
ori2
rhG-CSF + ori2
pERK1/2 (Thr202, Tyr204)
total protein
ERK1/2
total protein
— 55 kDa
— 55 kDa

Figure S20. Treatment with ori2 downregulates G-CSF-induced secondary messenger phosphorylation. The figure shows Western blot analysis of phosphorylated and total STAT3 (top), STAT5 (middle), and ERK1/2 (bottom) in NFS-60 cells treated with either PBS buffer, rhG-CSF (26 pM), ori2 (340 nM), or a mixture of rhG-CSF (26 pM) and ori2 (340 nM), respectively. Stained total protein bands were used for normalizing the relative quantification of phosphoprotein levels shown in Fig. 4H-J. Results were obtained from three biologically-independent replicas.

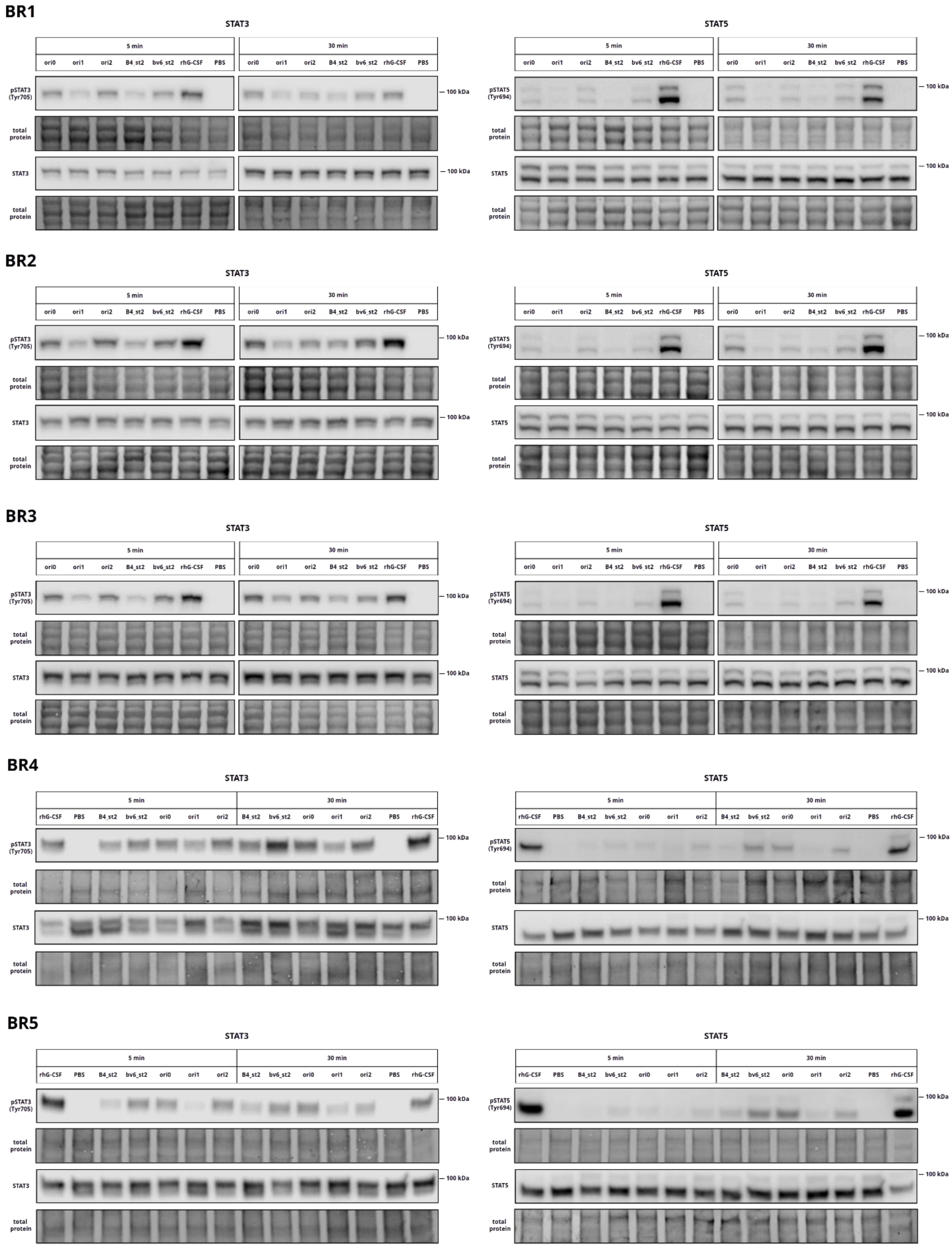

Figure S21. Western Blot analysis of extracts from NFS-60 cells treated with B4_st2, bv6_st2, ori0, ori1, ori2 designs, PBS, or rhG-CSF for 5 or 30 min, using phospho-STAT3 (Tyr705) or phospho-STAT5

(Tyr694) rabbit antibodies (as presented in Fig. 5A-D). As loading control, total protein staining of the corresponding lanes was used. BR1-5 indicate data from five independent biological replicas.

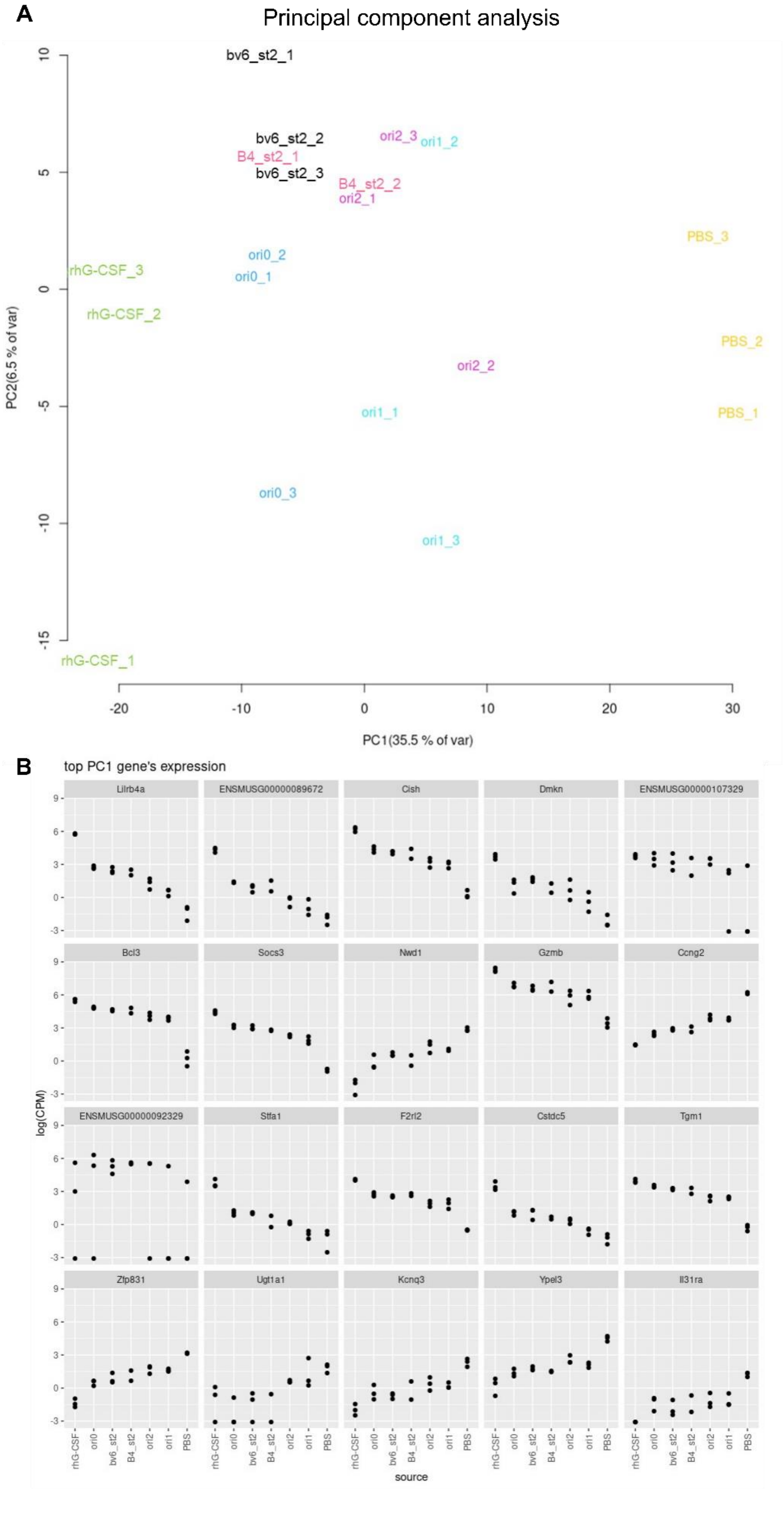
A
Principal component analysis
bv6_st2_1
bv6_st2_2
B4_st2_1
bv6_st2_3
ori2_3
ori1_2
B4_st2_2
ori2_1
PBS_3
ori0_2
ori0_1
rhG-CSF_3
rhG-CSF_2
PBS_2
ori2_2
ori1_1
PBS_1
ori0_3
ori1_3
rhG-CSF_1
PC2(6.5 % of var)
PC1(35.5 % of var)
B
top PC1 gene's expression
Lilrb4a
ENSMUSG00000089672
Cish
Dmkn
ENSMUSG00000107329
Bcl3
Socs3
Nwd1
Gzmb
Ccng2
ENSMUSG00000092329
Stfa1
F2rl2
Cstdc5
Tgm1
Zfp831
Ugt1a1
Kcnq3
Ypel3
Il31ra
log(CPM)
rhG-CSF
ori0
bv6_st2
B4_st2
ori2
ori1
PBS
source

Figure S22. (A) Principal component analysis (PCA) of groups treated with rhG-CSF, agonist designs (B4_st2, bv6_st2, ori0, ori1, ori2) or PBS and **(B)** the top 20 differentially expressed genes in PC1 of PCA.

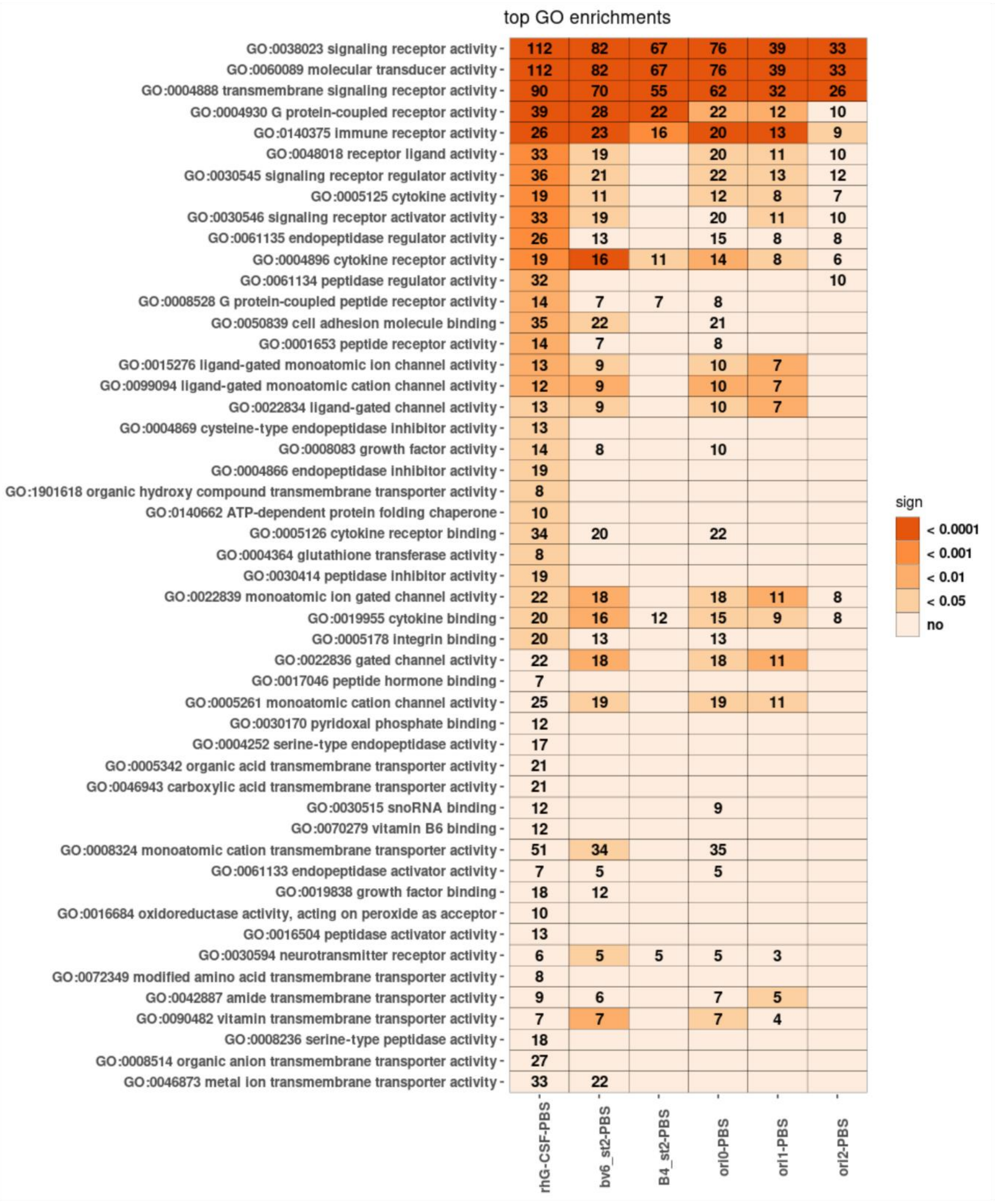

Figure S23. Top 50 GO pathways in the groups treated with rhG-CSF or agonist designs (B4_st2, bv6_st2, ori0, ori1, ori2) compared to PBS.

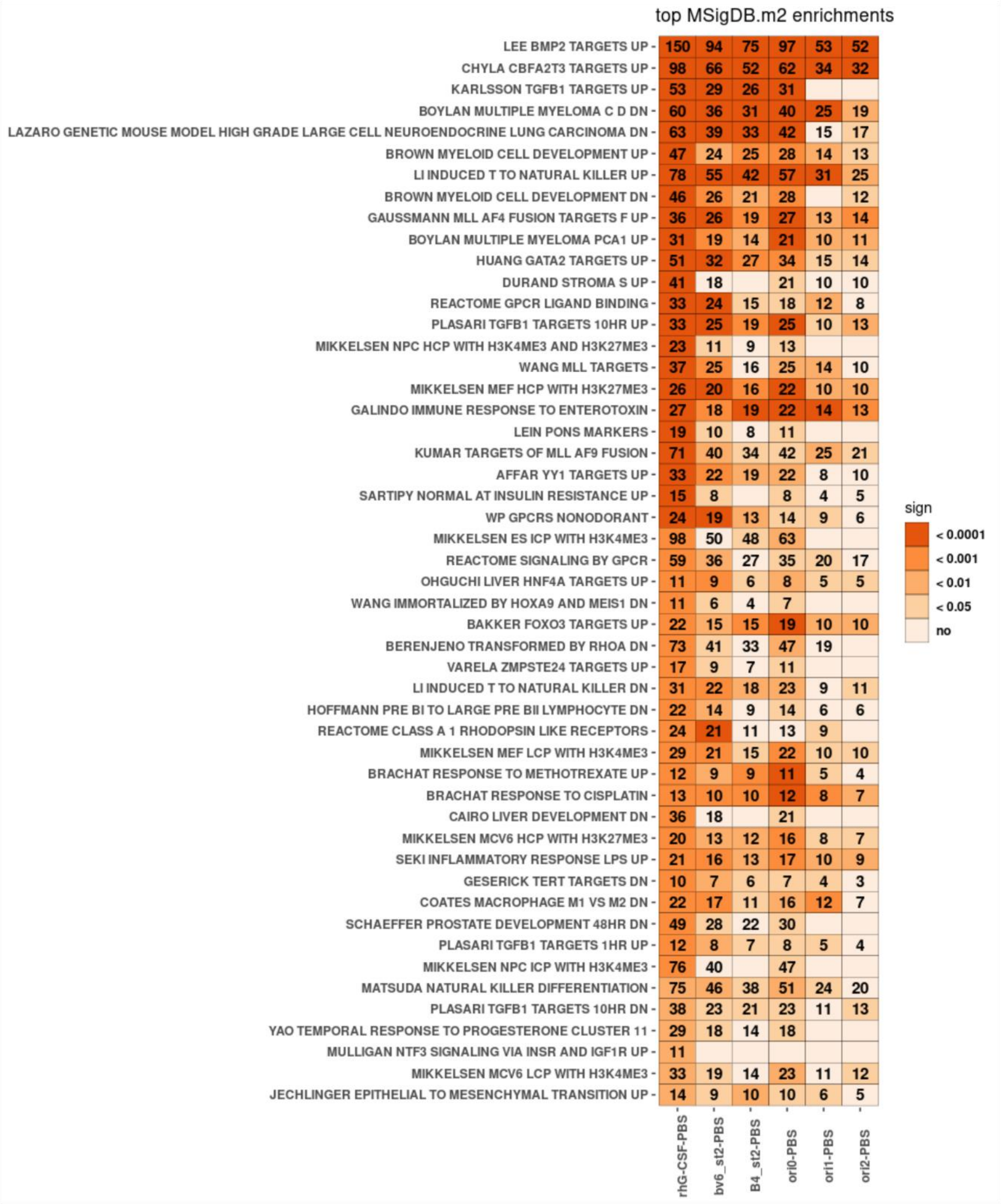

Figure S24. Top 50 MSigDB.m2 pathways in the groups treated with rhG-CSF or agonist designs (B4_st2, bv6_st2, ori0, ori1, ori2) compared to PBS.

Supplemental tables

Table S1. RNA-seq gene counts data.

Table S2. Primers used in this study.

Number	Name	Sequence
1	pN_insert_SfiI/BglI_F	gtaaggcccagccggccatggcagcactggcagcagcactggcagaaatttac
2	pN_insert_NotI_R	gtaagcggccgctgctgctgccagatgacgcaggatctg
3	B4_lib_sat_F	ggtctggatagtctgccgcagNDTtttctgNDTaaagcgctggaaVDGattcgtaaaattcaggcggacgcagcag
4	B4_lib_sat_R	ttcttccattgcctgcgccatANNANNggcaaaatctgccatatcAHNacgcagagcgtccagtgccggac
5	B4_lib_des_F1	ggtctggatagtctgccgcagNDCtttctgYNCaaagcgctggaaMWAattcgtaaaattcaggcggacgcagcag
6	B4_lib_des_F2	ggtctggatagtctgccgcagNDCtttctgYNCaaagcgctggaaRTGattcgtaaaattcaggcggacgcagcag
7	B4_lib_des_R1	ttcttccattgcctgcgccatGTYGNDggcaaaatctgccatatcTMGacgcagagcgtccagtgccggac
8	B4_lib_des_R2	ttcttccattgcctgcgccatTRWGNDggcaaaatctgccatatcTMGacgcagagcgtccagtgccggac
9	B4_lib_des_R3	ttcttccattgcctgcgccatGTYGNDggcaaaatctgccatatcGWWacgcagagcgtccagtgccggac
10	B4_lib_des_R4	ttcttccattgcctgcgccatTRWGNDggcaaaatctgccatatcGWWacgcagagcgtccagtgccggac
11	pET28_B4var_F	GGCAGCCATATGGCAGCACTGGCAGCAG
12	pET28_B4var_R	GTGGTGCTCGAGTTATGCTGCTGCCAGATGACGC
13	st2_backbone_F	taactcgagcaccaccaccaccaccactga
14	st2_backbone_R	atggctgccgcgcggcac
15	st2_fr1_F	AGCAGCGGCCTGGTGCCGCGCGGCAGCCATatggcagcactggcagcagcactggcagaaatttac
16	st2_fr1_R	GCTGGATCCGCCACCGCCtgctgctgccagatgacgcaggatctg
17	st2_fr2_F	GGCGGTGGCGGATCCAGCatggcagcactggcagcagcactggcagaaatttac
18	st2_fr2_R	TCAGTGGTGGTGGTGGTGGTGCTCGAGTTAtgctgctgccagatgacgcaggatctg
19	pN_Seq_insert_F	gaatggtcaagttgtcgac
20	pN_Seq_insert_R	Gaagaaagcgaaaggagc
21	ori1_fr1_R	CATCCAGCGCCACCAGCGTTTGCGTGCCAGATGACGCAGGATCTGG
22	ori1_fr2_F	CGCTGGTGGCGCTGGATGCTGCGCAAAGCACTGGCAGAAATTTACAAAGGTCTGGC
23	ori2_fr1_R	TTCGCGTTTTTTTTTCATGCGTTTCGCAAAGCGACGCAGGATCTGGTACGCTTTTTCCAG
24	ori2_fr2_F	ATGAAAAAAAAACGCGAATGGATGGCGAAAACCCTGGCAGAAATTTACAAAGGTCTGGCGGAATATC
23*	ori2_fr1_fix_R*	CTTCTTCATGCGTTTCGCAAAGCGACG
24*	ori2_fr2_fix_F*	AAACGCGAATGGATGGCGAAAACC
25	ori3_fr1_R	CCATTTTTTGCGCATGCGGCGCGCCAGCTGACGCAGGATCTGGTACGCTTTTTCCAG
26	ori3_fr2_F	CGCATGCGCAAAAAATGGGCGCGCGAAATGATGGCAGCACTGGCAGAAATTTACAAAGGTCTGG
27	ori4_fr1_R	CGCCATCATTTTGCGCATCATGCGGCGCGCCAGGCGACGCAGGATCTGGTACGCTTTTTCCAG
28	ori4_fr2_F	ATGCGCAAAATGATGGCGGAACGCATGGCGCGCGCACTGGCAGAAATTTACAAAGGTCTGGC
29	ori0_fr1_R	CCATTTCATCCACCACCATGCTGCCAGATGACGCAGGATC
30	ori0_fr2_F	TGGTGGTGGATGAAATGGCTGGCAGCAGCACTGGCAGAAATTTAC

* designated primers were used to generate amplicons for blunt-end ligation to reduce redundancy in the construct generated from the preceding ori2 plasmid pair.

Table S3: Composition of PCR reaction mix used for all reactions with the following specifications; for all reactions 5 ng/µL template was inputted but for the ones whose products were used for assembly reactions, 0.3 ng/mL were inputted. In the case of the use of multiple primers for the library backbone PCR, all F or R primers of each construct were mixed at a total concentration of 10 µM (e.g. 2.5 µM of primer 7 to 10 each). PCR program was used for all reactions either with 60 seconds extension time for all insert fragments or 340 seconds for all backbone fragments.

**PCR reaction mix**	Volume (µL)
dNTPs 10 mM each	1
Template 0.3 ng/µL or 5 ng/µL	1
Primer F 10 µM	2.5
Primer R 10 µM	2.5
5x Q5® Reaction Buffer (#B9027, NEB)	10
GC enhancer	10
Q5® High-Fidelity DNA Polymerase (#M0491S, NEB)	0.5
H_2O ad 50 µl	22.5

**PCR program**	Temperature (°C)	Time (s)	Cycle
Initial denaturation	98	30	1x
Denaturation	98	10	30x
Extension	72	60 or 340	
Final Extension	72	300	1x
Hold	8	-	1x

Table S4: Sequence of the fusion protein between Intimin [2] and Boskar4 coded by pNB4 (colored) and sequences of all Boskar4 variants and designs (black) described in this study

Name	Sequence
Intimin$_{EHEC}$ (1-659)-E_tag-Boskar4-Myc_tag	MITHGCYTRTRHKHKLKKTLIMLSAGLGLFFYVNQNSFANGENYFKLGSDSKLLTHDSYQNRLFYTLKTGETVADLSKSQDINLSTIWSLNKHLYS<br>SESEMMKAAPGQQIILPLKKLPFEYSALPLLGSAPLVAAGGVAGHTNKLTKMSPDVTKSNMTDDKALNYAAQQAASLGSQLQSRSLNGDYAKDTAL<br>GIAGNQASSQLQAWLQHYGTAEVNLQSGNNFDGSSLDFLLPFYDSEKMLAFGQVGARYIDSRFTANLGAGQRFFLPANMLGYNVFIDQDFSGDNTR<br>LGIGGEYWRDYFKSSVNGYFRMSGWHESYNKKDYDERPANGFDIRFNGYLPSYPALGAKLIYEQYYGDNVALFNSDKLQSNPGAATVGVNYTPIPL<br>VTMGIDYRHGTGNENDLLYSMQFRYQFDKSWSQQIEPQYVNELRTLSGSRYDLVQRNNNIILEYKKQDILSLNIPHDINGTEHSTQKIQLIVKSKY<br>GLDRIVWDDSALRSQGGQIQHSGSQSAQDYQAILPAYVQGGSNIYKVTARAYDRNGNSSNNVQLTITVLSNGQVVDQVGVTDFTADKTSAKADNAD<br>TITYTATVKKNGVAQANVPVSFNIVSGTATLGANSAKTDANGKATVTLKSSTPGQVVVSAKTAEMTSALNASAVIFFDGAPVPYPDPLEPAQPAMA<br>ALAAALAEIYKGLAEYQARLKSLEGISPELGPALDALRLDMADFATTMAQAMEEGLDSLPQSFLLKALEQIRKIQADAAALREKLAATYKGNDRAA<br>AAVEIAAQLEAFLEKAYQILRHLAAAAAAEQKLISEEDAAA
**bv1**	MAALAAALAEIYKGLAEYQARLKSLEGISPELGPALDALRYDMADFAYLMAQAMEEGLDSLPQLFLYKALEMIRKIQADAAALREKLAATYKGNDR<br>AAAAVEIAAQLEAFLEKAYQILRHLAAA
**bv2**	MAALAAALAEIYKGLAEYQARLKSLEGISPELGPALDALRYDMADFACFMAQAMEEGLDSLPQIFLYKALEQIRKIQADAAALREKLAATYKGNDR<br>AAAAVEIAAQLEAFLEKAYQILRHLAAA
**bv3**	MAALAAALAEIYKGLAEYQARLKSLEGISPELGPALDALRYDMADFAYFMAQAMEEGLDSLPQGFLHKALEQIRKIQADAAALREKLAATYKGNDR<br>AAAAVEIAAQLEAFLEKAYQILRHLAAA
**bv4**	MAALAAALAEIYKGLAEYQARLKSLEGISPELGPALDALRYDMADFAYFMAQAMEEGLDSLPQIFLLKALEQIRKIQADAAALREKLAATYKGNDR<br>AAAAVEIAAQLEAFLEKAYQILRHLAAA
**bv5**	MAALAAALAEIYKGLAEYQARLKSLEGISPELGPALDALRFDMADFAVFMAQAMEEGLDSLPQFFLFKALEQIRKIQADAAALREKLAATYKGNDR<br>AAAAVEIAAQLEAFLEKAYQILRHLAAA
**bv6**	MAALAAALAEIYKGLAEYQARLKSLEGISPELGPALDALRYDMADFAILMAQAMEEGLDSLPQSFLRKALEMIRKIQADAAALREKLAATYKGNDR<br>AAAAVEIAAQLEAFLEKAYQILRHLAAA
**bv7**	MAALAAALAEIYKGLAEYQARLKSLEGISPELGPALDALRFDMADFAYFMAQAMEEGLDSLPQSFLYKALEQIRKIQADAAALREKLAATYKGNDR<br>AAAAVEIAAQLEAFLEKAYQILRHLAAA
**bv8**	MAALAAALAEIYKGLAEYQARLKSLEGISPELGPALDALRLDMADFARLMAQAMEEGLDSLPQRFLYKALEMIRKIQADAAALREKLAATYKGNDR<br>AAAAVEIAAQLEAFLEKAYQILRHLAAA
**bv9**	MAALAAALAEIYKGLAEYQARLKSLEGISPELGPALDALRYDMADFAIYMAQAMEEGLDSLPQHFLLKALEQIRKIQADAAALREKLAATYKGNDR<br>AAAAVEIAAQLEAFLEKAYQILRHLAAA
**bv10**	MAALAAALAEIYKGLAEYQARLKSLEGISPELGPALDALRYDMADFAYFMAQAMEEGLDSLPQSFLLKALEQIRKIQADAAALREKLAATYKGNDR<br>AAAAVEIAAQLEAFLEKAYQILRHLAAA
**bv11**	MAALAAALAEIYKGLAEYQARLKSLEGISPELGPALDALRHDMADFAYFMAQAMEEGLDSLPQGFLDKALEQIRKIQADAAALREKLAATYKGNDR<br>AAAAVEIAAQLEAFLEKAYQILRHLAAA
**bv12**	MAALAAALAEIYKGLAEYQARLKSLEGISPELGPALDALRYDMADFALFMAQAMEEGLDSLPQCFLLKALEQIRKIQADAAALREKLAATYKGNDR<br>AAAAVEIAAQLEAFLEKAYQILRHLAAA
**bv13**	MAALAAALAEIYKGLAEYQARLKSLEGISPELGPALDALRLDMADFAFFMAQAMEEGLDSLPQSFLYKALEQIRKIQADAAALREKLAATYKGNDR<br>AAAAVEIAAQLEAFLEKAYQILRHLAAA
**bv14**	MAALAAALAEIYKGLAEYQARLKSLEGISPELGPALDALRYDMADFAFNMAQAMEEGLDSLPQGFLYKALEQIRKIQADAAALREKLAATYKGNDR<br>AAAAVEIAAQLEAFLEKAYQILRHLAAA
**bv15**	MAALAAALAEIYKGLAEYQARLKSLEGISPELGPALDALRYDMADFAYNMAQAMEEGLDSLPQHFLLKALEMIRKIQADAAALREKLAATYKGNDR<br>AAAAVEIAAQLEAFLEKAYQILRHLAAA
**bv16**	MAALAAALAEIYKGLAEYQARLKSLEGISPELGPALDALRLDMADFATNMAQAMEEGLDSLPQFFLYKALEMIRKIQADAAALREKLAATYKGNDR<br>AAAAVEIAAQLEAFLEKAYQILRHLAAA
**bv1_st2**	MAALAAALAEIYKGLAEYQARLKSLEGISPELGPALDALRYDMADFAYLMAQAMEEGLDSLPQLFLYKALEMIRKIQADAAALREKLAATYKGNDR<br>AAAAVEIAAQLEAFLEKAYQILRHLAAAGGGGSSMAALAAALAEIYKGLAEYQARLKSLEGISPELGPALDALRYDMADFAYLMAQAMEEGLDSLP<br>QLFLYKALEMIRKIQADAAALREKLAATYKGNDRAAAAVEIAAQLEAFLEKAYQILRHLAAA
**bv2_st2**	MAALAAALAEIYKGLAEYQARLKSLEGISPELGPALDALRYDMADFACFMAQAMEEGLDSLPQIFLYKALEQIRKIQADAAALREKLAATYKGNDR<br>AAAAVEIAAQLEAFLEKAYQILRHLAAAGGGGSSMAALAAALAEIYKGLAEYQARLKSLEGISPELGPALDALRYDMADFACFMAQAMEEGLDSLP<br>QIFLYKALEQIRKIQADAAALREKLAATYKGNDRAAAAVEIAAQLEAFLEKAYQILRHLAAA
**bv6_st2**	MAALAAALAEIYKGLAEYQARLKSLEGISPELGPALDALRYDMADFAILMAQAMEEGLDSLPQSFLRKALEMIRKIQADAAALREKLAATYKGNDR<br>AAAAVEIAAQLEAFLEKAYQILRHLAAAGGGGSSMAALAAALAEIYKGLAEYQARLKSLEGISPELGPALDALRYDMADFAILMAQAMEEGLDSLP<br>QSFLRKALEMIRKIQADAAALREKLAATYKGNDRAAAAVEIAAQLEAFLEKAYQILRHLAAA
**bv8_st2**	MAALAAALAEIYKGLAEYQARLKSLEGISPELGPALDALRLDMADFARLMAQAMEEGLDSLPQRFLYKALEMIRKIQADAAALREKLAATYKGNDR<br>AAAAVEIAAQLEAFLEKAYQILRHLAAAGGGGSSMAALAAALAEIYKGLAEYQARLKSLEGISPELGPALDALRLDMADFARLMAQAMEEGLDSLP<br>QRFLYKALEMIRKIQADAAALREKLAATYKGNDRAAAAVEIAAQLEAFLEKAYQILRHLAAA
**bv15_st2**	MAALAAALAEIYKGLAEYQARLKSLEGISPELGPALDALRYDMADFAYNMAQAMEEGLDSLPQHFLLKALEMIRKIQADAAALREKLAATYKGNDR<br>AAAAVEIAAQLEAFLEKAYQILRHLAAAGGGGSSMAALAAALAEIYKGLAEYQARLKSLEGISPELGPALDALRYDMADFAYNMAQAMEEGLDSLP<br>QHFLLKALEMIRKIQADAAALREKLAATYKGNDRAAAAVEIAAQLEAFLEKAYQILRHLAAA
**bv16_st2**	MAALAAALAEIYKGLAEYQARLKSLEGISPELGPALDALRLDMADFATNMAQAMEEGLDSLPQFFLYKALEMIRKIQADAAALREKLAATYKGNDR<br>AAAAVEIAAQLEAFLEKAYQILRHLAAAGGGGSSMAALAAALAEIYKGLAEYQARLKSLEGISPELGPALDALRLDMADFATNMAQAMEEGLDSLP<br>QFFLYKALEMIRKIQADAAALREKLAATYKGNDRAAAAVEIAAQLEAFLEKAYQILRHLAAA
**ori1**	MAALAAALAEIYKGLAEYQARLKSLEGISPELGPALDALRYDMADFAILMAQAMEEGLDSLPQSFLRKALEMIRKIQADAAALREKLAATYKGNDR<br>AAAAVEIAAQLEAFLEKAYQILRHLARKRWWRWMLRKALAEIYKGLAEYQARLKSLEGISPELGPALDALRYDMADFAILMAQAMEEGLDSLPQSF<br>LRKALEMIRKIQADAAALREKLAATYKGNDRAAAAVEIAAQLEAFLEKAYQILRHLAAA
**ori2**	MAALAAALAEIYKGLAEYQARLKSLEGISPELGPALDALRYDMADFAILMAQAMEEGLDSLPQSFLRKALEMIRKIQADAAALREKLAATYKGNDR<br>AAAAVEIAAQLEAFLEKAYQILRRFAKRMKKKREWMAKTLAEIYKGLAEYQARLKSLEGISPELGPALDALRYDMADFAILMAQAMEEGLDSLPQS<br>FLRKALEMIRKIQADAAALREKLAATYKGNDRAAAAVEIAAQLEAFLEKAYQILRHLAAA

**ori3**	MAALAAALAEIYKGLAEYQARLKSLEGISPELGPALDALRYDMADFAILMAQAMEEGLDSLPQSFLRKALEMIRKIQADAAALREKLAATYKGNDRAAAAVEIAAQLEAFLEKAYQILRQLARRMRKKWAREMMAALAEIYKGLAEYQARLKSLEGISPELGPALDALRYDMADFAILMAQAMEEGLDSLPQSFLRKALEMIRKIQADAAALREKLAATYKGNDRAAAAVEIAAQLEAFLEKAYQILRHLAAA
**ori4**	MAALAAALAEIYKGLAEYQARLKSLEGISPELGPALDALRYDMADFAILMAQAMEEGLDSLPQSFLRKALEMIRKIQADAAALREKLAATYKGNDRAAAAVEIAAQLEAFLEKAYQILRRLARRMMRKMMAERMARALAEIYKGLAEYQARLKSLEGISPELGPALDALRYDMADFAILMAQAMEEGLDSLPQSFLRKALEMIRKIQADAAALREKLAATYKGNDRAAAAVEIAAQLEAFLEKAYQILRHLAAA
**ori0**	MAALAAALAEIYKGLAEYQARLKSLEGISPELGPALDALRYDMADFAILMAQAMEEGLDSLPQSFLRKALEMIRKIQADAAALREKLAATYKGNDRAAAAVEIAAQLEAFLEKAYQILRHLAAWWWMKWLAAALAEIYKGLAEYQARLKSLEGISPELGPALDALRYDMADFAILMAQAMEEGLDSLPQSFLRKALEMIRKIQADAAALREKLAATYKGNDRAAAAVEIAAQLEAFLEKAYQILRHLAAA

Supplemental methods

Damietta specifications for the design of orientation-rigging ligands

Example Damietta specifications for weights, sample parameters, the mutable residues, and repackable residues for designing the rigid linker helix region of ori0 (compare Material and Methods, Design of orientation-rigging ligands):

```
# mutational targets
mut_res 124 ADEFHIKLMNQRSTVWY
mut_res 125 ADEFHIKLMNQRSTVWY
mut_res 126 ADEFHIKLMNQRSTVWY
mut_res 127 ADEFHIKLMNQRSTVWY
mut_res 128 KREQ
mut_res 129 ADEFHIKLMNQRSTVWY

# repacking targets
rpk_res 61
rpk_res 63
rpk_res 66
rpk_res 119
rpk_res 120
rpk_res 121
rpk_res 122
rpk_res 123
rpk_res 130
rpk_res 131
rpk_res 132
rpk_res 133
rpk_res 134
rpk_res 184
rpk_res 185
rpk_res 247
rpk_res 250

# sampling parameters (optional)
scramble_order       1 # default:= 0
m_mutations               3 # default:= 3
n_paths                   7 # default:= 1
n_iters                   5 # default:= 1

# mutagenesis scoring weights (optional)
mut_max_lj                25.0
mut_w_pp             1.0
mut_w_k              0.0
mut_w_lj             1.0
mut_w_solv                1.0
mut_w_elec                0.125

# repacking scoring weights (optional)
rpk_max_lj           25.0
rpk_w_pp        1.0
rpk_w_k         1.0
```

```
rpk_w_lj         1.0
rpk_w_solv            1.0
rpk_w_elec            0.125
```

References

1. Young, D.C., Cheng, Q.L., Hou, J., Matthews, D.J., and Zhan, H. (1997). Characterization of the receptor binding determinants of granulocyte colony stimulating factor. Protein Science *6*, 1228-1236.
2. Salema, V., Marín, E., Martínez-Arteaga, R., Ruano-Gallego, D., Fraile, S., Margolles, Y., Teira, X., Gutierrez, C., Bodelón, G., and Fernández, L.Á. (2013). Selection of single domain antibodies from immune libraries displayed on the surface of E. coli cells with two β-domains of opposite topologies. PloS one *8*, e75126. 10.1371/journal.pone.0075126.
